# Stable Two‐Legged Parent Piano‐Stool and Mixed Diborabenzene‐E_4_ (E=P, As) Sandwich Complexes of Group 8

**DOI:** 10.1002/anie.202206840

**Published:** 2022-07-27

**Authors:** Maximilian Dietz, Merle Arrowsmith, Stephan Reichl, Leonardo I. Lugo‐Fuentes, J. Oscar C. Jiménez‐Halla, Manfred Scheer, Holger Braunschweig

**Affiliations:** ^1^ Institute for Inorganic Chemistry Julius-Maximilians-Universität Würzburg Am Hubland 97074 Würzburg Germany; ^2^ Institute for Sustainable Chemistry & Catalysis with Boron Julius-Maximilians-Universität Würzburg Am Hubland 97074 Würzburg Germany; ^3^ Institute of Inorganic Chemistry University of Regensburg Universitätsstraße 31 93040 Regensburg Germany; ^4^ Departamento de Química, División de Ciencias Naturales y Exactas Unversidad de Guanajuato, Noria Alta S/N Col. Noria Alta Guanajuato, C.P. 36050, Gto. Mexico

**Keywords:** 1,4-Diborabenzene, Bonding, Group 8 Metals, Parent Piano-Stool Complex, Pnictogen Reduction

## Abstract

A cyclic alkyl(amino)carbene‐stabilized 1,4‐diborabenzene (DBB) ligand enables the isolation of 18‐electron two‐legged parent piano‐stool Fe^0^ and Ru^0^ complexes, [(η^6^‐DBB)M(CO)_2_], the ruthenium complex being the first of its kind to be structurally characterized. [(η^6^‐DBB)Fe(CO)_2_] reacts with E_4_ (E=P, As) to yield mixed DBB‐*cyclo*‐E_4_ sandwich complexes with planar E_4_^2−^ ligands. Computational analyses confirm the strong electron‐donating capacity of the DBB ligand and show that the E_4_ ligand is bound by four equivalent Fe−P σ bonds.

## Graphical abstract

A cyclic alkyl(amino)carbene‐stabilized 1,4‐diborabenzene ligand enables the isolation of thermally stable two‐legged parent piano‐stool Fe^0^ and Ru^0^ complexes. The iron complex reacts with P_4_ and As_4_ to yield mixed diborabenzene‐*cyclo*‐E_4_ sandwich complexes with planar E_4_^2−^ ligands. Computational analyses confirm the strong electron‐donating capacity of the DBB ligand and show that the E_4_ ligand is bound by four equivalent Fe−P σ bonds.

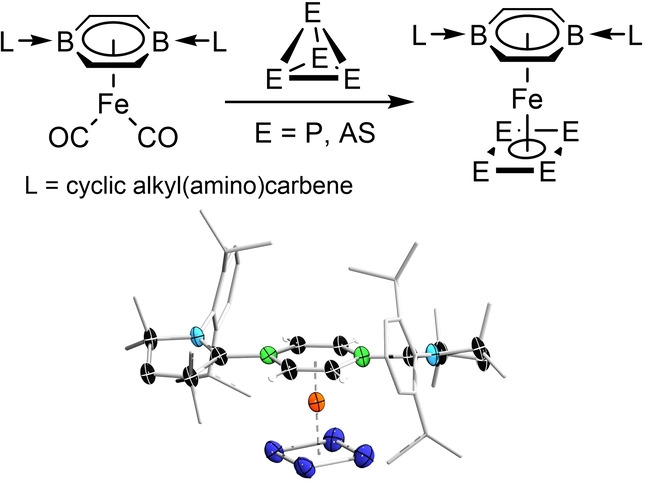

Owing to their labile CO ligands low‐valent half‐sandwich transition metal carbonyl complexes, or parent piano‐stool complexes,[^1^, ^2^, ^3^] are ideal precursors for a variety of half‐sandwich and mixed ligand sandwich complexes used in organometallic chemistry and catalysis.[^4^, ^5^, ^6^, ^7^, ^8^, ^9^, ^10^, ^11^, ^12^] Two‐legged parent piano‐stool 18‐electron complexes, [(η^*n*^‐C_*n*_R_*n*_)M(CO)_2_] (*n*=3–8), are relatively rare compared to their three‐ and four‐legged counterparts. Often generated in situ by photolysis of tricarbonyl precursors,[^13^, ^14^] they tend to dimerize via metal‐metal bonding and/or CO bridging.[^15^, ^16^, ^17^, ^18^, ^19^, ^20^] Bursten showed that the stability of these complexes is mainly governed by the relative energy of the HOMO versus the LUMO and HOMO‐1.^[21]^ Their relative instability often results from insufficient stabilization of the metal‐centered HOMO by the π‐acidic aromatic ligand.[^22^, ^23^]

There are only a handful of zero‐valent two‐legged parent piano‐stool group 8 complexes. Most of these are highly reducing anionic Fe^0^ and Ru^0^ cyclopentadienyl derivatives, often stabilized by additional CO⋅⋅⋅countercation interactions.[^24^, ^25^, ^26^, ^27^, ^28^, ^29^, ^30^, ^31^, ^32^] While [(η^6^‐arene)M(CO)_2_] (M=Fe, Ru, Os) complexes such as **I‐M**, **II** and **III^R^‐X** (Figure 1) have long been known,[^33^, ^34^, ^35^, ^36^] only the iron complex **III^Me^‐SiMe_3_** has been structurally authenticated.^[36]^

**Figure 1:**
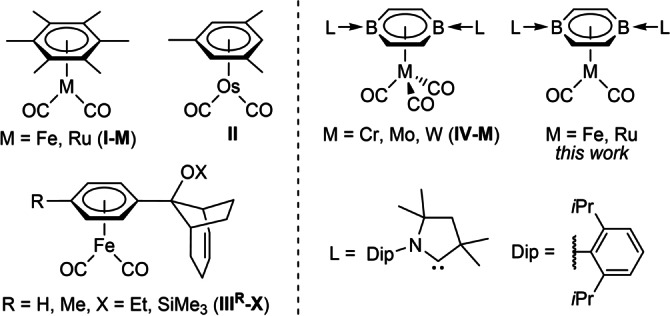
Known neutral two‐legged parent piano‐stool group 8 arene complexes (**I**–**III**) and parent piano‐stool 1,4‐diborabenzene (DBB) group 6 (**IV**) and group 8 complexes.

The substitution of endocyclic carbon atoms by more electropositive boron atoms in a benzene ring results in a removal of degeneracy of the highest filled molecular orbitals (MOs) and a destabilization of the MOs involving the boron 2p orbitals.[^37^, ^38^, ^39^, ^40^, ^41^, ^42^] This makes borabenzenes significantly more electron‐donating than their isoelectronic organic counterparts. Our group has reported the synthesis and coordination chemistry of the doubly cyclic alkyl(amino)carbene (CAAC)‐stabilized 1,4‐diborabenzene (DBB) **1** (Scheme 1).[^43^, ^44^, ^45^] Three‐legged parent piano‐stool group 6 DBB complexes (**IV‐M**, Figure 1) displayed the lowest CO stretching frequencies yet observed for this class of complex, reflecting the exceptionally strong electron‐donating ability of **1**.^[44]^ Furthermore, **1** enabled the synthesis of the first heteroarene actinide complexes.^[45]^ In this work we report the use of **1** as a stabilizing ligand for two‐legged parent piano‐stool group 8 complexes of the form [(η^6^‐DBB)M(CO)_2_] (M=Fe, Ru) and the reduction of white phosphorus and yellow arsenic by the iron complex, yielding mixed‐ligand [(η^6^‐DBB)M(η^4^‐E_4_)] (E=P, As) sandwich complexes.

**Scheme 1:**
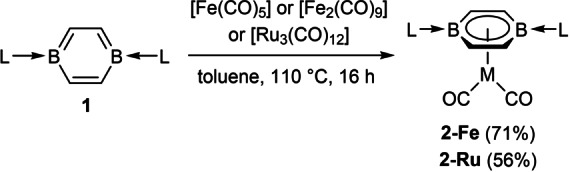
Synthesis of two‐legged parent piano‐stool group 8 diborabenzene complexes.

The two‐legged piano‐stool complexes **2‐Fe** and **2‐Ru** were synthesized by refluxing **1** with [Fe(CO)_5_] or [Fe_2_(CO)_9_] and [Ru_3_(CO)_12_], respectively, in toluene (Scheme 1). The complexes were isolated as dark green and dark turquoise solids, respectively. The analogous reaction with [Os_3_(CO)_12_] did not lead to the formation of **2‐Os**. The ^11^B NMR spectrum of **2‐Fe** and **2‐Ru** complexes shows a broad resonance around 4 ppm, significantly upfield from **1** (*δ*_11B_=24.8 ppm),^[43]^ and similar to the group 6 complexes **IV‐M** (*δ*_11B_=6–7 ppm).^[44]^ The ^1^H and ^13^C NMR DBB ring resonances are significantly upfield‐shifted from 7.3 to ca. 4.7 ppm and from 150 to ca. 108 ppm, respectively, reflecting a reduction in aromaticity. The IR spectra showed several broad CO stretching bands in the 1822–1877 cm^−1^ region for **2‐Fe** and 1843–1978 cm^−1^ for **2‐Ru**, shifted to much lower wavenumbers than in the analogous arene complexes **III^R^‐X** (*ν*_CO_=1877–1980 cm^−1^) and **I‐Ru** (*ν*_CO_=1903, 1973 cm^−1^).[^34^, ^36^] This confirms the exceptionally strong backdonation from the [(DBB)M] fragment into the π* orbitals of the CO ligands previously observed for **IV‐M**.^[44]^

The UV‐Vis spectra of **2‐Fe** and **2‐Ru** in benzene showed absorption maxima at 425 and 358 nm, respectively, as well as broad higher‐wavelength absorption bands in the 570–710 nm region, and two additional low intensity bands at 453 and 494 nm for **2‐Ru**. Calculations on complexes **IV‐M** have shown that the absorption maximum around 400 nm corresponds to π–π* ligand transitions,^[44]^ which undergo a hypsochromic shift upon descending the group, correlating with stronger binding to the metal. The broad bands at lower energy are likely metal‐to‐ligand charge transfer bands.

The solid‐state structures of **2‐Fe** and **2‐Ru** confirm the formation of the two‐legged piano‐stool complexes (Figure 2).^[46]^ While a number of neutral two‐legged piano‐stool Fe^0^ arene complexes have been structurally characterized, including doubly CO‐,^[36]^ NHC‐,^[47]^ silylene‐,^[48]^ stannylene‐,^[49]^ and phosphine‐stabilized examples,[^50^, ^51^, ^52^] **2‐Ru** is, to our knowledge, the first structurally characterized Ru^0^ complex of this type. Unlike in **1** and complexes **IV‐M**,[^43^, ^44^] the DBB ring in **2‐Fe** and **2‐Ru** is slightly twisted out of planarity (B−C−C−B torsion angles: 3.0–5.4°). The CAAC ligands, rotated by 20.8–24.7° out of the C_4_B_2_ plane, adopt a *trans* conformation rather than the *cis* conformation observed in **1** and **IV‐M**. The endocyclic C−C (avg. 1.41 Å) and exocyclic B−C bonds (avg. 1.57 Å) of the DBB ring are slightly longer than in **1** (1.38 and 1.54 Å, respectively),^[43]^ reflecting a decrease in π delocalization within the DBB ring and in π backdonation to the CAAC ligands due to competition with the metal. Whereas the iron center in **2‐Fe** is equidistant from the two boron atoms (Fe1−B1 2.2075(17), Fe1−B2 2.2076(16) Å), the ruthenium atom in **2‐Ru** sits slightly off‐center (Ru1−B1 2.349(3), Ru1−B2 2.317(3) Å). The Fe⋅⋅⋅(DBB)_centroid_ distance (1.59 Å) and Fe−CO bond lengths (avg. 1.74 Å) in **2‐Fe** are identical to those in the arene analogue **III^Me^‐SiMe_3_**.^[36]^ The C−O bonds lengths of **2‐Fe** (1.171(2) and 1.166(2) Å), however, are slightly longer than in **III^Me^‐SiMe_3_** (1.151(2) and 1.159(2) Å), confirming the strong backdonation from the [(DBB)M] fragment suggested by the IR C−O stretching bands.

**Figure 2:**
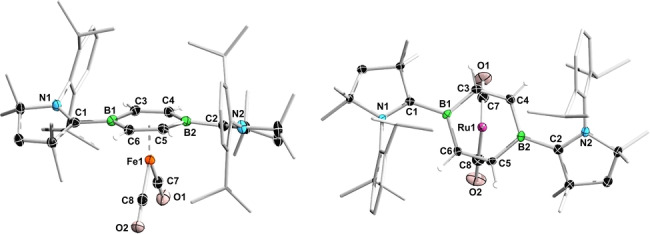
Solid‐state structures of **2‐Fe** (side view) and **2‐Ru** (top view). Atomic displacement ellipsoids at 50 %. Ellipsoids of ligand periphery and CAAC hydrogen atoms omitted for clarity.

The cyclovoltammogram of **2‐Fe** shows a reversible reduction at −2.46 V and a reversible oxidation at −1.13 V (vs Fc/Fc^+^ couple), which correspond to DBB‐based redox events, as the free ligand itself shows two reversible redox events at −2.48 and −0.81 V.^[43]^ Further irreversible reduction and oxidation events are observed at −3.05 and −0.02 V, respectively. The cyclovoltammogram of **2‐Ru** similarly shows four redox waves at −3.19, −2.53, −1.42 and −0.87 V, of which only the reduction at −2.53 V is reversible. Thus the identity of the metal center only influences the oxidation events, so that **2‐Ru** is much less easily oxidized than **2‐Fe**.

Irradiating a benzene suspension of **2‐Fe** and white phosphorus for 4 days under a Hg/Xe UV lamp (185–2000 nm) resulted in quantitative conversion to the mixed sandwich complex **3‐P**, isolated as a dark brown crystalline solid (Scheme 2). The heavier analogue **3‐As** was obtained by refluxing **2‐Fe** with yellow arsenic for 1 h in decalin, and isolated as a dark brown solid. The high reaction temperature of 190 °C did not lead to any substantial decomposition of the **2‐M** precursors, thus demonstrating the stabilizing power of the DBB ligand. Despite the prolonged reaction time for the synthesis of **3‐P** no reaction intermediates could be detected by ^11^B or ^31^P NMR spectroscopy, the reaction proceeding cleanly from **2‐Fe** to **3‐P**. While P_4_ potentially undergoes dissociation to P_2_ under the photolytic conditions employed in the synthesis of **3‐P**,^[53]^ the latter is also formed under the thermal conditions employed in the synthesis of **3‐As**, albeit less efficiently. Since the thermal dissociation of P_4_ to P_2_ and As_4_ to As_2_ require temperatures above 1100 K and 800 K, respectively,[^54^, ^55^] we deem it unlikely that the formation of **3‐E** proceeds via generation of E_2_ fragments.

**Scheme 2:**
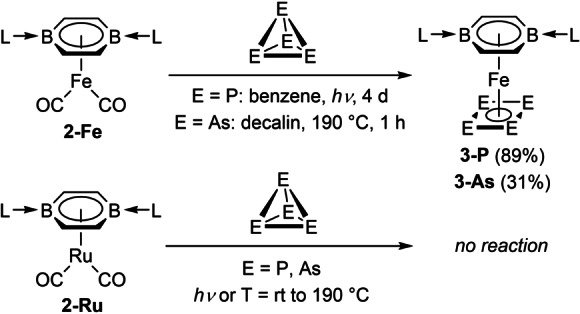
Reduction of P_4_ and As_4_ by **2‐Fe**.

The reduction of the elemental pnictogens was not achieved with **2‐Ru** under similar conditions. The ^11^B NMR shifts of 5.3 and 4.4 ppm for **3‐P** and **3‐As**, respectively, are similar to that of **2‐Fe**. The ^31^P NMR spectrum of **3‐P** displays a broad resonance at 61.4 ppm for the P_4_ ring, downfield‐shifted from that of Mézailles’ related tris(phosphine) iron *cyclo*‐P_4_ complex (*δ*_31P_=53.2 ppm, see complex **4** in Table 3).^[56]^ The ^75^As NMR shift of **3‐As** could not be detected in the entire −1300–2000 ppm range. The UV‐Vis spectra of **3‐P** and **3‐As** each show two major absorption bands in the 380–435 nm region and several broad low intensity bands in the 450–635 nm range.

X‐ray crystallographic analyses of **3‐P** and **3‐As** confirmed the formation of the mixed sandwich complexes (Figure 3). Unlike in **2‐Fe**, the DBB ring is quasi‐planar (B−C−C−B torsion angles<1°). The Fe⋅⋅⋅(DBB)_centroid_ distance (1.55 Å) is slightly shorter than in **2‐Fe**, suggesting a stronger interaction. The Fe⋅⋅⋅(E_4_)_centroid_ distance in **3‐P** (1.77 Å) is only slightly shorter than in **3‐As** (1.79 Å). The E_4_ rings are parallel to the DBB ring and essentially square‐planar (Σ(E−E−E)≈360°). The E−E bonds in **3‐P** (2.139(2)–2.179(2) Å) and **3‐As** (2.383(1)–2.397(1) Å) are within the range of partial double bonds.^[57]^ Complexes with delocalized As_4_ rings are much rarer than their lighter *cyclo*‐P_4_ analogues, both types including several double‐decker iron and cobalt complexes.[^58^, ^59^] The only other structurally characterized neutral end‐deck *cyclo*‐P_4_ iron and end‐deck *cyclo*‐As_4_ complexes are [{κ^3^‐(PhP(CH_2_CH_2_PCy_2_)_2_)}Fe(η^4^‐P_4_)] (see complex **4**, Table 3)^[53]^ and [(η^5^‐C_5_Me_5_)Nb(CO)_2_(η^4^‐As_4_)],^[60]^ respectively, both showing similar E_4_ bonding parameters to **3‐P** and **3‐As**, respectively.

**Figure 3:**
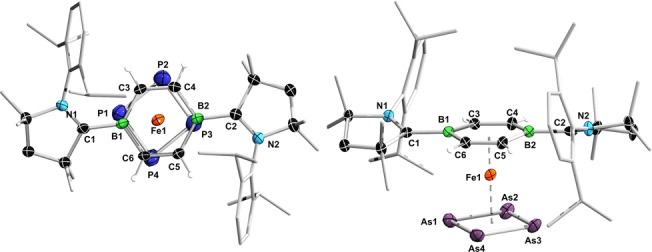
Crystallographically‐determined solid‐state structures of **3‐P** (top view) and **3‐As** (side view). Atomic displacement ellipsoids at 50 %. Ellipsoids of ligand periphery and CAAC hydrogen atoms omitted for clarity.

Given the strong π‐acceptor properties of the DBB ligand, which favor its reduction to [DBB]^2−^,^[44]^ DFT calculations were carried out at the ωB97X−D/def2‐svpp level to investigate the electronic structure of complexes **2‐M** and **3‐E** (see details in the Supporting Information). Bond dissociation energy (BDE) calculations for **2‐M** (M=Fe, Ru, Os) provided the lowest energy values for the neutral singlet fragments, DBB and M(CO)_2_ (Table 1). The calculated net charge of the metal centers is close to neutral and effective oxidation state (EOS)^[61]^ calculations for **2‐Fe** and **2‐Ru** confirm their oxidation state of zero. The HOMO of **2‐M** is located at the metal carbonyl fragment whereas the LUMO is mainly located at the carbene moieties. The HOMO energy of **2‐M** decreases down the group (by only 3 kcal mol^−1^), as do the HOMO–LUMO gaps (**2‐Fe** 5.67 eV, **2‐Os** 5.50 eV). The reason why **2‐Os** is not formed lies in the instability of the Os(CO)_2_ fragment required for its formation. The M→CO π backdonation decreases from M=Fe to Os (Table S2), which in turn weakens the M−CO bonds, thus destabilizing the M(CO)_2_ fragment.

**Table 1:** Frontier MOs, BDEs (corrected by BSSE) and HOMO–LUMO gaps of **2‐M** at the ωB97X−D/def2‐svpp level.

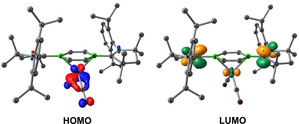				
	**Fragments**	**2‐Fe**	**2‐Ru**	**2‐Os**
BDE [kcal mol^−1^]	DBB+M(CO)_2_	71.0	97.9	109.9
	DBB^2−^+M(CO)_2_^2+^	595.4	667.1	683.4
HOMO–LUMO [eV]		5.67	5.56	5.50

In order to understand the reluctance of **2‐Ru** to react with P_4_ and As_4_, reaction energies were calculated for the formation of **(DBB)ME_4_** from **2‐M** via the dissociation of both CO ligands. Table 2 shows that while the overall energies of formation of **(DBB)RuE_4_** are similar to those of **3‐E**, the dissociation energy for both CO ligands from **2‐Ru** (Δ*G*=119.6 kcal mol^−1^) is a prohibitive 45 kcal mol^−1^ higher than from **2‐Fe** (Δ*G*=74.5 kcal mol^−1^), thus preventing the reaction.

**Table 2:** Calculated reaction energies for the reduction of E_4_ by **2‐M** at the ωB97X‐D/def2‐svpp level.

Reaction	E	Δ*G* [kcal mol^−1^]
**2‐Fe**→(DBB)Fe+2 CO		74.5
(DBB)Fe+E_4_→**3‐E**	P	−124.3
	As	−100.9
**2‐Fe**+E_4_→**3‐E**+2 CO	P	−49.8
	As	−26.4
**2‐Ru**→(DBB)Ru+2 CO		119.6
(DBB)Ru+E_4_→(DBB)RuP_4_	P	−167.7
	As	−146.1
**2‐Ru**+E_4_→(DBB)RuP_4_+2 CO	P	−48.1
	As	−26.4

The electronic nature of **3‐P** and **3‐As** was assessed and compared with complex **4**, a simplified version of the known Fe^II^ complex [{κ^3^‐(PhP(CH_2_CH_2_PCy_2_)_2_)}Fe(η^4^‐P_4_)],^[56]^ in which the cyclohexyl groups were replaced by methyl groups, and the model arene‐P_4_ sandwich complex **5** (Table 3). Calculated net charges at the metal centers and P_4_ ligands and EOS calculations confirm that all four compounds are Fe^II^ complexes with dianionic E_4_ ligands (see Supporting Information). The main differences between **3‐P**, **4** and **5** are seen in the natural bond orbital (NBO) charges summed by fragments.^[62]^ The NBO charge of the DBB fragment of **3‐P** is less positive (+0.193) than that of the tris(phosphine) fragment of **4** (+0.572), indicating that the latter is more electron‐donating. This is in agreement with the downfield‐shift of the ^31^P NMR resonance of **3‐P** (*δ*_31P_=61.4 ppm) compared to [{κ^3^‐(PhP(CH_2_CH_2_PCy_2_)_2_)}Fe(η^4^‐P_4_)] (*δ*_31P_=53.2 ppm).^[56]^ In turn, the DBB ligand of **3‐P** is more electron‐donating than the hexamethylbenzene ligand of **5** (+0.072), as expected upon exchanging two carbon atoms with boron.

**Table 3:** NBO charges of complexes **3‐P**, **4** and **5** summed by fragments.

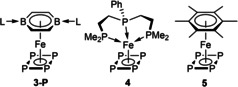			
	**3‐P**	**4**	**5**
Neutral ligand	0.193	0.572	0.074
Fe	0.584	0.215	0.521
P4	−0.777	−0.788	−0.595

The bonding within **3‐P** and **3‐As** was investigated using the NBO approach. Calculations suggest that these complexes have three Fe−E σ bonds (E=P, As) and one lone pair on E, which is strongly delocalized towards the vacant Fe *s* orbital (n(P)→*s*(Fe)), as revealed by the interaction energies (*E*(2)) of 76.1 and 78.8 kcal mol^−1^ for **3‐P** and **3‐As** respectively (Figure 4, see Supporting Information for **3‐As** and for IBO calculations). Due to greater charge transfer from the tris(phosphine) ligand to the metal and P_4_ fragments in **4**, as well as the low *C*_s_ symmetry of the tris(phosphine) ligand, the NBOs of **4** show only two Fe−P σ bonds and a P−P π bond, which allows π backbonding from one metal d orbital into the P−P π* orbital (*E*(2)=68.3 kcal mol^−1^). Complex **5** shows a P−P π bond and two Fe−E σ bonds, albeit with weaker π backbonding (*E*(2)=36.6 kcal mol^−1^) due to the lesser extent of charge transfer from the neutral ligand to the metal and P_4_ fragments. This shows that the bonding situation between the metal center and the E_4_ ligand is strongly affected by the electronics and symmetry of the neutral ligand.

**Figure 4:**
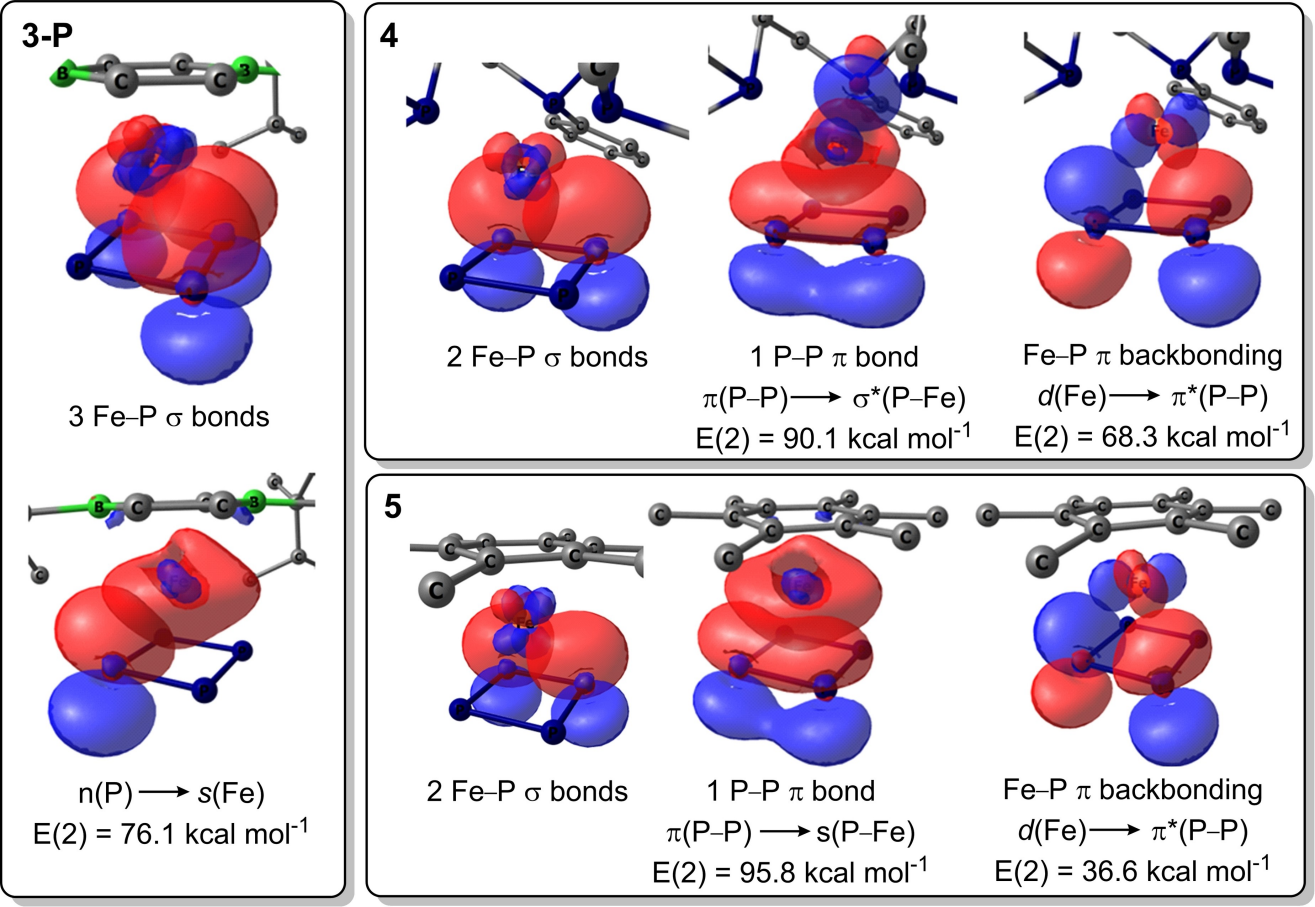
Fe−P_4_ bonding NBOs of complexes **3‐P**, **4** and **5**. Isosurface value: 0.05 a.u.

To conclude, the highly electron‐donating DBB ligand enables the stabilization of rare two‐legged parent Fe^0^ and Ru^0^ piano‐stool complexes, [(η^6^‐DBB)M(CO)_2_] with high thermal stability. The ruthenium complex is the first of its kind to be crystallographically characterized. The reduction of white phosphorus or yellow arsenic by the iron complex yields the mixed sandwich complexes [(η^6^‐DBB)Fe(η^4^‐E_4_)], which display square‐planar E_4_^2−^ ligands. Calculations confirm these are Fe^II^ complexes and show that the *cyclo*‐E_4_ ligand is symmetrically bound by four σ bonds. Comparison with related [LFe(η^4^‐E_4_)] complexes shows that Fe−P_4_ bonding is strongly influenced by the nature of the neutral ligand L.

## Conflict of interest

The authors declare no conflict of interest.

## Supporting information

As a service to our authors and readers, this journal provides supporting information supplied by the authors. Such materials are peer reviewed and may be re‐organized for online delivery, but are not copy‐edited or typeset. Technical support issues arising from supporting information (other than missing files) should be addressed to the authors.

Supporting Information

Supporting Information

Supporting Information

Supporting Information

Supporting Information

## Data Availability

The data that support the findings of this study are available from the corresponding author upon reasonable request.
