# Supplementary material for: Stable Two‐Legged Parent Piano‐Stool and Mixed Diborabenzene‐E4 (E=P, As) Sandwich Complexes of Group 8
Source: Angew Chem Int Ed Engl. 2022 Jul 27;61(36):e202206840. doi: 10.1002/anie.202206840 (PMC9540419; doi:10.1002/anie.202206840)
Supplement: Supplementary file 1 — Supporting Information [file ANIE-61-0-s003.pdf]

Angewandte Chemie
Eine Zeitschrift der Gesellschaft Deutscher Chemiker

Supporting Information

Stable Two-Legged Parent Piano-Stool and Mixed Diborabenzene-E_4 (E = P, As) Sandwich Complexes of Group 8

*M. Dietz, M. Arrowsmith, S. Reichl, L. I. Lugo-Fuentes, J. O. C. Jiménez-Halla, M. Scheer, H. Braunschweig**

Supporting Information

Contents

Methods and materials 2

Synthetic procedures 3

NMR spectra of isolated compounds 6

IR spectra 19

High-resolution mass spectrometry 21

UV-vis spectra 25

Cyclic voltammetry 26

X-ray crystallographic data 28

Computational details 30

References 92

Methods and materials

All manipulations were performed either under an atmosphere of dry argon or *in vacuo* using standard Schlenk line or glovebox techniques. Deuterated solvents were dried over molecular sieves and degassed by three freeze-pump-thaw cycles prior to use. All other solvents were distilled and degassed from appropriate drying agents. Both deuterated and non-deuterated solvents were stored under argon over activated 4 Å molecular sieves. Liquid-phase NMR spectra were acquired on a Bruker Avance 500 spectrometer (^{1}H: 500.1 MHz, ^{11}B: 160.5, ^{31}P: 202.5 MHz) or a Bruker Avance Neo I 600 (^{1}H: 600.2 MHz, ^{75}As: 102.8 MHz). Chemical shifts (δ) are reported in ppm and internally referenced to the carbon nuclei (^{13}C{^{1}H}) or residual protons (^{1}H) of the solvent. Heteronuclei NMR spectra are referenced to external standards (^{11}B: $BF_3{\cdot}OEt_2$, ^{31}P: 85% H_3PO_4, ^{75}As: $NaAsF_6$). Resonances are given as singlet (s), doublet (d), triplet (t), septet (sept) or multiplet (m). High-resolution mass spectrometry (HRMS) data were obtained from a Thermo Scientific Exactive Plus spectrometer. Solid-state IR spectra were recorded on a Bruker FT-IR spectrometer ALPHA II inside a glovebox. UV-vis spectra were acquired on a METTLER TOLEDO UV-vis-Excellence UV5 spectrophotometer inside a glovebox. Photoreactions were performed using a LOT-Quantum Design GmbH mercury-xenon vapor lamp (I = 19 A, U = 26 V). Cyclic voltammetry experiments were performed using a Gamry Instruments Reference 600 potentiostat. A standard three-electrode cell configuration was employed using a platinum disk working electrode, a platinum wire counter electrode, and a silver wire, separated by a Vycor tip, serving as the reference electrode. Formal redox potentials are referenced to the ferrocene/ferrocenium ($[Cp_2Fe]^{+/0}$) redox couple by using decamethylferrocene ($[Cp^*_2Fe]$; $E_{1/2} = -0.427$ V in THF) as an internal standard. Tetra(n-butyl)ammonium hexafluorophosphate ($[n\text{Bu}_4\text{N}][\text{PF}_6]$) was employed as the supporting electrolyte. Compensation for resistive losses (*iR* drop) was employed for all measurements.

Solvents and reagents were purchased from Sigma-Aldrich or Alfa Aesar. $[(\text{CAAC})_2(\text{C}_4\text{H}_4\text{B}_2)]$ = DBB (CAAC = 1-(2,6-di*iso*propylphenyl)-3,3,5,5-tetramethylpyrrolidin-2-ylidene)[1] and yellow arsenic (As_4)[2] were synthesized using literature procedures.

Synthetic procedures

Synthesis of [(η^6-DBB)Fe(CO)$_2$], 2-Fe

Route A: DBB (322 mg, 500 μmol) and [$Fe_2(CO)_9$] (200 mg, 540 μmol) were combined in toluene (15 mL) and stirred at 105 °C for 16 h, whereupon the color changed to dark green. The reaction mixture was filtered and all volatiles removed *in vacuo*. The remaining solid was washed with hexane (4 × 5 mL) and dried again, yielding **2-Fe** as a dark green solid (268 mg, 354 μmol, 71%). Single crystals of **2-Fe** suitable for X-ray diffraction analysis were obtained by slow diffusion of hexane into a saturated benzene solution. **Route B:** DBB (200 mg, 310 μmol) was dissolved in benzene and a stock solution of [$Fe(CO)_5$] in benzene (c = 744 μmol mL^{-1}, 500 μL, 372 μmol) was added. After stirring at 105 °C for 16 h the ^{11}B NMR spectrum showed full conversion to **2-Fe**. ^{1}H{^{11}B} NMR (500.1 MHz, C_6D_6, 297 K): δ = 7.14 (t, ^3J = 7.8 Hz, 2H, *p*-Ar*H*), 7.07 (d, ^3J = 7.8 Hz, 4H, *m*-Ar*H*), 4.85 (br s, 4H, DBB-Ar*H*), 2.89 (sept, ^3J = 6.4 Hz, 4H, *i*Pr-C*H*), 1.72 (s, 12H, C(CH_3)$_2$), 1.60 (s, 4H, CH_2), 1.42 (d, ^3J = 6.4 Hz, 12H, *i*Pr-CH_3), 1.14 (d, ^3J = 6.4 Hz, 12H, *i*Pr-CH_3), 0.94 (s, 12H, NC(CH_3)$_2$) ppm. ^{13}C{^{1}H} NMR (125.8 MHz, C_6D_6, 297 K): δ = 223.8 ($C_{carbene}$, identified by HMBC), 146.3 (*o*-Ar*C*), 136.0 (*i*-Ar*C*), 129.5 (*p*-Ar*C*), 125.8 (*m*-Ar*C*), 108.0 (DBB-Ar*C*), 75.4 (N*C*(CH$_3$)$_2$), 54.1 (*C*H$_2$), 52.3 (*C*(CH$_3$)$_2$), 33.2 (C(*C*H$_3$)$_2$), 29.4 (*i*Pr-*C*H), 28.5 (NC(*C*H$_3$)$_2$), 27.6 (*i*Pr-*C*H$_3$), 24.7 (*i*Pr-*C*H$_3$) ppm. *Note: The CO resonances of the carbonyl moieties could not be detected.* ^{11}B NMR (160.5 MHz, C_6D_6, 297 K): δ = 3.7 (br s) ppm. FT-IR (solid-state): $\tilde{\nu}$(CO) = 1980, 1878, 1822 cm^{-1}. HRMS LIFDI for [$C_{46}H_{66}B_2FeN_2O_2$] = [M]: calcd. 756.4654; found 756.4643.

Synthesis of [(η^6-DBB)Ru(CO)$_2$], 2-Ru

DBB (300 mg, 465 μmol) and [$Ru_3(CO)_9$] (99.1 mg, 155 μmol) were combined in toluene (15 mL) and stirred at 105 °C for 16 h, whereupon the color changed to dark turquoise. The reaction mixture was filtered and all volatiles removed *in vacuo*. The remaining solid was washed with hexane (4 × 5 mL) and dried again, yielding **2-Ru** as a dark turquoise solid (210 mg, 262 μmol, 56%). Single crystals of **2-Ru** suitable for X-ray diffraction analysis were obtained by slow diffusion of hexane into a saturated benzene solution. ^{1}H{^{11}B} NMR (500.1 MHz, C_6D_6, 297 K): δ = 7.13 (t, ^3J = 7.7 Hz, 2H, *p*-Ar*H*), 7.05 (d, ^3J = 7.7 Hz, 4H, *m*-Ar*H*), 4.63 (s, 4H, DBB-Ar*H*), 2.88 (sept, ^3J = 6.6 Hz, 4H, *i*Pr-C*H*), 1.69 (s, 12H, C(CH_3)$_2$), 1.60 (s,

4H, CH_2), 1.45 (d, ^3J = 6.6 Hz, 12H, *i*Pr-CH_3), 1.14 (d, ^3J = 6.6 Hz, 12H, *i*Pr-CH_3), 0.93 (s, 12H, NC(CH_3)$_2$) ppm. ^{13}C{^{1}H} NMR (125.8 MHz, C_6D_6, 297 K): δ = 223.4 ($C_{carbene}$, identified by HMBC), 210.5 (*C*O), 146.6 (*o*-Ar*C*), 135.9 (*i*-Ar*C*), 129.5 (*p*-Ar*C*), 125.9 (*m*-Ar*C*), 107.9 (DBB-Ar*C*), 75.0 (N*C*(CH_3)$_2$), 53.9 (*C*H_2), 51.8 (*C*(CH_3)$_2$), 34.8 (C(*C*H_3)$_2$), 29.4 (*i*Pr-*C*H), 28.5 (NC(*C*H_3)$_2$), 28.1 (*i*Pr-*C*H_3), 24.7 (*i*Pr-*C*H_3) ppm. ^{11}B NMR (160.5 MHz, C_6D_6, 297 K): δ = 4.4 (br s) ppm. FT-IR (solid-state): $\tilde{\nu}$(CO) = 1978, 1910, 1843 cm^{-1}. HRMS LIFDI for [$C_{46}H_{66}B_2RuN_2O_2$] = [M]: calcd. 802.4348; found 802.4344.

Synthesis of [(η^6-DBB)Fe(η^4-P_4)], 3-P

In a quartz Schlenk flask, **2-Fe** (40.0 mg, 52.9 μmol) and white phosphorus (39.3 mg, 317 μmol) were combined in benzene (3 mL) and irradiated under stirring for 4 d, whereupon the color changed to red-brown. All volatiles were removed *in vacuo*, the product was extracted with benzene (4 mL) and all volatiles were removed again. The remaining solid was washed with hexane (4 × 5 mL) and dried, yielding **3-P** as a dark brown solid (39.0 mg, 0.047 μmol, 89%). Single crystals of **3-P** suitable for X-ray diffraction analysis were obtained by slow evaporation of a saturated benzene solution. ^{1}H{^{11}B} NMR (500.1 MHz, CD_2Cl_2, 297 K): δ = 7.45 (t, ^3J = 7.7 Hz, 2H, *p*-Ar*H*), 7.27 (d, ^3J = 7.7 Hz, 4H, *m*-Ar*H*), 4.68 (br s, 4H, DBB-Ar*H*), 2.83 (sept, ^3J = 6.6 Hz, 4H, *i*Pr-C*H*), 2.16 (s, 4H, CH_2), 1.70 (s, 12H, C(CH_3)$_2$), 1.40 (s, 12H, NC(CH_3)$_2$), 1.25 (d, ^3J = 6.6 Hz, 12H, *i*Pr-CH_3), 0.80 (d, ^3J = 6.6 Hz, 12H, *i*Pr-CH_3) ppm. ^{13}C{^{1}H} NMR (125.8 MHz, CD_2Cl_2, 297 K): δ = 228.0 ($C_{carbene}$, identified by HMBC), 146.1 (*o*-Ar*C*), 135.3 (*i*-Ar*C*), 129.8 (*p*-Ar*C*), 125.9 (*m*-Ar*C*), 99.0 (DBB-Ar*C*), 78.4 (N*C*(CH_3)$_2$), 53.6 (*C*(CH_3), overlapping with CD_2Cl_2, identified by HMBC), 53.5 (*C*H_2, overlapping with CD_2Cl_2), 31.2 (C(*C*H_3)$_2$), 30.0 (*i*Pr-*C*H), 29.3 (NC(*C*H_3)$_2$), 27.1 (*i*Pr-*C*H_3), 24.9 (*i*Pr-*C*H_3) ppm. ^{11}B NMR (160.5 MHz, CD_2Cl_2, 297 K): δ = 5.3 (br s) ppm. ^{31}P NMR (202.5 MHz, CD_2Cl_2, 297 K): δ = 61.4 (br s) ppm. HRMS LIFDI for [$C_{44}H_{66}P_4B_2FeN_2$] = [M]: calcd. 824.3712; found 824.3692.

Synthesis of [(η^6-DBB)Fe(η^4-As_4)], 3-As

2-Fe (500 mg, 661 μmol) was suspended in decalin (50 mL) and a freshly prepared, saturated solution of yellow arsenic in decalin (300 mL) was added. The mixture was refluxed for 1 h and all volatiles were removed *in vacuo*, any excess yellow arsenic being transformed into insoluble grey arsenic. The remaining solid was suspended in toluene (50 mL), filtered through celite and further extracted with toluene (2 × 50 mL). After removing all volatiles *in vacuo* the

solid was washed with hexane (4 × 5 mL) and dried again, yielding **3-As** as a dark brown solid (202 mg, 202 μmol, 31%). Single crystals of **3-As** suitable for X-ray diffraction analysis were obtained by slow evaporation of a saturated dichloromethane solution at –30 °C. $^{1}H\{^{11}B\}$ NMR (500.1 MHz, CD_2Cl_2, 297 K): δ = 7.44 (t, ^{3}J = 7.8 Hz, 2H, *p*-Ar*H*), 7.26 (d, ^{3}J = 7.8 Hz, 4H, *m*-Ar*H*), 4.61 (br s, 4H, DBB-Ar*H*), 2.85 (sept, ^{3}J = 6.6 Hz, 4H, *i*Pr-C*H*), 2.17 (s, 4H, CH_2), 1.74 (s, 12H, $C(CH_3)_2$), 1.40 (s, 12H, $NC(CH_3)_2$), 1.25 (d, ^{3}J = 6.6 Hz, 12H, *i*Pr-CH_3), 0.80 (d, ^{3}J = 6.6 Hz, 12H, *i*Pr-CH_3) ppm. $^{13}C\{^{1}H\}$ NMR (125.8 MHz, CD_2Cl_2, 297 K): δ = 228.9 ($C_{carbene}$, identified by HMBC), 146.2 (*o*-Ar*C*), 135.6 (*i*-Ar*C*), 129.8 (*p*-Ar*C*), 126.0 (*m*-Ar*C*), 97.3 (DBB-Ar*C*), 78.2 ($NC(CH_3)_2$), 53.9 ($C(CH_3)$, overlapping with CD_2Cl_2, identified by HMBC), 53.6 (CH_2, overlapping with CD_2Cl_2), 31.1 ($C(CH_3)_2$), 30.2 (*i*Pr-*C*H), 29.4 ($NC(CH_3)_2$), 27.1 (*i*Pr-CH_3), 25.0 (*i*Pr-CH_3) ppm. ^{11}B NMR (160.5 MHz, CD_2Cl_2, 297 K): δ = 4.4 (br s) ppm. *Note: the ^{75}As NMR resonance could not be detected*. HRMS LIFDI for $[C_{44}H_{66}As_4B_2FeN_2]$ = [M]: calcd. 1000.1626; found 1000.1596.

.

NMR spectra of isolated compounds

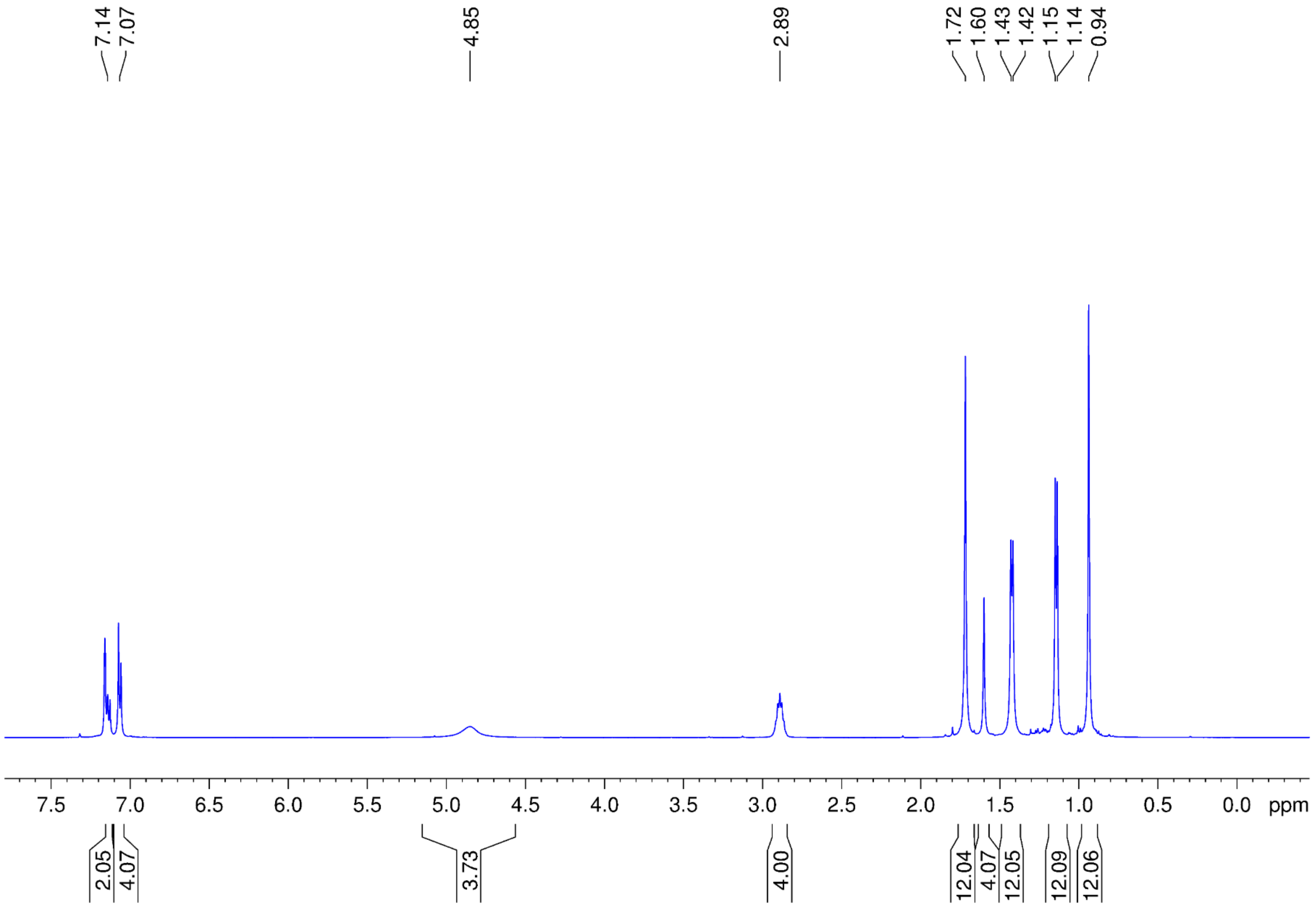

Figure S1. $^{1}H\{^{11}B\}$ NMR spectrum of **2-Fe** in C_6D_6.

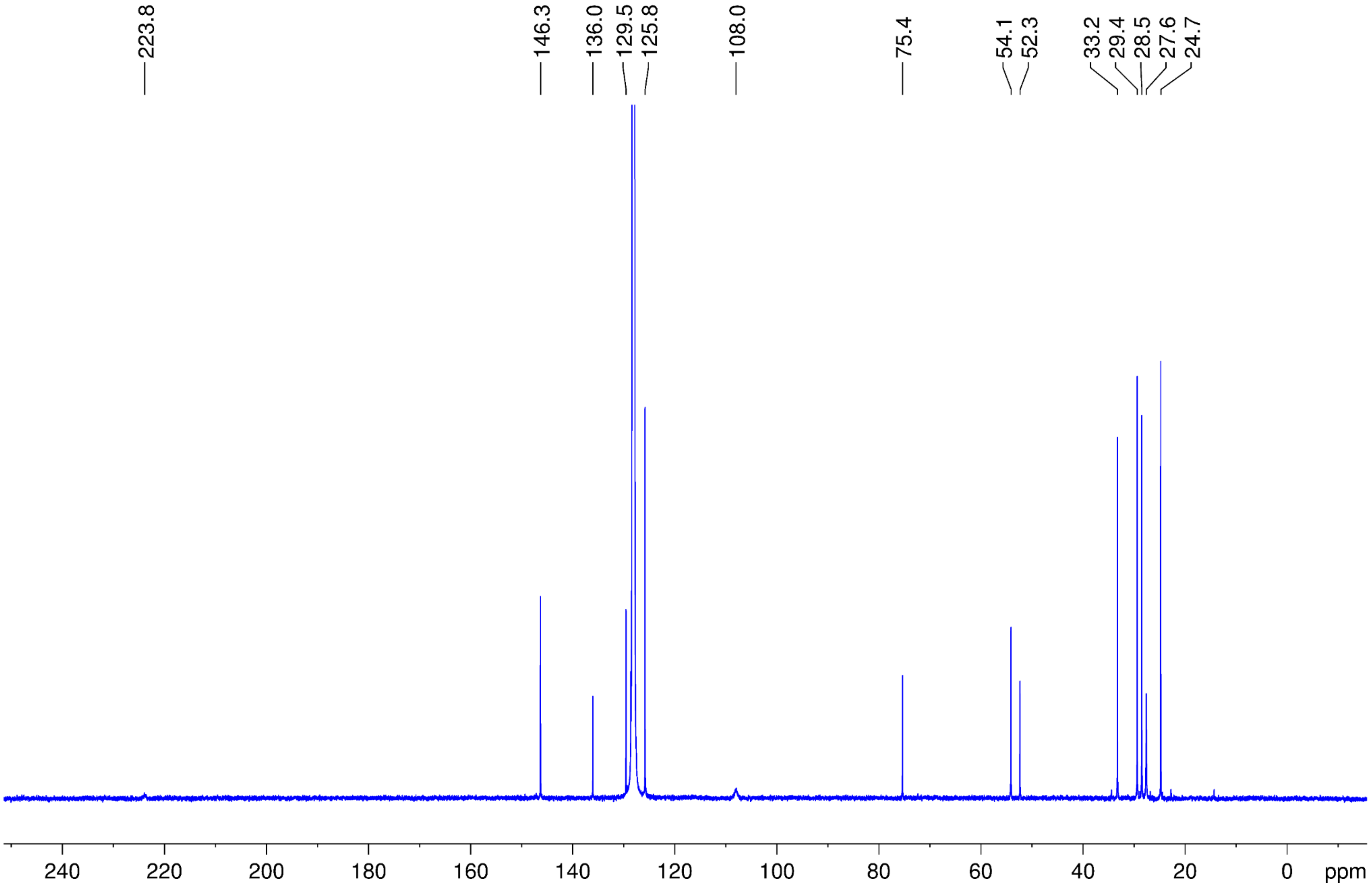

Figure S2. $^{13}C\{^{1}H\}$ NMR spectrum of **2-Fe** in C_6D_6.

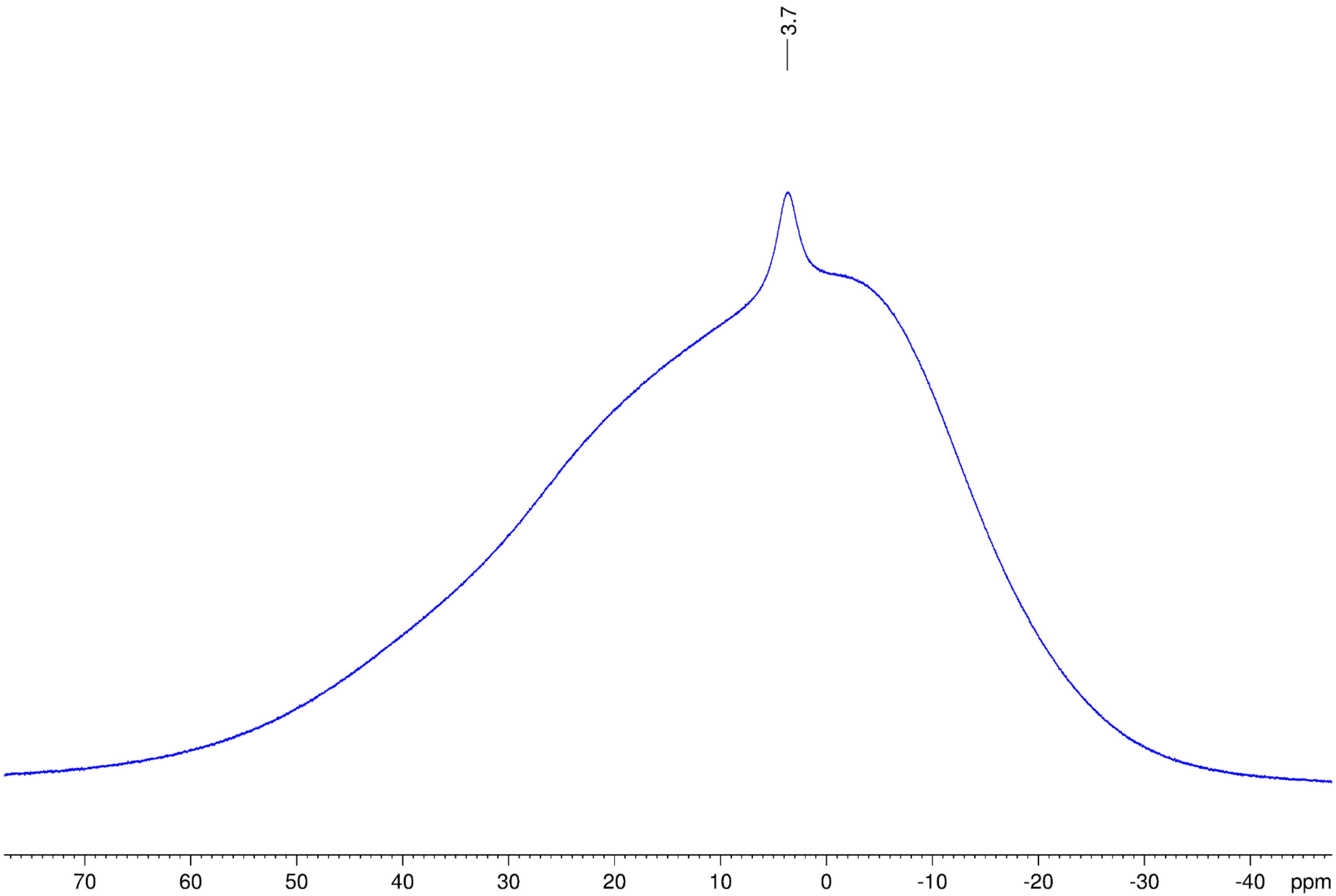

Figure S3. ^{11}B NMR spectrum of **2-Fe** in C_6D_6.

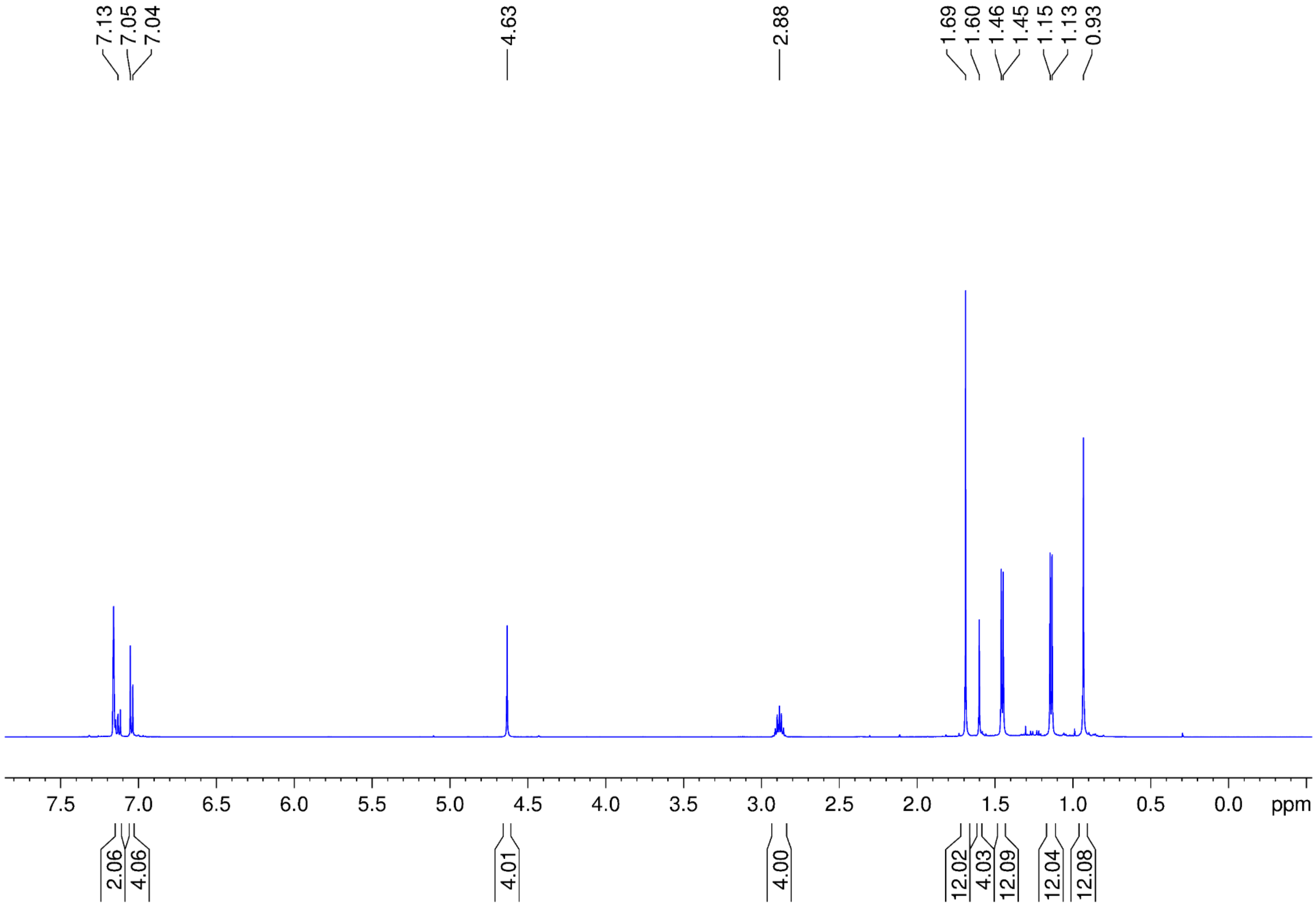

Figure S4. $^{1}H\{^{11}B\}$ NMR spectrum of **2-Ru** in C_6D_6.

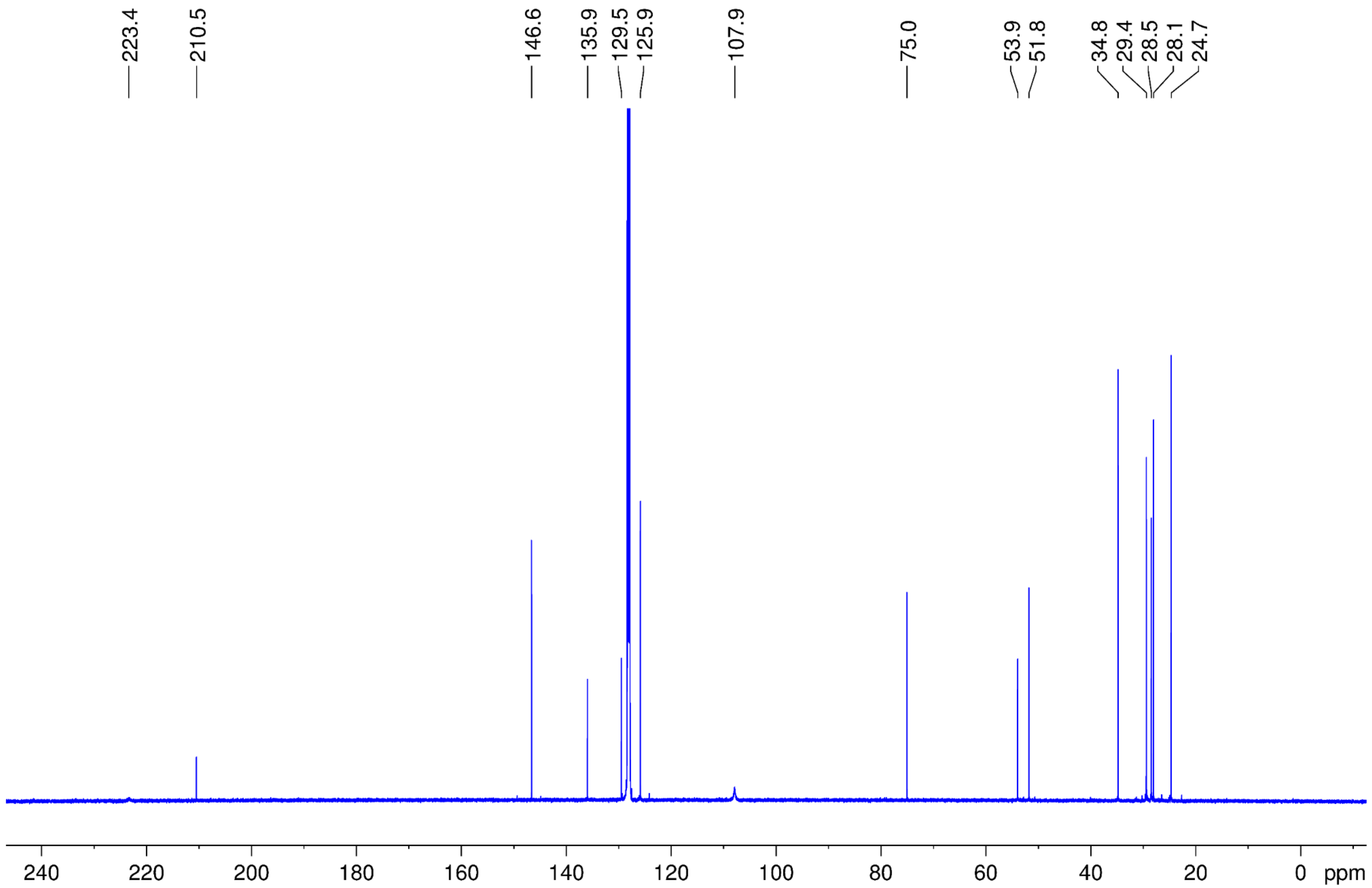

Figure S5. ${}^{13}C\{{}^{1}H\}$ NMR spectrum of **2-Ru** in C_6D_6.

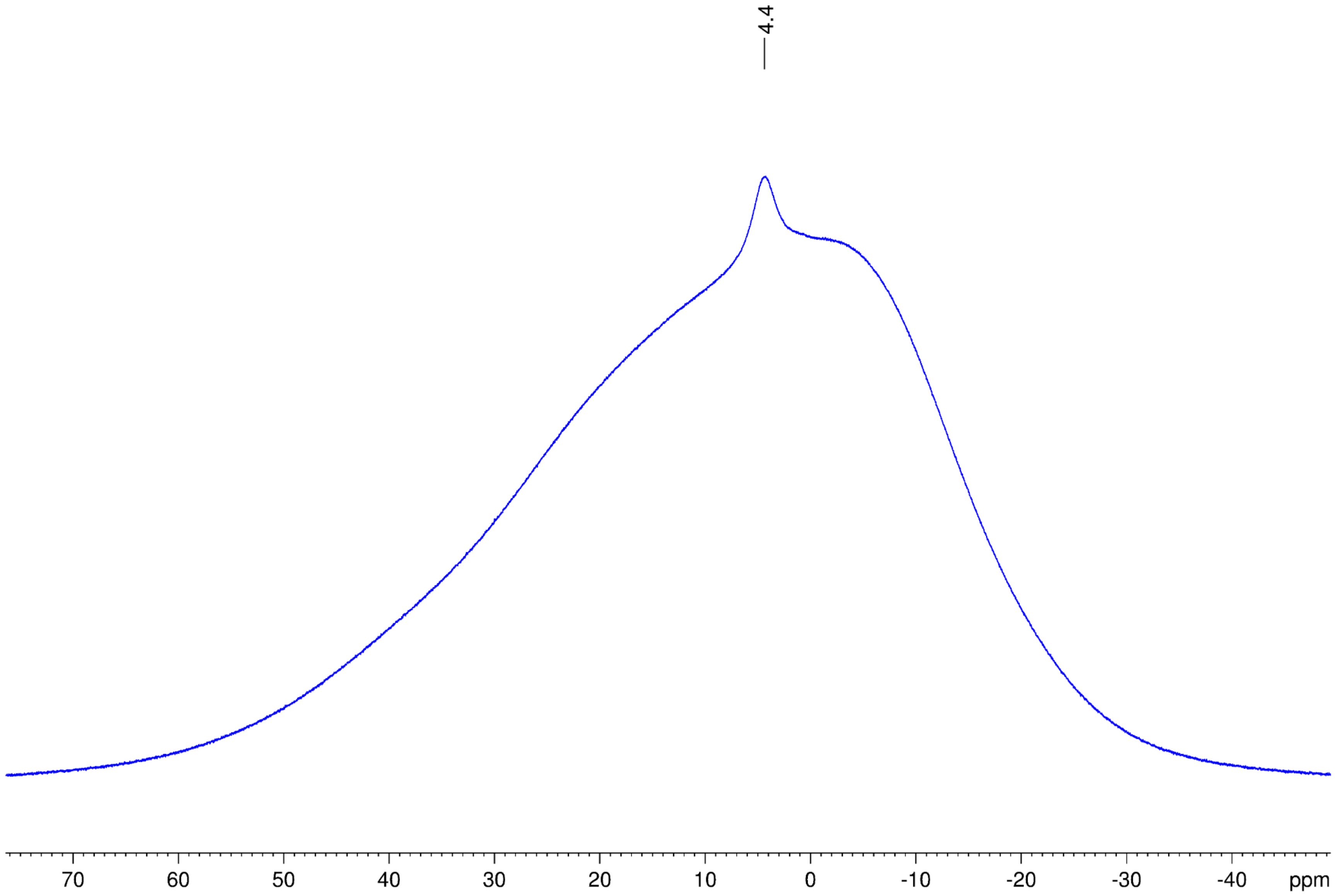

Figure S6. ^{11}B NMR spectrum of **2-Ru** in C_6D_6.

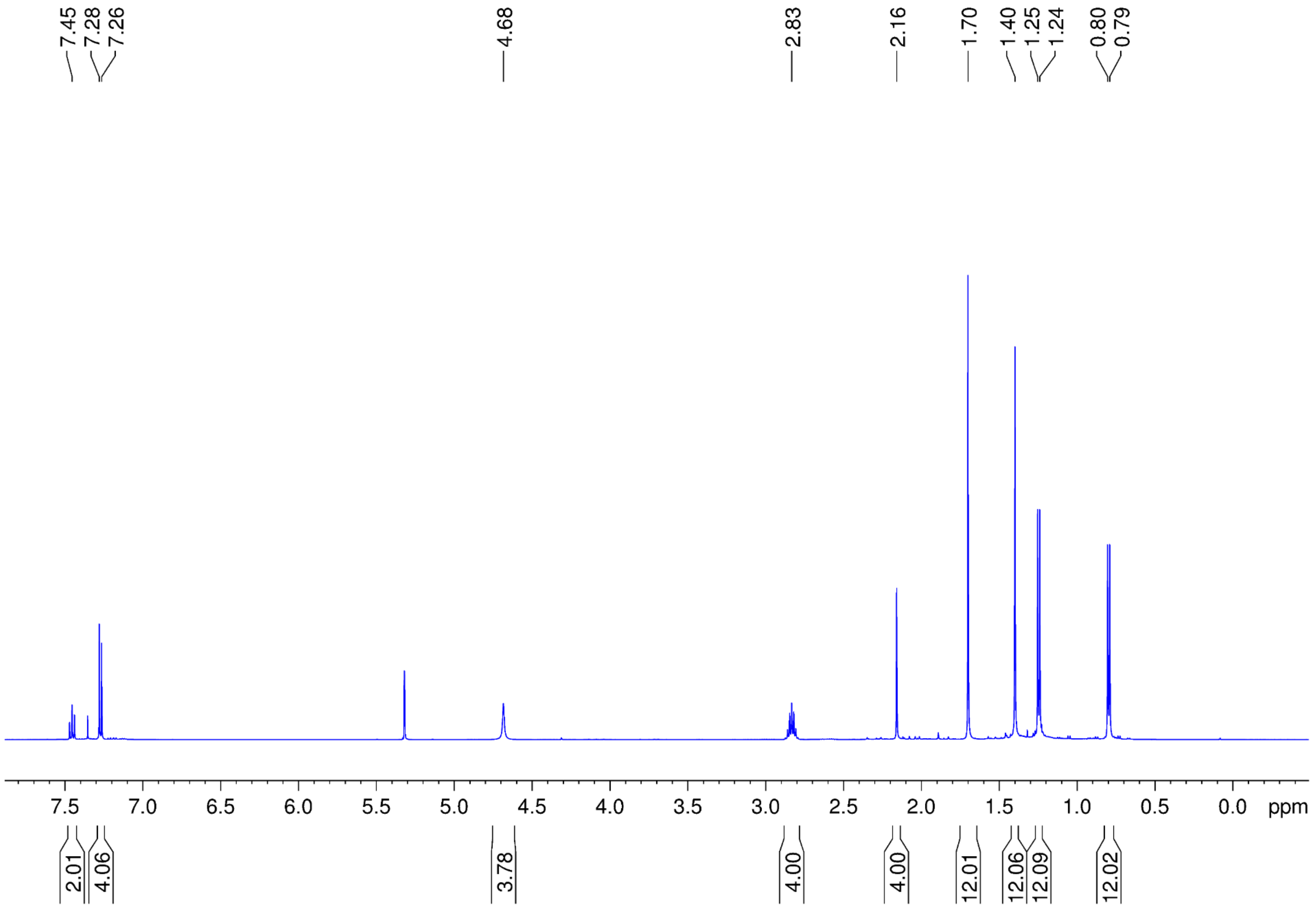

Figure S7. $^{1}H\{^{11}B\}$ NMR spectrum of **3-P** in C_6D_6.

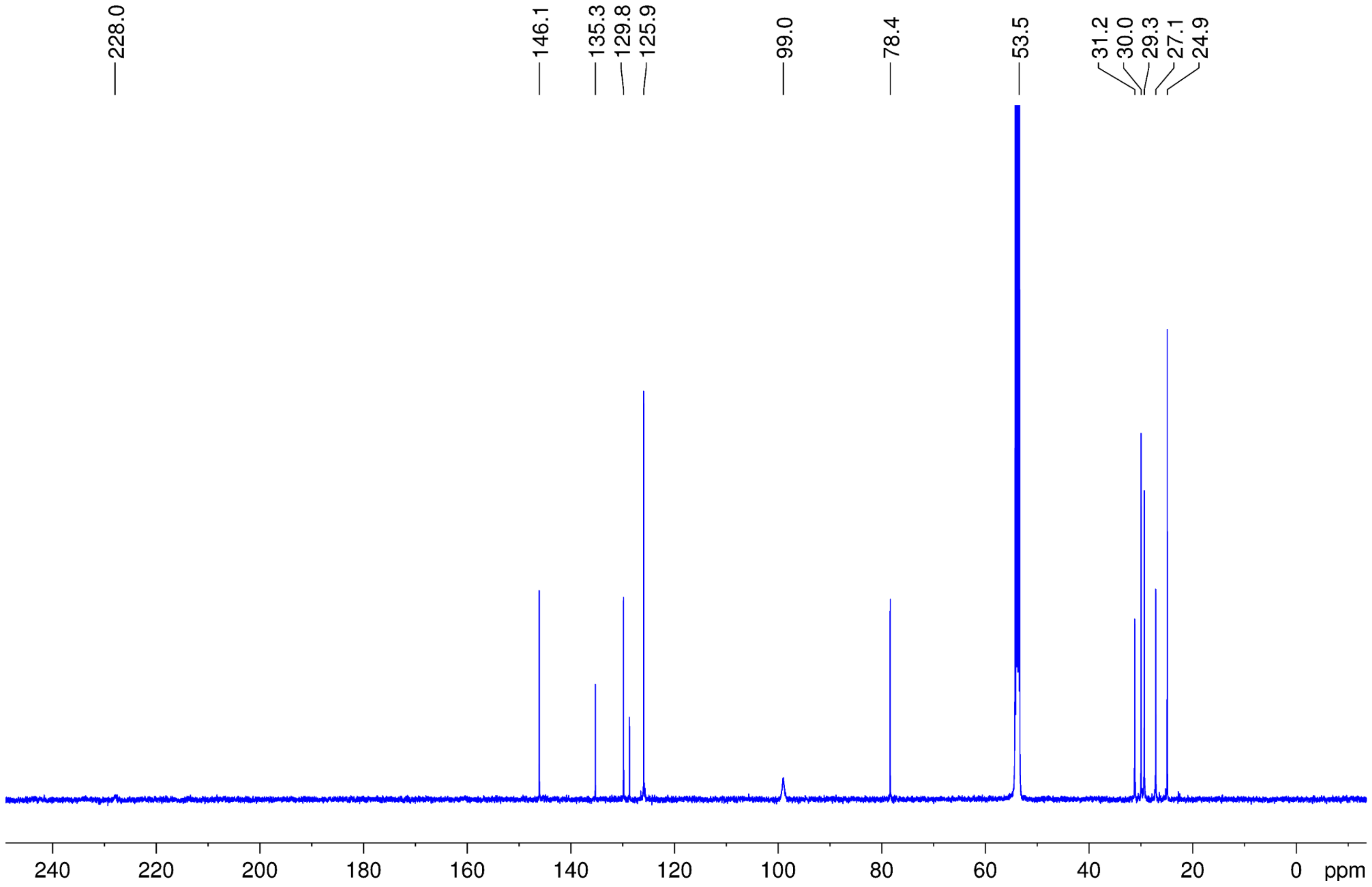

Figure S8. $^{13}C\{^1H\}$ NMR spectrum of **3-P** in C_6D_6.

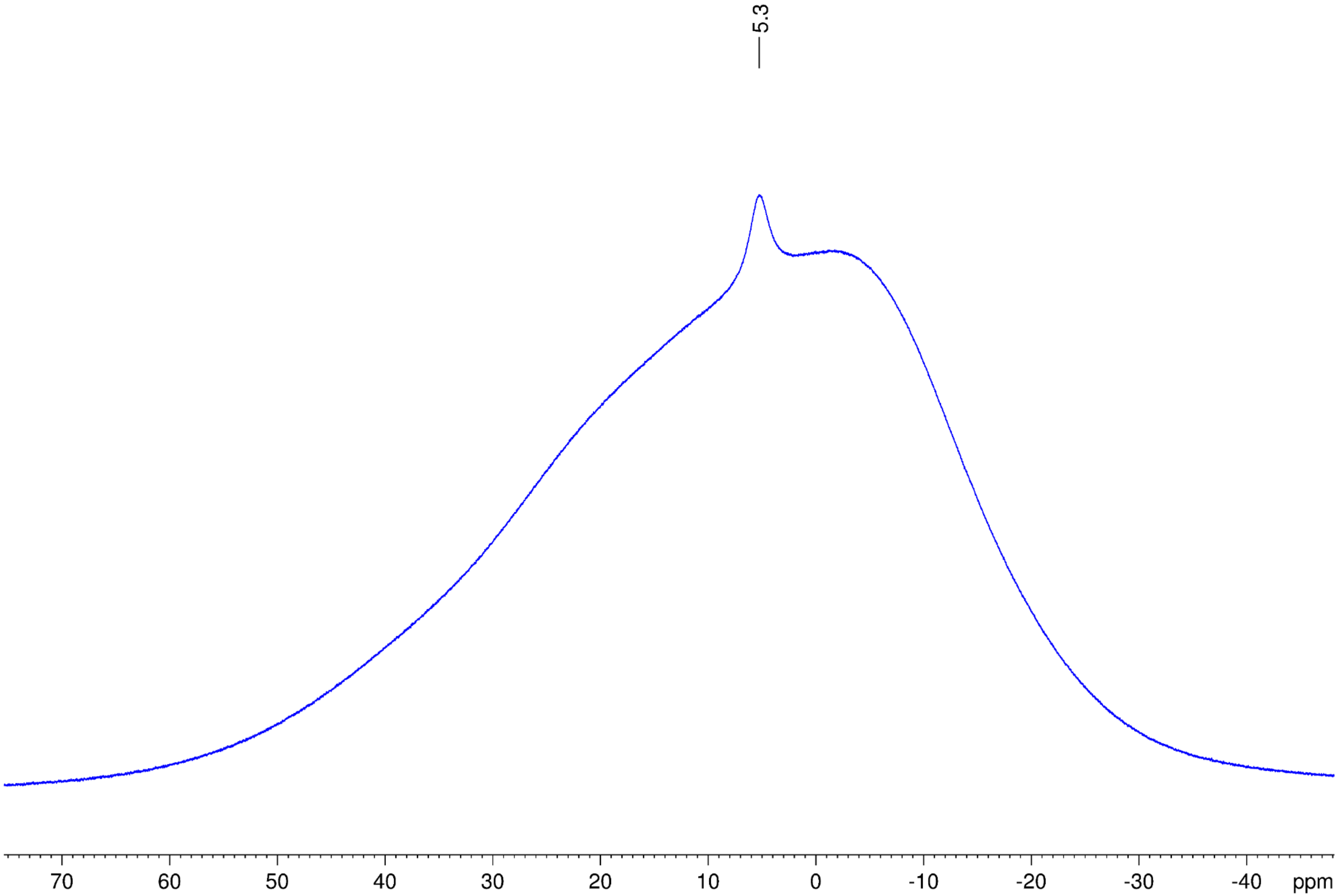

Figure S9. ^{11}B NMR spectrum of **3-P** in C_6D_6.

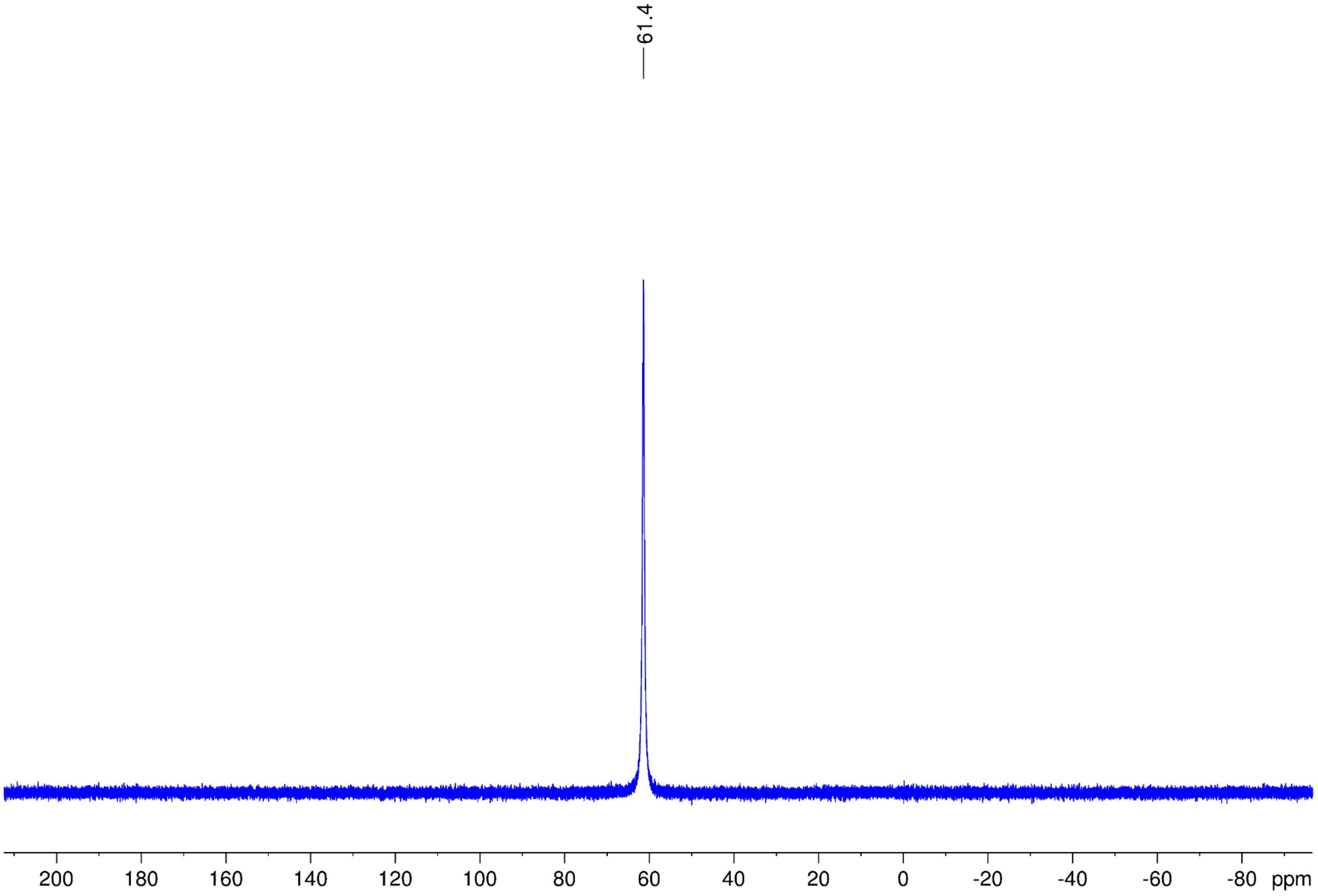

Figure S10. ^{31}P NMR spectrum of **3-P** in C_6D_6.

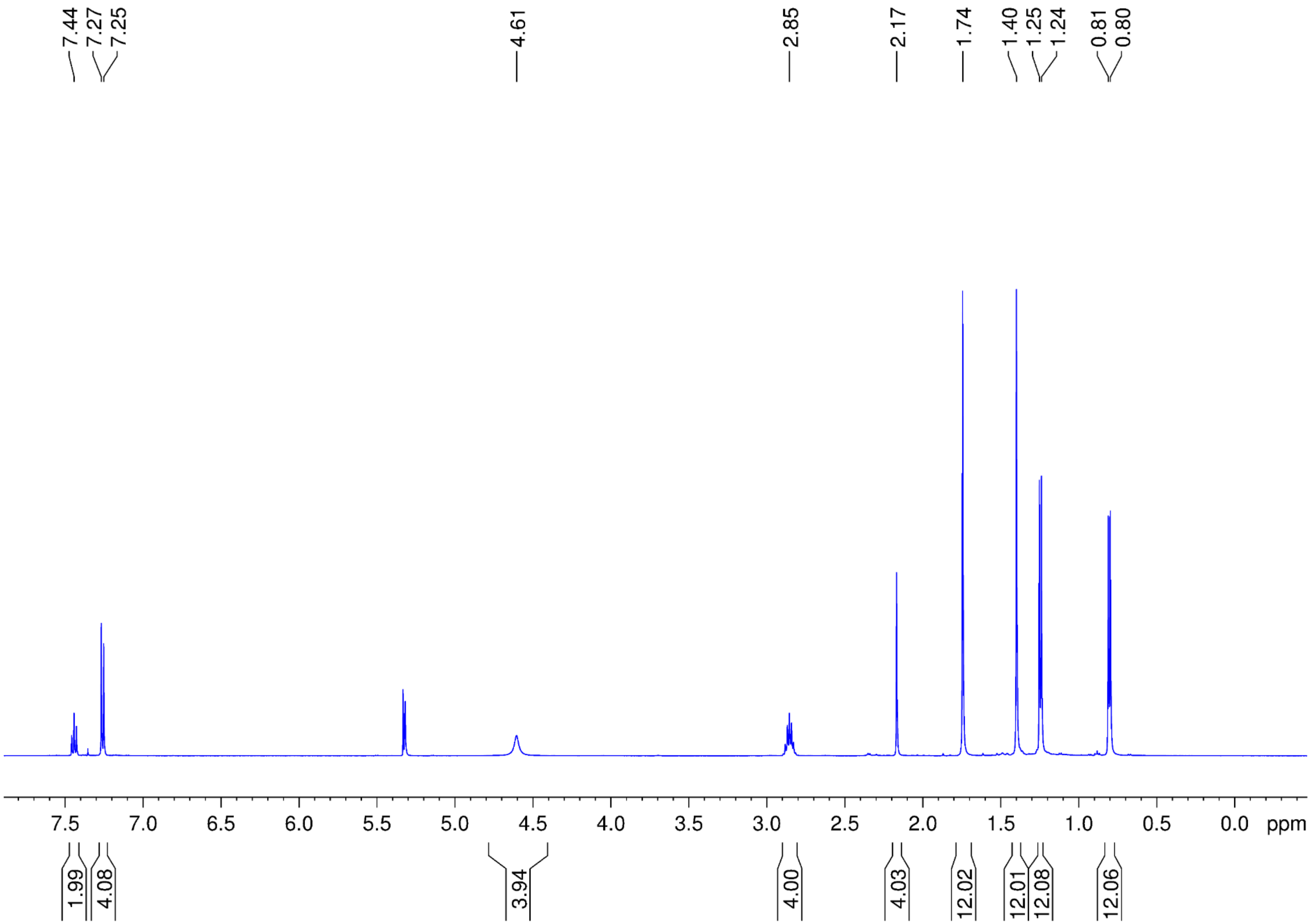

Figure S11. $^{1}H\{^{11}B\}$ NMR spectrum of **3-As** in C_6D_6.

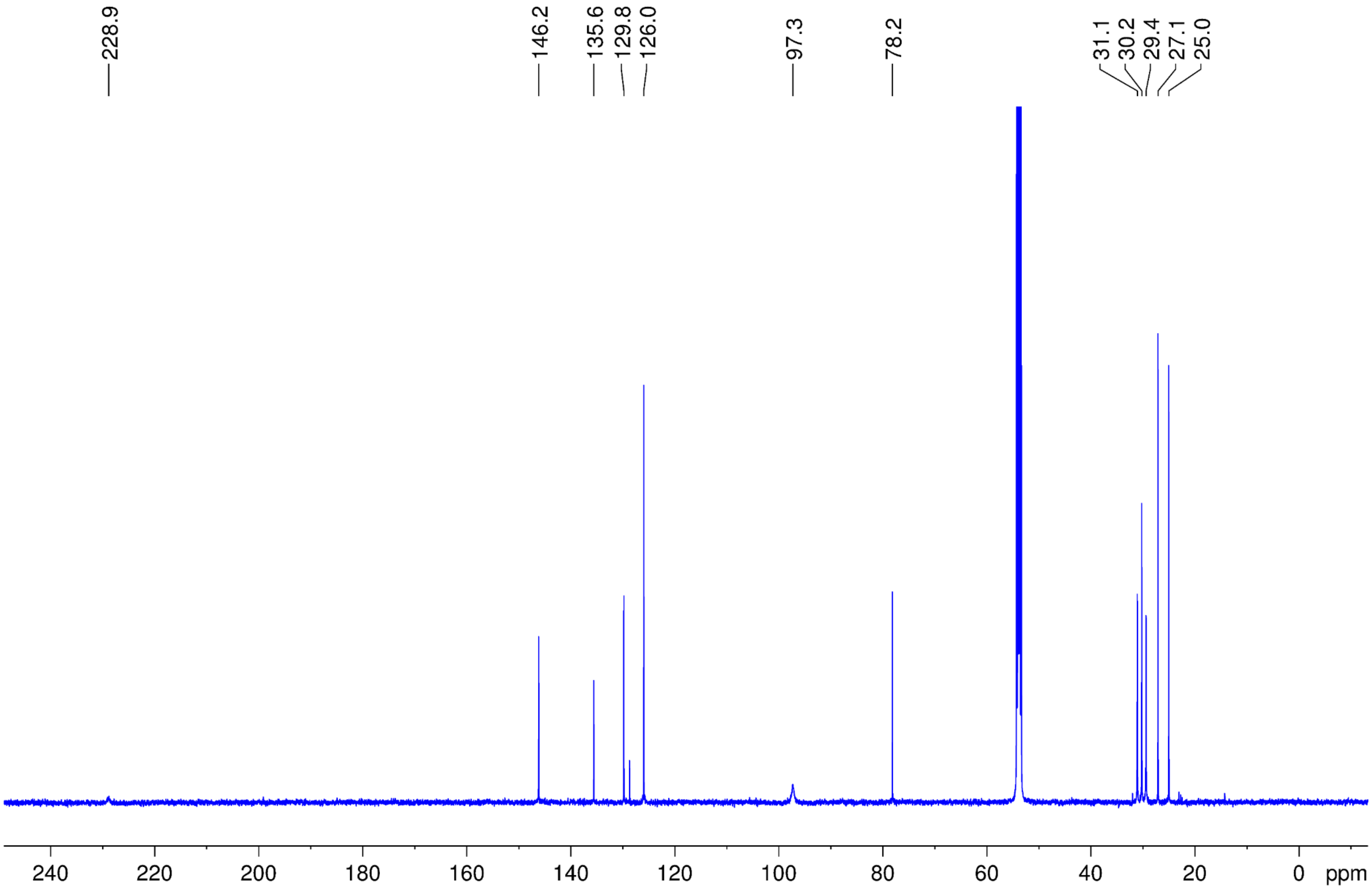

Figure S12. $^{13}C\{^{1}H\}$ NMR spectrum of **3-As** in C_6D_6.

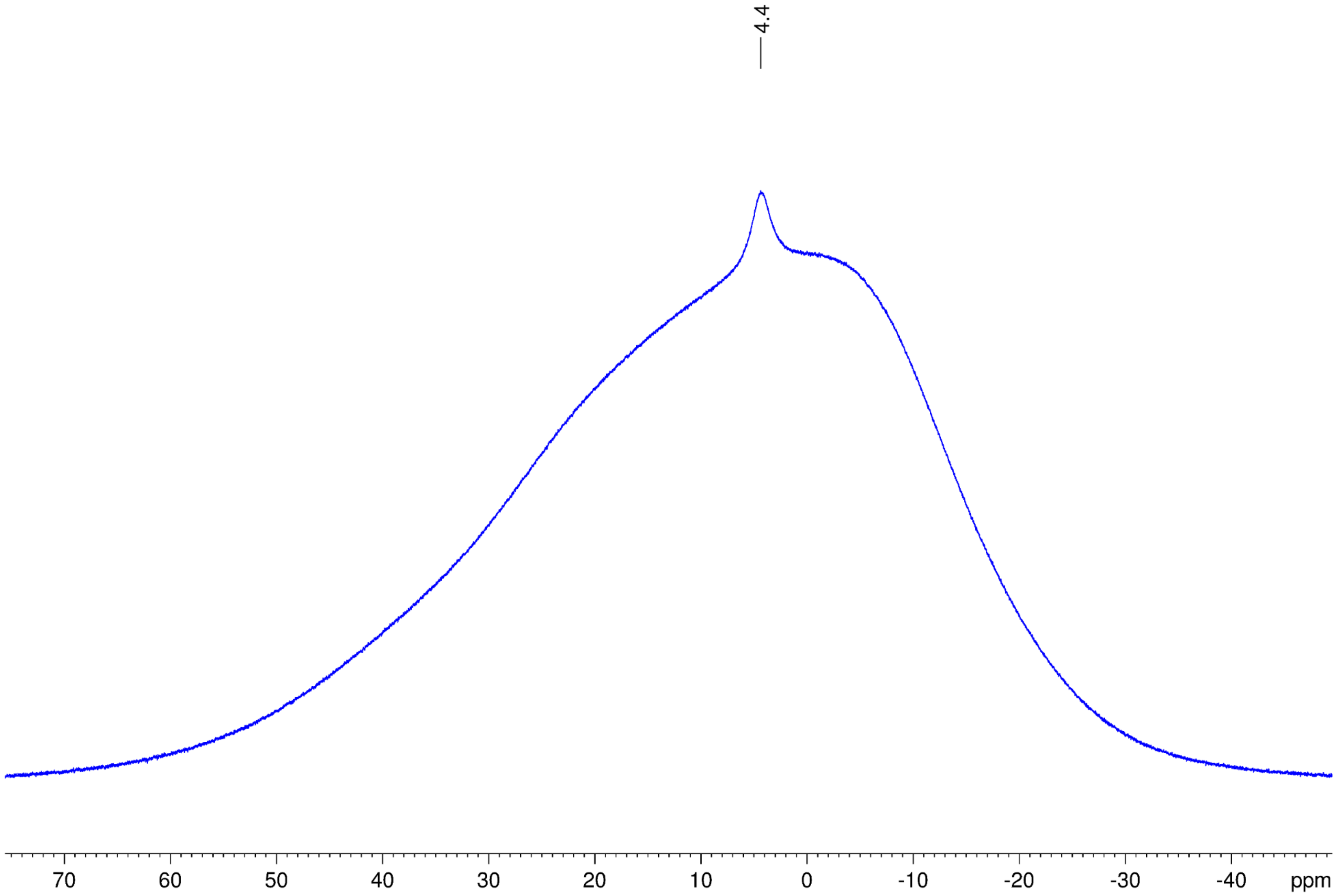

Figure S13. ^{11}B NMR spectrum of **3-As** in C_6D_6.

IR spectra

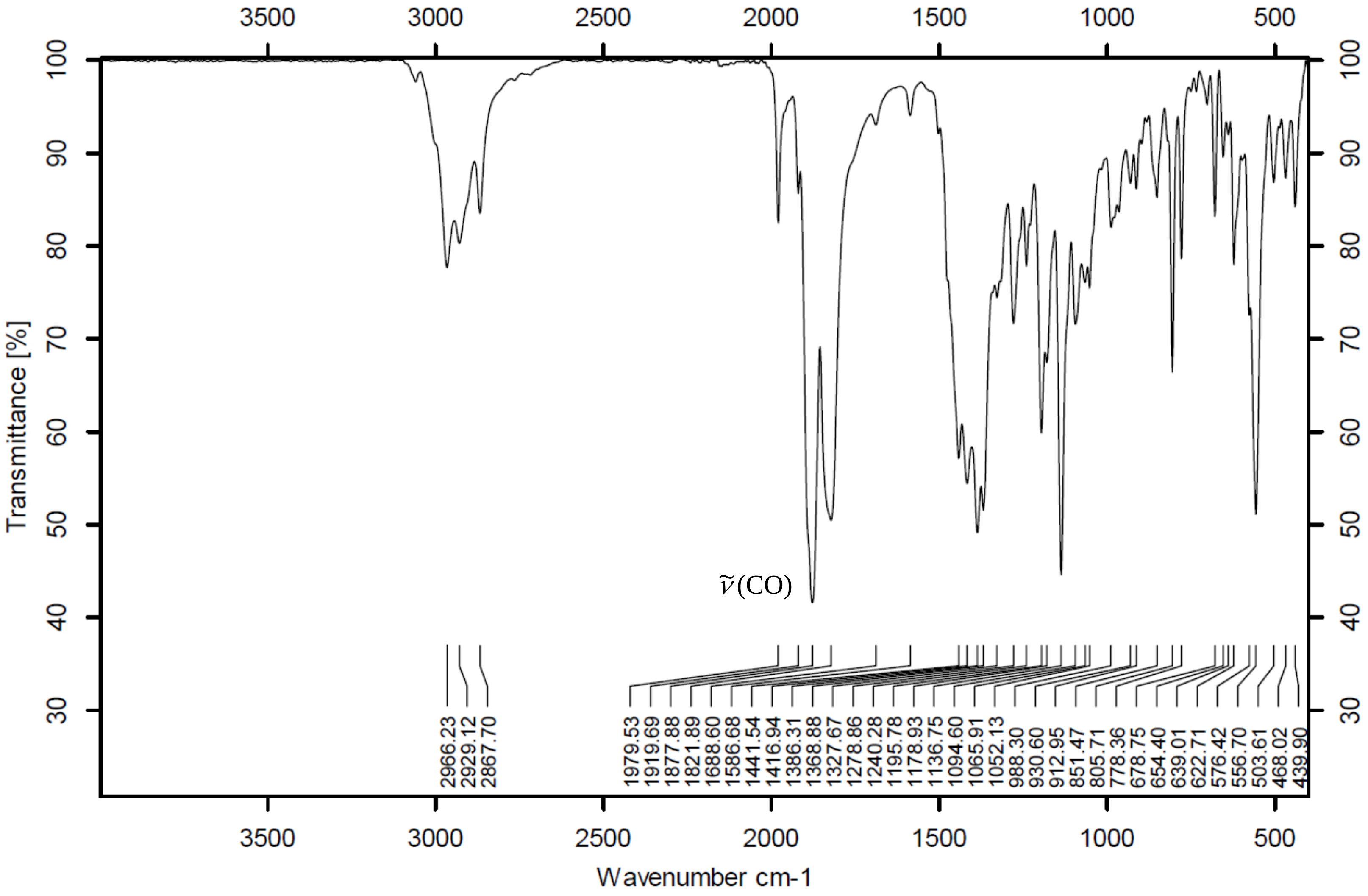

Figure S14. Solid-state IR spectrum of **2-Fe**.

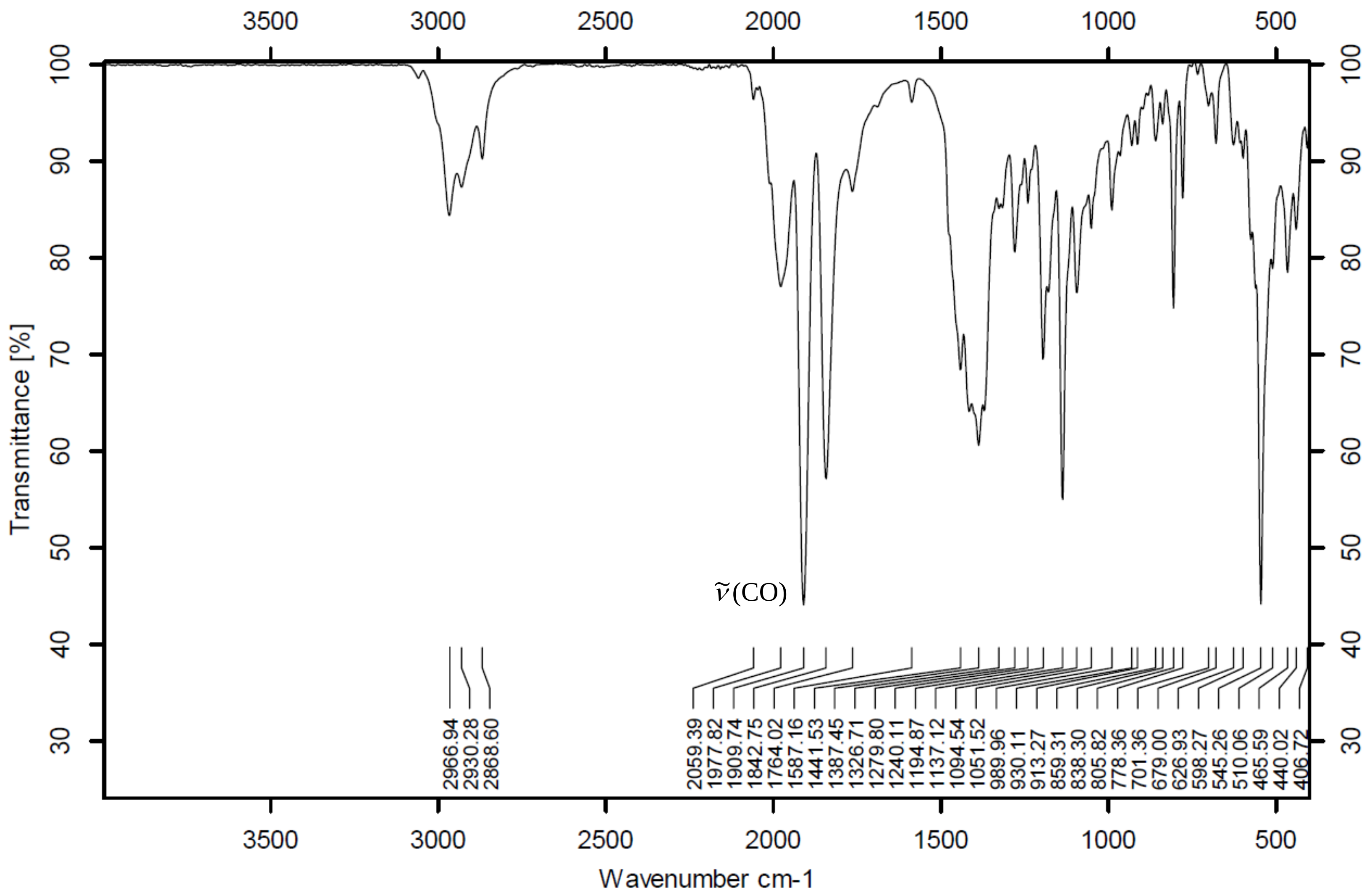

Figure S15. Solid-state IR spectrum of **2-Ru**.

High-resolution mass spectra

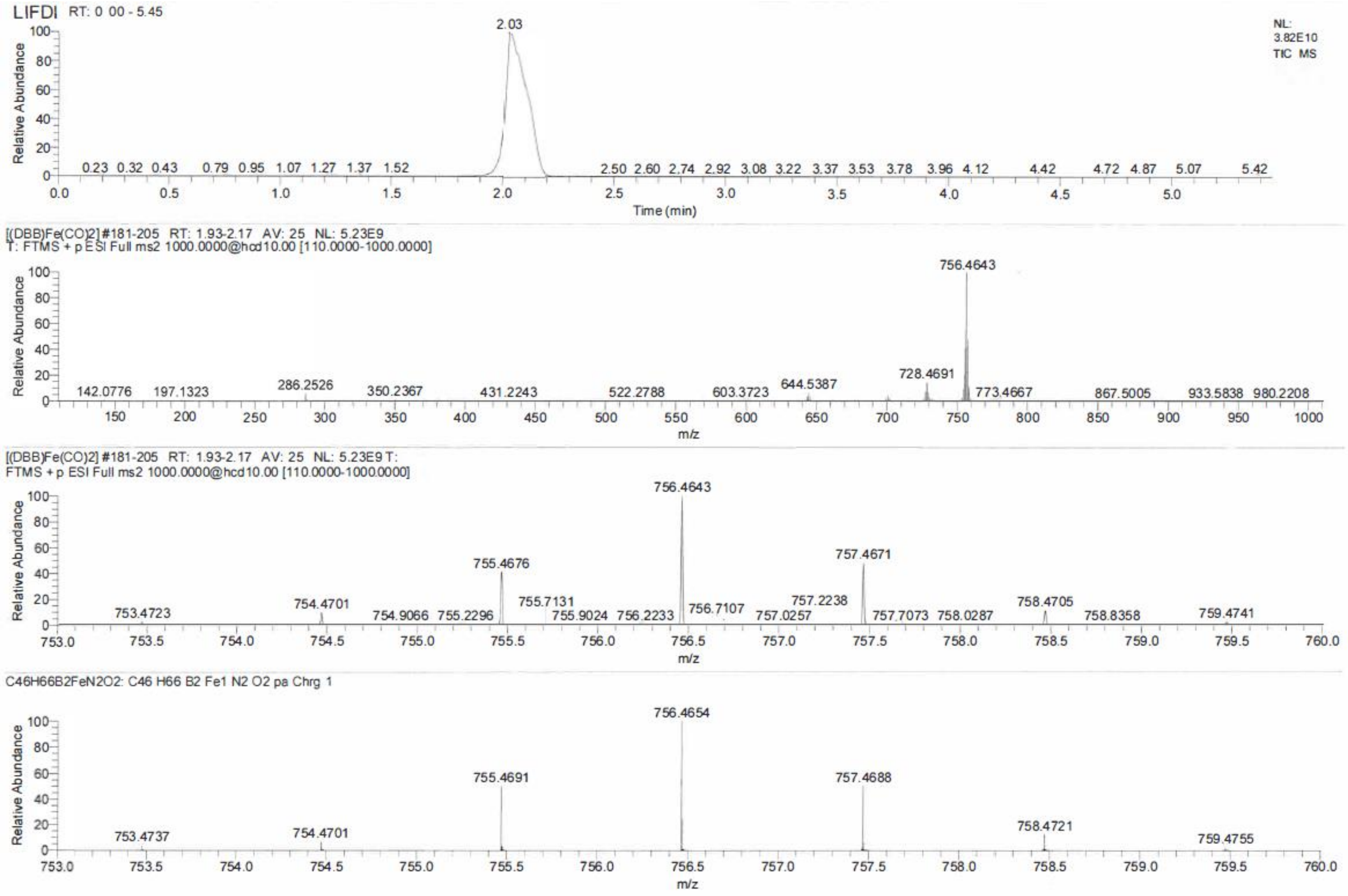

Figure S16. HRMS of **2-Fe**.

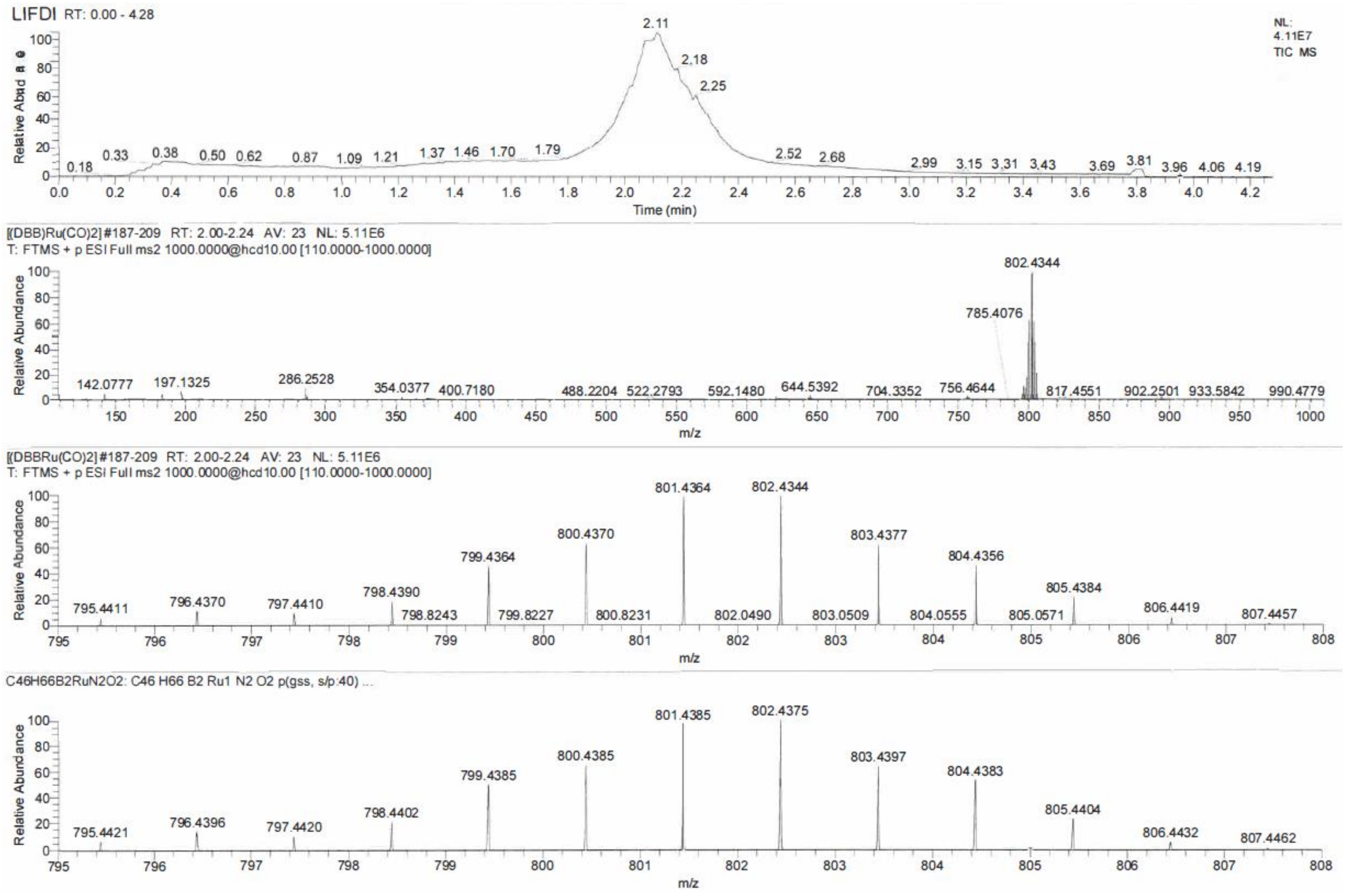

Figure S17. HRMS of **2-Ru**.

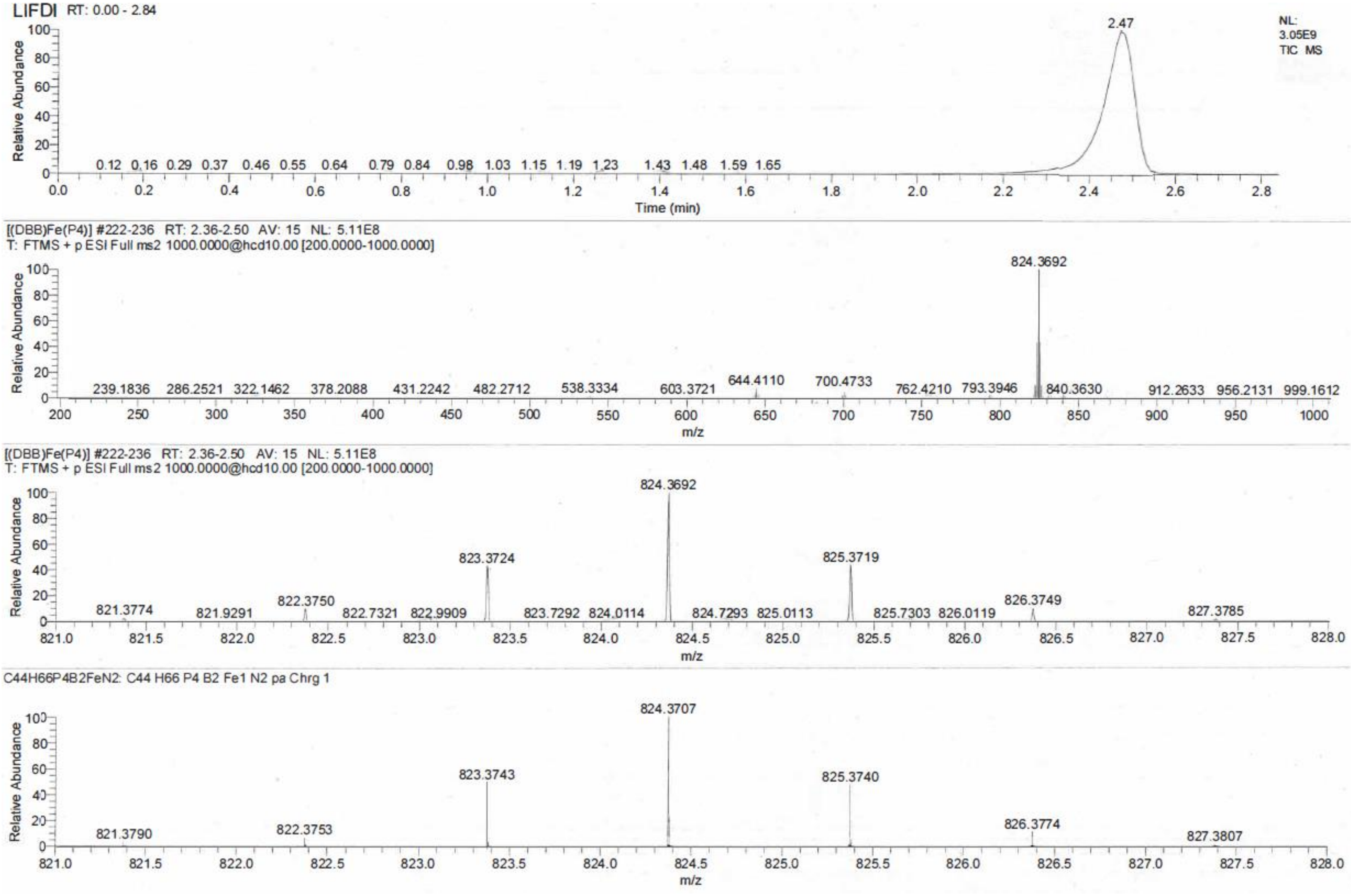

Figure S18. HRMS of **3-P**.

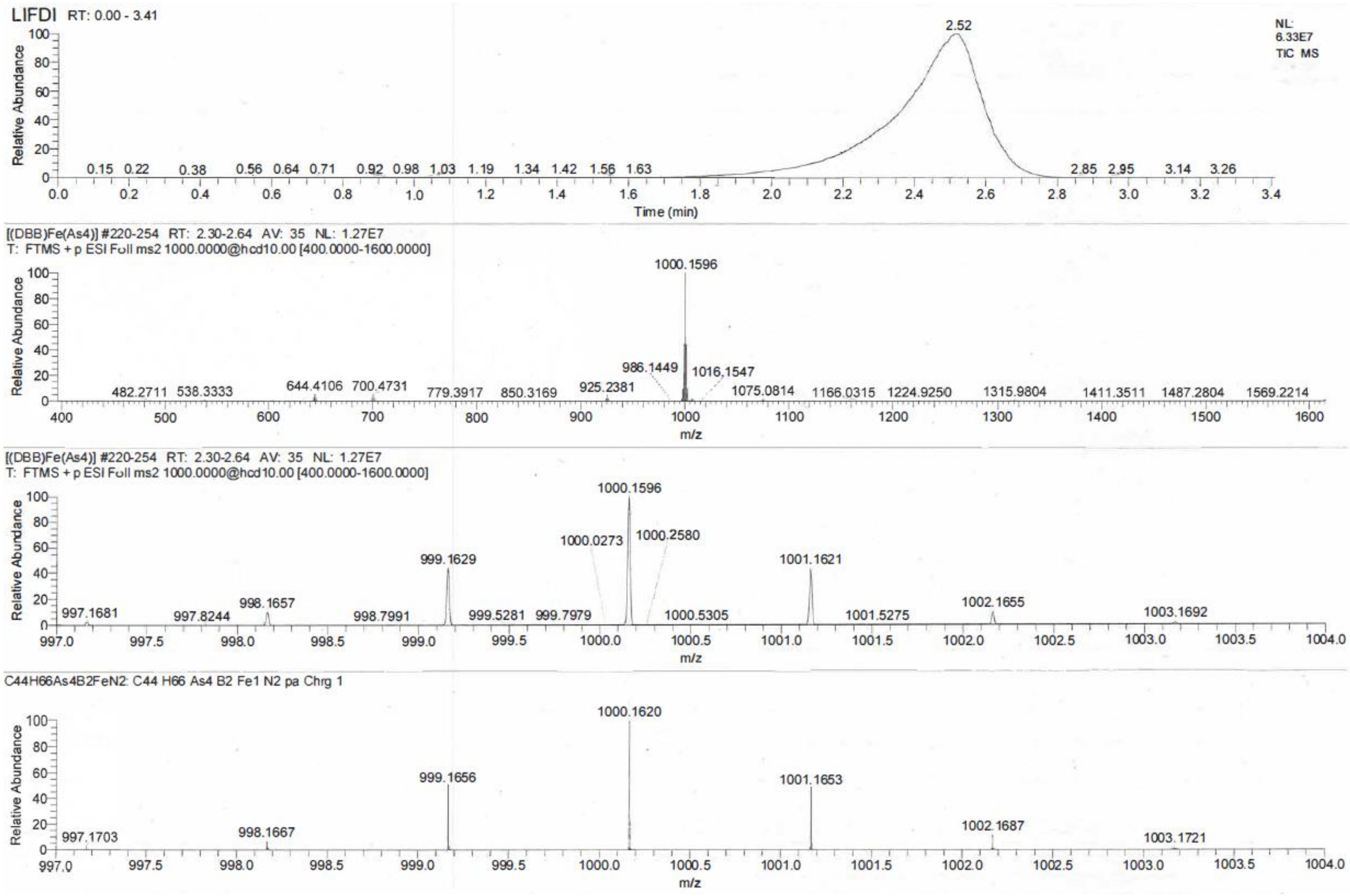

Figure S19. HRMS of **3-As**.

UV-vis spectra

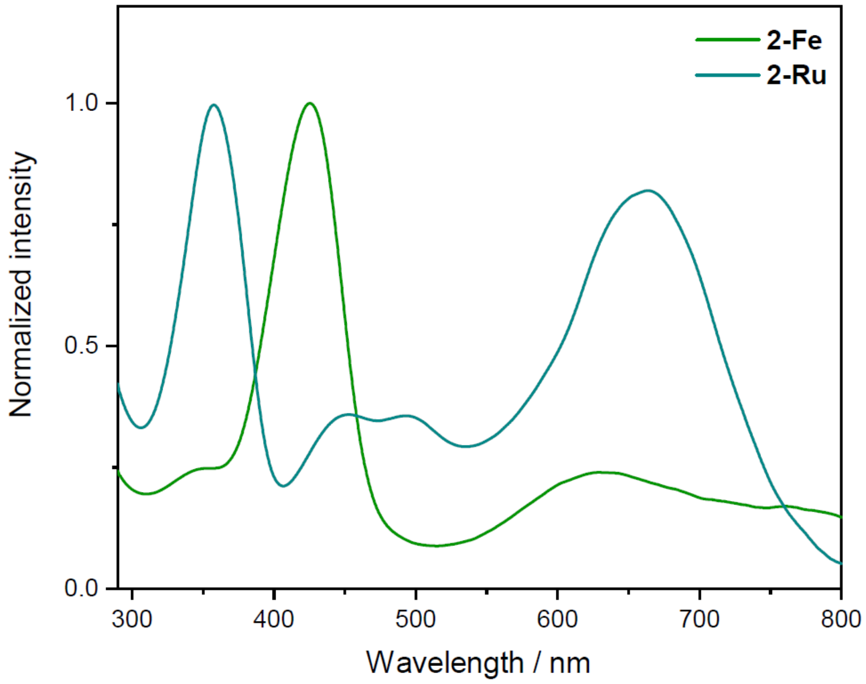

Figure S20. Overlay of UV-vis absorption spectra of **2-Fe** (dark green) and **2-Ru** (dark turquoise) in benzene at 23 °C. **2-Fe**: λ_{max} = 425 nm, λ_2 = 629 nm; **2-Ru**: λ_{max} = 358 nm, λ_2 = 664 nm, λ_3 = 453 and 494 nm.

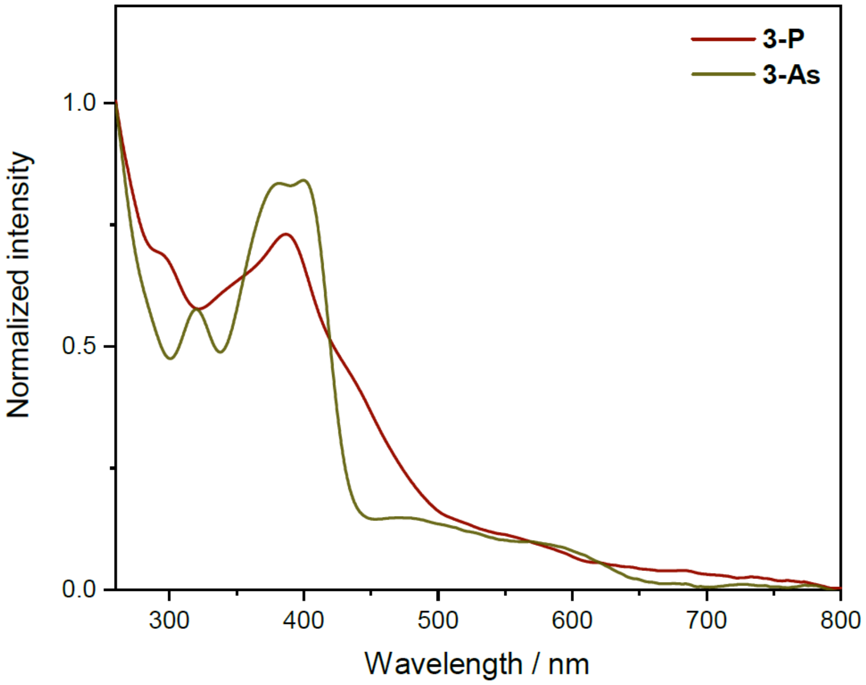

Figure S21. Overlay of UV-vis absorption spectra of **3-P** (red-brown) and **3-As** (green-brown) in benzene at 23 °C. **3-P**: λ_{max} = 387 nm, λ_2 = 435 nm (shoulder), λ_3 = 552 nm; **3-As**: λ_{max} = 382 and 400 nm, λ_2 = 470 nm, λ_3 = 570 nm.

<u>**Cyclic voltammetry**</u>

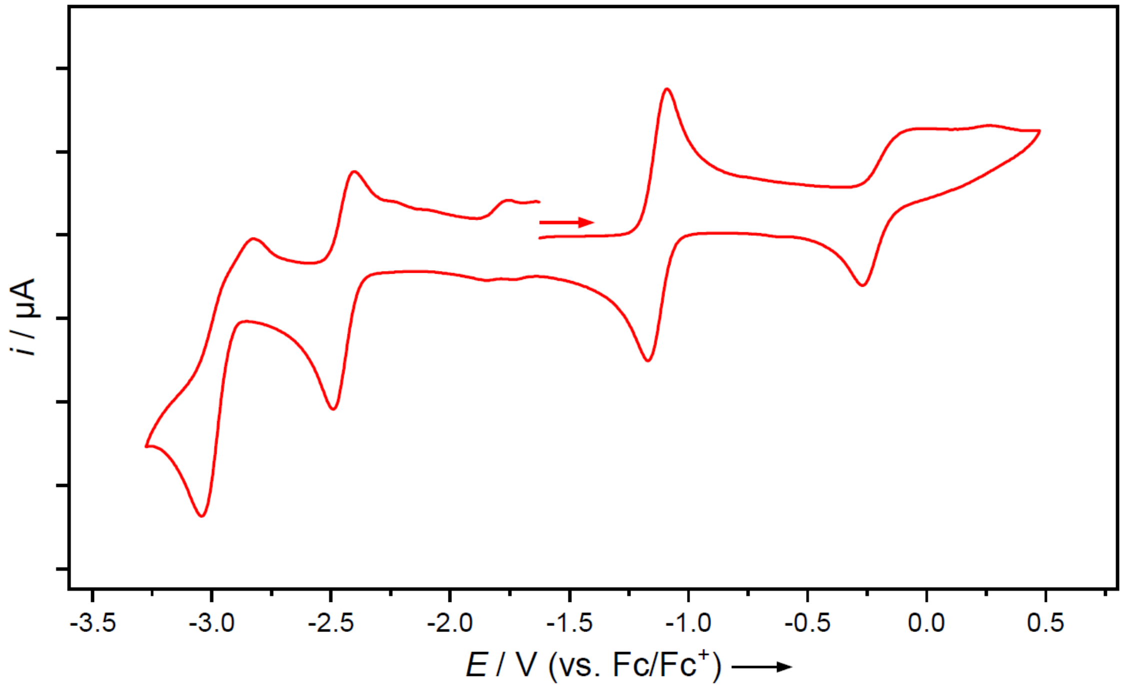

Figure S22. Cyclic voltammogram of **2-Fe** in THF/0.1 M [nBu$_4$N][PF$_6$] measured at 250 mV s^{-1} with voltammetric response. Formal potentials: E_{pc} = −3.05 V, $E_{1/2_1}$ = −2.46 V, $E_{1/2_2}$ = −1.13 V, E_{pa} = ca. −0.02 V.

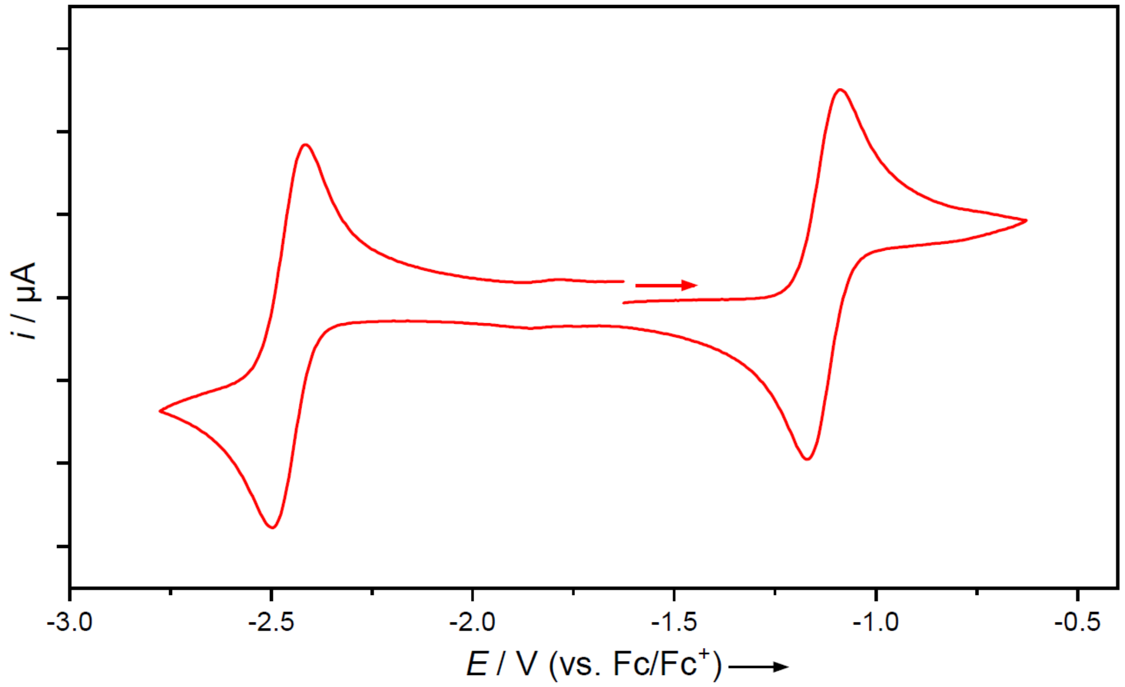

Figure S23. Cyclic voltammogram of **2-Fe** in THF/0.1 M [nBu$_4$N][PF$_6$] measured at 250 mV s^{-1} with voltammetric response. Formal potentials: $E_{1/2_1}$ = −2.46 V, $E_{1/2_2}$ = −1.13 V.

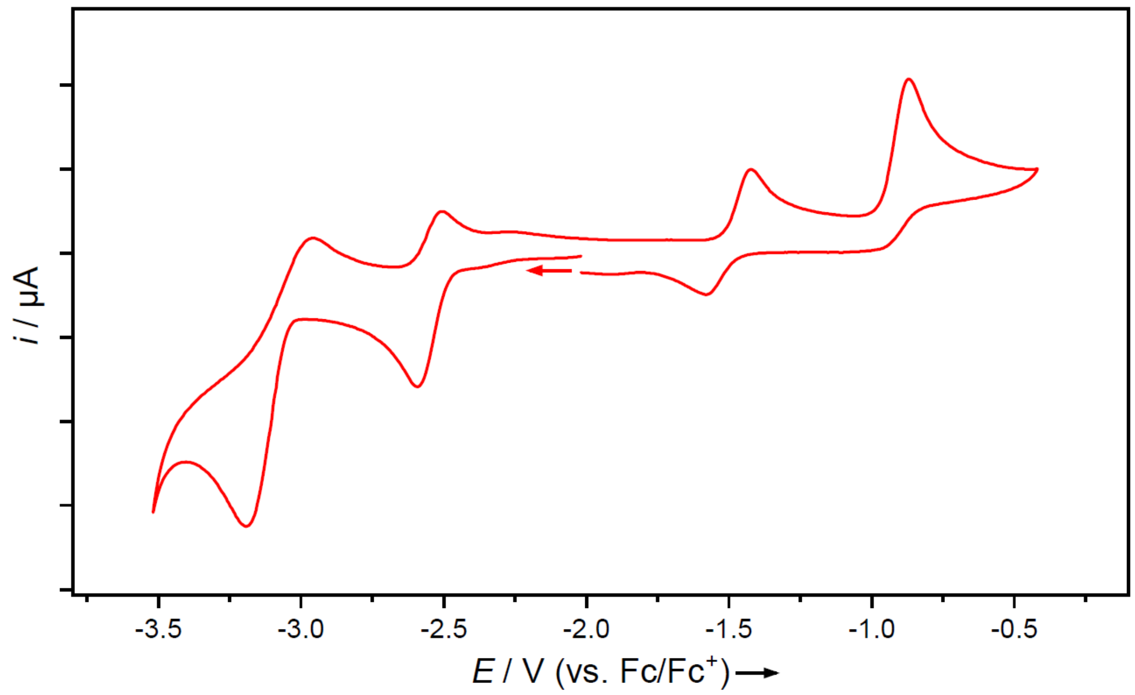

Figure S24. Cyclic voltammogram of **2-Ru** in THF/0.1 M [nBu$_4$N][PF$_6$] measured at 250 mV s^{-1} with voltammetric response. Formal potentials: E_{pc} = −3.19 V, $E_{1/2}$ = −2.53 V, E_{pa1} = −1.42 V, E_{pa2} = −0.87 V.

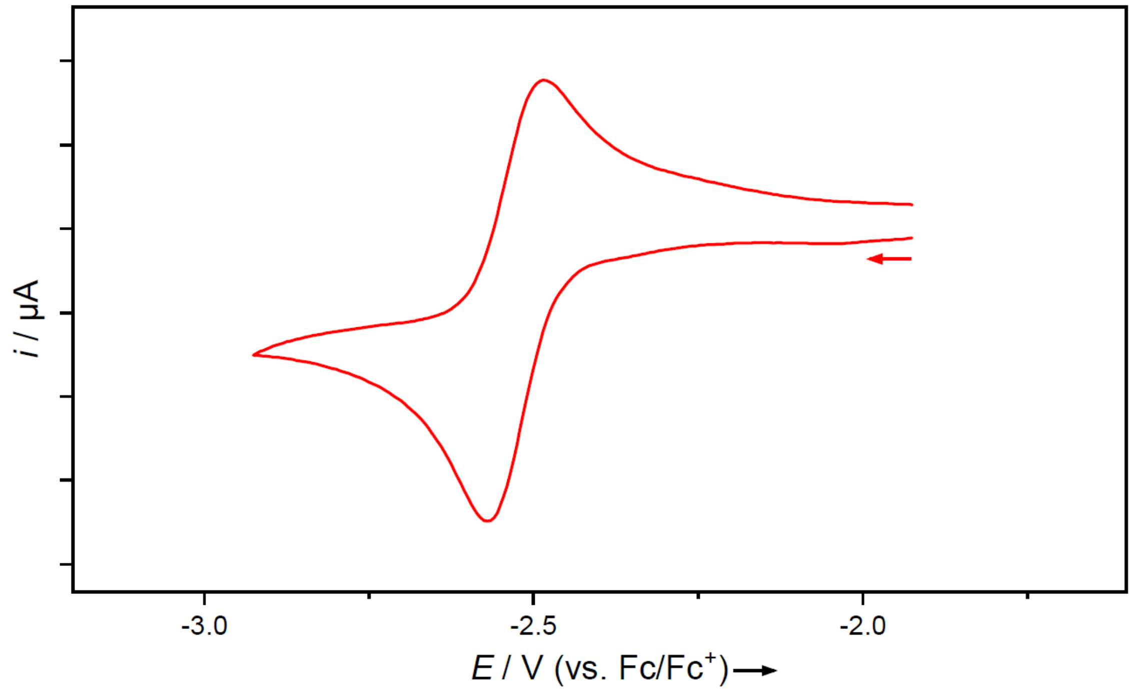

Figure S25. Cyclic voltammogram of **2-Ru** in THF/0.1 M [nBu$_4$N][PF$_6$] measured at 250 mV s^{-1} with voltammetric response. Formal potentials: $E_{1/2}$ = −2.53 V.

X-ray crystallographic data

The crystal data of **2-Fe**, **2-Ru, 3-P** and **3-As** were collected on a *XtaLAB Synergy Dualflex HyPix* diffractometer with a Hybrid Pixel array detector and multi-layer mirror monochromated $Cu_{K\alpha}$ radiation. The structures were solved using the intrinsic phasing method,[3] refined with the ShelXL program[4] and expanded using Fourier techniques. All non-hydrogen atoms were refined anisotropically. Hydrogen atoms were included in structure factor calculations. All hydrogen atoms were assigned to idealized geometric positions.

Crystallographic data have been deposited with the Cambridge Crystallographic Data Center as supplementary publication nos. CCDC 2169216 (**3-As**), 2169217 (**2-Ru**), 2169218 (**2-Fe**) and 2169219 (**3-P**). These data can be obtained free of charge from The Cambridge Crystallographic Data Center *via* www.ccdc.cam.ac.uk/data_request/cif.

Crystal data for 2-Fe: $C_{46}H_{66}B_2FeN_2O_2$, M_r = 756.47, clear black block, 0.217×0.100×0.046 mm^3, orthorhombic space group *Pbca*, a = 16.19730(10) Å, b = 21.45830(10) Å, c = 24.78820(10) Å, $\alpha = \beta = \gamma = 90°$, V = 8615.55(8) Å^3, Z = 8, ρ_{calcd} = 1.166 g·cm^{-3}, μ = 3.084 mm^{-1}, F(000) = 3264, T = 100.00(10) K, R_1 = 0.0380, wR_2 = 0.0872, 8483 independent reflections [$2\theta \leq 144.244°$] and 494 parameters.

Crystal data for 2-Ru: $C_{46}H_{66}B_2N_2O_2Ru{\cdot}C_6H_6$, M_r = 879.80, clear black plate, 0.094×0.069×0.014 mm^3, orthorhombic space group *Pbca*, a = 20.8220(3) Å, b = 18.9766(2) Å, c = 24.4586(3) Å, $\alpha = \beta = \gamma = 90°$, V = 9664.3(2) Å^3, Z = 8, ρ_{calcd} = 1.209 g·cm^{-3}, μ = 2.919 mm^{-1}, F(000) = 3744, T = 100.00(11) K, R_1 = 0.0572, wR_2 = 0.0779, 9166 independent reflections [$2\theta \leq 140.15°$] and 548 parameters.

Refinement details for 3-P: Refined as a two-component twin. Component 2 rotated by –179.9444° around [0.00 1.00 –0.00] (reciprocal) or [0.26 0.95 0.16] (direct) The BASF parameter was refined to 39.6%. The asymmetric unit contains 1.5 molecules of benzene, the first fully occupied and modelled as twofold rotationally disordered (RESI 6 and 16 BENZ) in

a 60:40 ratio, the second half occupied on an inversion centre and modelled as twofold rotationally disordered (RESI 7 and 17 BENZ) in a 69:39 ratio. All benzene rings within the disorders were idealized using AFIX 66 and ADPs restrained using SIMU and ISOR 0.01. The Dipp substituent of one CAAC ligand (RESI 51 and 51 Dipp) was modelled as twofold rotationally disordered in a 65:35 ratio. The aryl ring within this disorder was idealized with AFIX 66 and ADPs restrained with SIMU 0.01.

Crystal data for 3-P: $C_{44}H_{66}B_2FeN_2P_4{\cdot}(C_6H_6)_{1.5}$, M_r = 941.50, clear yellow plate, 0.195×0.131×0.039 mm^3, triclinic space group $P\bar{1}$, a = 9.9240(2) Å, b = 16.5160(5) Å, c = 16.8008(3) Å, α = 96.857(2)°, β = 106.093(2)°, γ = 96.836(2)°, V = 2593.28(11) Å^3, Z = 2, ρ_{calcd} = 1.206 g·cm^{-3}, μ = 3.761 mm^{-1}, F(000) = 1006, T = 100.00(12) K, R_1 = 0.1030, wR_2 = 0.2653, 15643 independent reflections [$2\theta \leq 136.498°$] and 754 parameters.

Refinement details for 3-As: Collected reflections were indexed and integrated as a single crystal. The data were later refined as a two-component twin using Platon TwinRotMat.[5] Component 2 rotated by 4.60° around (0 0 1) (reciprocal) or [0 –1 5] (direct). The BASF parameter was refined to 46.5%. The asymmetric unit contains three highly disordered CH_2Cl_2 molecules, which could not be modelled satisfactorily and have therefore been treated as a diffuse contribution to the overall scattering without specific atom positions by SQUEEZE/PLATON.[6] 250 electrons were thus squeezed from the entire unit cell, i.e. 5.95 (ca. 6) molecules of CH_2Cl_2.

Crystal data for 3-As: $C_{44}H_{66}As_4B_2FeN_2{\cdot}[(CH_2Cl_2)_3$ squeezed], M_r = 1254.91, clear light yellow plate, 0.419×0.157×0.023 mm^3, triclinic space group $P\bar{1}$, a = 10.0843(3) Å, b = 16.3614(4) Å, c = 16.9614(5) Å, α = 81.185(2)°, β = 83.687(2)°, γ = 73.224(2)°, V = 2641.37(13) Å^3, Z = 2, ρ_{calcd} = 1.258 g·cm^{-3}, μ = 5.269 mm^{-1}, F(000) = 1024, T = 100.00(10) K, R_1 = 0.0805, wR_2 = 0.2142, 9630 independent reflections [$2\theta \leq 136.478°$] and 495 parameters.

Computational details

All electronic structure calculations were performed using the Gaussian16 rev.C.01 package.[7] Geometry optimizations were carried out in the gas phase with the long-range hybrid functional ωB97X-D[8] in conjunction with Weigend and Ahlrichs' def2-sv(p) split valence basis set (called def2-svpp in this paper), which contains a polarizing *d* function for *p* elements and a diffuse *p* set for *d* elements, but no polarization functions for hydrogen atoms.[9] Several oxidation states and multiplicities were tested for the metal centers. Bond strengths and dissociation energies were calculated with corrections for the basis set superposition error (BSSE) using Boys and Bernardi's counterpoise method,[10-12] as implemented in Gaussian, according to the following equation:

$$\Delta E = \Delta E_{el} + ZPE(AB) - ZPE(A) - ZPE(B) - \delta^{BSSE} \tag{1}$$

where $\Delta E_{el} = E_{AB}^{AB}(AB) - E_{A}^{A}(A) - E_{B}^{B}(B)$. Here $E_{Z}^{Y}(X)$ represents the energy of subsystem *X* at the optimized geometry *Y* using the basis set *Z*. The counterpoise correction is defined as:[12]

$$\delta^{BSSE} = E_{AB}^{A}(A) + E_{AB}^{B}(B) - E_{AB}^{AB}(A) - E_{AB}^{AB}(B) \tag{2}$$

To assess aromaticity nucleus-independent chemical shifts (NICS)[13-15] and multi-center index (MCI)[16] calculations at the level of theory B3LYP/6-311+G*[17] were performed. Multiwfn version 3.7[18] was used to calculate the MCIs. To investigate the bonding and the atomic charges of the metal complexes the Intrinsic Bonding Orbital (IBO, v20211019)[19,20] and Natural Bonding Orbitals (NBO, version 7.0.6)[21] approaches were employed. Finally, the effective oxidation states (EOS) were calculated using the same Iboview software. The redox charge analysis was done by defining the fragmentation of the system via Topological Fuzzy Voronoi Cells (TFVC) with the default integration grid.[22] For comparative purposes some calculations were also performed on a simplified version of a literature-known end-deck *cyclo*-P_4 iron complex, $[\{\kappa^3\text{-}(PhP(CH_2CH_2PMe_2)_2)\}Fe(\eta^4\text{-}P_4)]$ (**4**),[23] and the model arene complex $[(\eta^6\text{-}C_6Me_6)Fe(\eta^4\text{-}P_4)]$ (**5**).

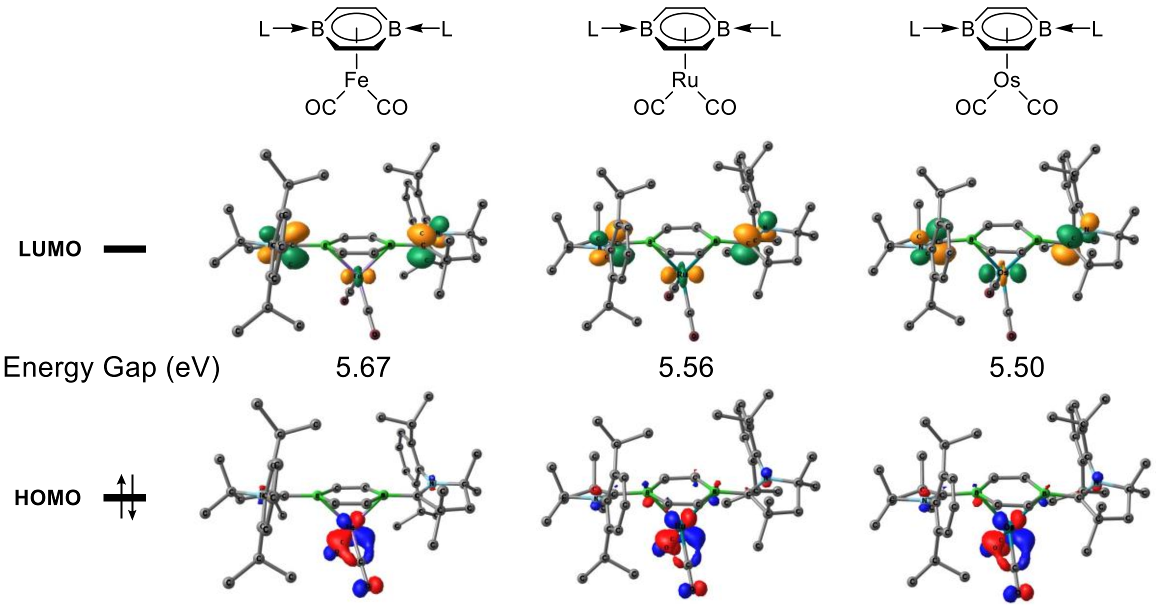

Figure S26. Frontier molecular orbitals of the two-legged parent piano-stool group 8 diborabenzene **2-M** (M = Fe, Ru, Os) complexes calculated at the ωB97X-D/def2-svpp level of theory.

Table S1. Test of different fragmentation possibilities between the diborabenzene (DBB) ligand and the $M(CO)_2$ (M = Fe, Ru, Os) fragment in complexes **2-M**. Each $M(CO)_2$ fragment was calculated with different multiplicities to determine its ground state.

**Complex**	**BDE (kcal mol^{-1})^{a}**
DBB - $Fe(CO)_2$	71.0
DBB - $Ru(CO)_2$	97.9
DBB - $Os(CO)_2$	109.9
DBB^{2-} - $Fe^{2+}(CO)_2$	595.4
DBB^{2-} - $Ru^{2+}(CO)_2$	667.1
DBB^{2-} - $Os^{2+}(CO)_2$	683.4

[a]values contain the BSSE correction.

Table S2. Analysis of $M(CO)_2$ fragments according to our DFT calculations.

Fragment	Stretching frequency (cm^{-1})	Bond distance M–CO (Å)	π backdonation (e^-)
$Fe(CO)_2$	2064	1.70	0.48
$Ru(CO)_2$	2084	1.80	0.14
$Os(CO)_2$	2086	1.81	0.11

Table S1 shows that the fragmentation energies of ionic species $(DBB)^{2-}/[M]^{2+}$ for M = Fe, Ru, Os) are much higher than for neutral moieties. In $[(DBB)M^0(CO)_2]$ complexes fragmentation energies increase from M = Fe to Os, which means that the $M(CO)_2$ fragment becomes less stable down the group. This is also reflected in the M–CO stretching frequencies, which are blue-shifted from M = Fe to Os. M–CO bond distances also increase down the group, while the calculated π backdonation decreases in the same order (see Table S2). This explains why the $Os(CO)_2$ fragment is destabilized compared to its lighter analogues, as previous studies have pointed out for other transition metals and heavier alkaline earth metals.[24]

NICS scans

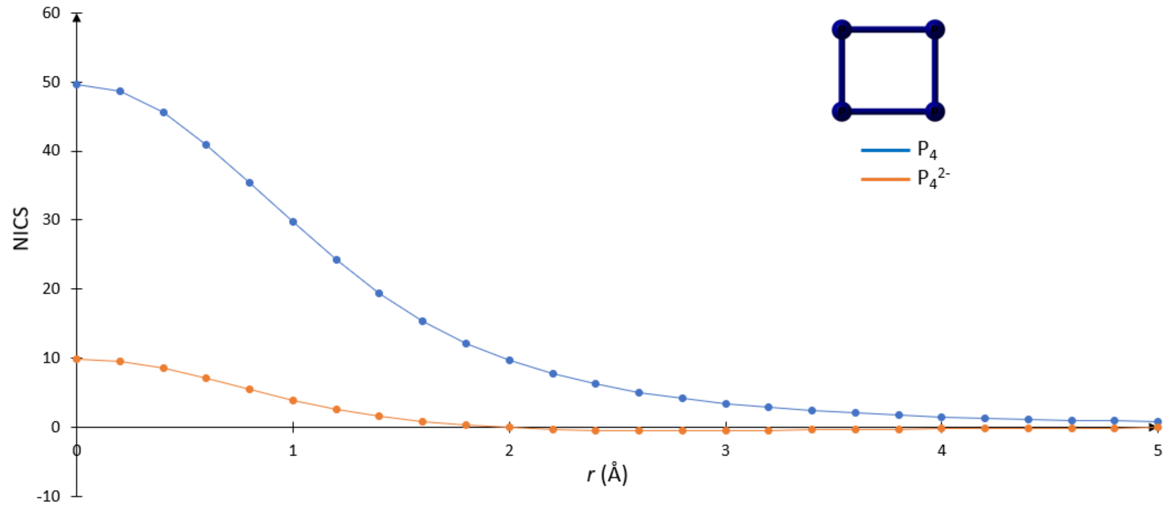

Figure S27. NICS scan for neutral P_4 and dianionic $P_4^{2–}$.

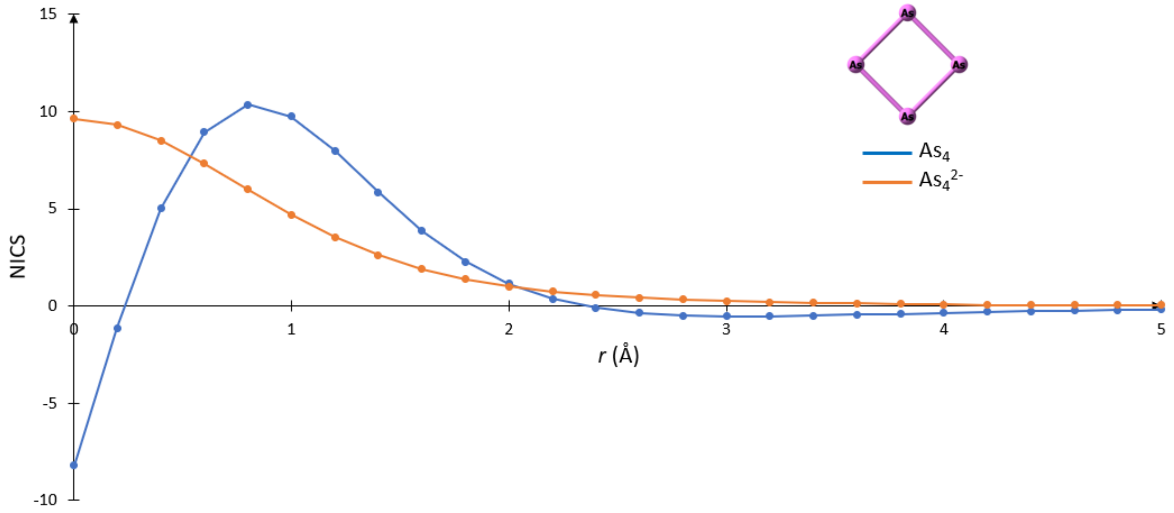

Figure S28. NICS scan for neutral As_4 and dianionic $As_4^{2–}$.

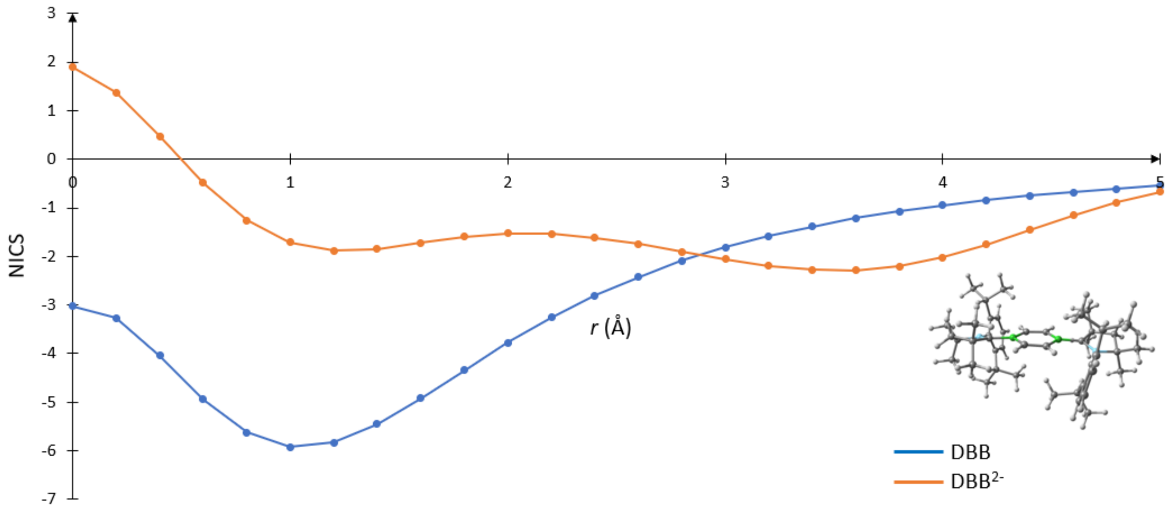

Figure S29. NICS scan for neutral DBB and dianionic DBB^{2-}.

To assess the aromaticity of our cyclic systems before coordination to the transition metals, which is important to rationalize any charge transfer process suggesting an oxidation state change, we performed nucleus-independent chemical shifts (NICS)[25] through the gauge-independent atomic orbital (GIAO) method[26] as implemented in Gaussian. Our calculated NICS scan profiles[27] show the P_4 and As_4 rings are antiaromatic whereas the neutral diborabenzene ring is aromatic. Therefore, the coordination of DBB to the metal center does not result in oxidation to DBB^{2-}, as this would decrease its aromaticity (Figure S29). However, the effect is the opposite for P_4^{2-} and As_4^{2-} species coordinated to the [Fe^{2+}] fragment.
Because NICS is a magnetic aromaticity index, it fails when describing the aromaticity of cyclic systems coordinated to metal centers, as the magnetic field of the metal center directly affects the magnetic field of the tested ring (apparently increases its aromaticity).[28] It is therefore important to measure the electron delocalization, which produces an opposite effect if we consider that the transition metal interacts strongly (donation/backdonation) with the (anti)aromatic ring, using an electronic aromaticity index. The multi-center index (MCI)[29] and its variants have been demonstrated to be robust and reliable aromaticity indices in ligand-TM complexes.[30] Table S2 shows the normalized MCIs for the non-coordinated and complexed species: the more positive the MCI values, the more aromatic the rings. Thus, when the DBB ligand coordinates to the metal complexes its aromaticity is reduced (same as for benzene, which is opposite to the NICS prediction and incorrect for π-delocalized systems). However, the coordination of P_4 and As_4 induces a charge transfer, resulting in aromatic P_4^{2-} and As_4^{2-} rings, the aromaticity of which decreases upon complexation with Fe^{2+} compared to free P_4^{2-} and As_4^{2-}.

Table S3. Calculated normalized multicenter indices for each cyclic fragment within the geometry of complexes **2-M** and **3-E**.

Complex	MCI	
	DBB	E_4
DBB (free)	0.5719	–
**2-Fe**	0.4893	–
**2-Ru**	0.4910	–
**2-Os**	0.4882	–
P_4 (free)	–	0.2312
P_4^{2-} (free)	–	0.4649
As_4 (free)	–	–0.5155
As_4^{2-} (free)	–	0.4609
**3-P**	0.4908	0.3368
**3-As**	0.4891	0.3418
**(DBB)RuP$_4$**	0.4893	0.3700
**(DBB)RuAs$_4$**	0.4889	0.3745

Table S4. Test of different fragmentation possibilities between the (DBB)$M^{0/2+}$ (M = Fe, Ru, Os) and the $E_4^{0/2-}$ (E = P, As) fragments in complexes **3-M**. Each (DBB)M fragment was calculated with different multiplicities to determine its ground state.

Complex	BDE (kcal mol^{-1})[a]
(DBB)Fe – P_4	110.6
(DBB)Fe – As_4	97.9
(DBB)Ru – P_4	162.1
(DBB)Ru – As_4	162.4
(DBB)Fe^{2+} – P_4^{2-}	396.3
(DBB)Fe^{2+} – As_4^{2-}	389.4
(DBB)Ru^{2+} – P_4^{2-}	412.6
(DBB)Ru^{2+} – As_4^{2-}	408.4

[a]Values contain the BSSE correction.

Table S5. NBO charges of complexes **2-M**, **3-E**, **(DBB)RuE₄**, **4** and **5** summed by fragments.

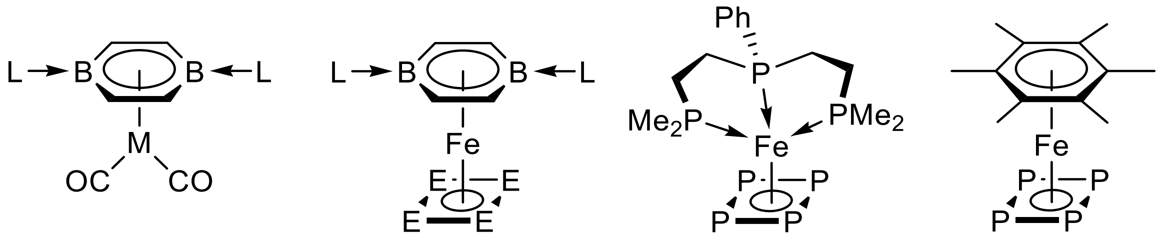

2-M **3-E** **4** **5**

Fragment	2-Fe	2-Ru	2-Os	3-P	3-As	(DBB)RuP$_4$	(DBB)RuAs$_4$
DBB	–0.0786	0.1474	0.0800	0.1927	0.1704	0.5440	0.5069
M	0.2961	–0.3256	–0.1712	0.5844	0.5921	–0.2918	–0.2908
E$_4$	–	–	–	–0.7770	–0.7625	–0.2523	–0.2160
(CO)$_2$	–0.2174	0.1782	0.0911	–	–	–	–

Fragment	4	5
Neutral L	0.572	0.074
Fe	0.215	0.521
P$_4$	–0.788	–0.595

Table S6. Calculated effective oxidation state (EOS) for the metal atom of complexes **3-E**, **(DBB)RuE₄**, **4** and **5**.

	3-P	3-As	(DBB)RuP$_4$	(DBB)RuAs$_4$	4	5
EOS (Metal)	2+	2+	2+	2+	2+	2+
Redox assignment (R)	55.09%	52.74%	53.93%	51.86%	54.89%	62.39%

	2-Fe	2-Ru
EOS (Metal)	0	0
Redox assignment (R)	73.19%	75.11%

Table S7. Calculated IBO bonding mechanism of complexes **3-P**, **4** and **5**.

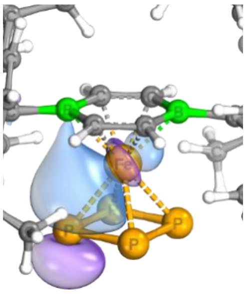<br>σ bond<br>Fe (0.68)<br>P (1.08)	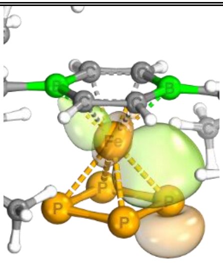<br>σ bond<br>Fe (0.83)<br>P (0.99)	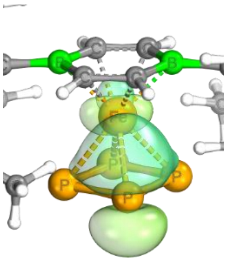<br>σ bond<br>Fe (0.71)<br>P (1.10)
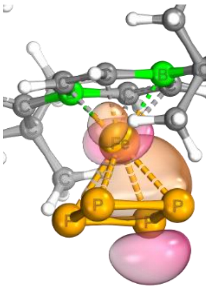<br>σ bond<br>Fe (0.74)<br>P (1.07)	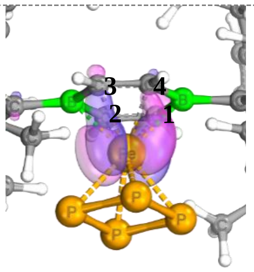<br>d orbital<br>Fe (1.60) C1 (0.15)<br>C2 (0.06)<br>C3 (0.02) C4 (0.04)	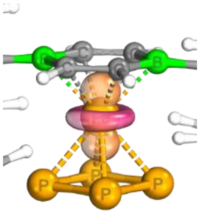<br>d orbital<br>Fe (1.97)

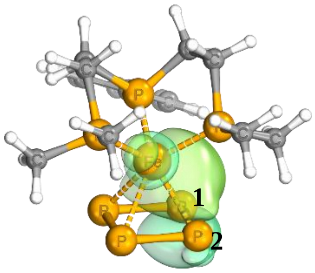<br>σ bond<br>Fe (0.56)<br>P1 (1.19)<br>P2 (0.12)	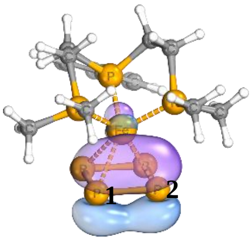<br>π bond<br>Fe (0.37)<br>P1 (0.80)<br>P2 (0.77)

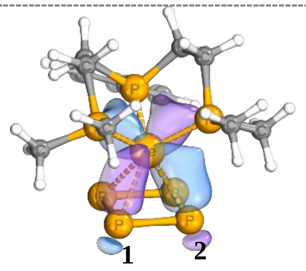<br>π backbonding<br>Fe (1.49)<br>P1 (0.16)<br>P2 (0.17)	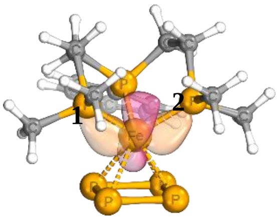<br>d orbital<br>Fe (1.78)	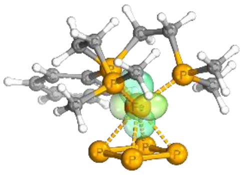<br>d orbital<br>Fe (1.84)
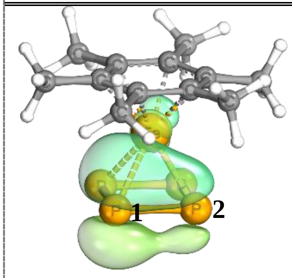<br>π delocalization<br>Fe (0.53)<br>P1 (1.08)<br>P2 (0.31)	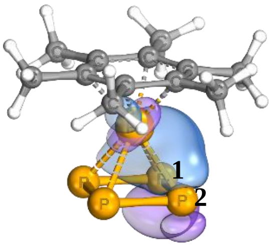<br>π delocalization<br>Fe (0.52)<br>P1 (1.08)<br>P2 (0.32)	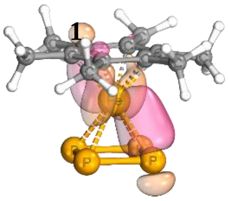<br>σ bond<br>Fe (1.37)<br>P (0.34)<br>C1 (0.13)
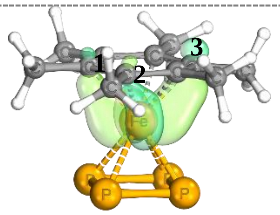<br>d orbital<br>Fe (1.60)<br>C1 (0.08)<br>C2 (0.07) C3 (0.18)	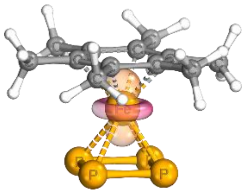<br>d orbital<br>Fe (1.97)	

Intrinsic Bond Orbitals

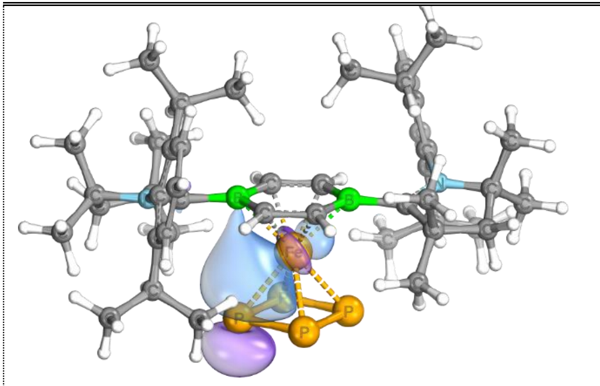<br>Fe (0.68)<br>P (1.08)	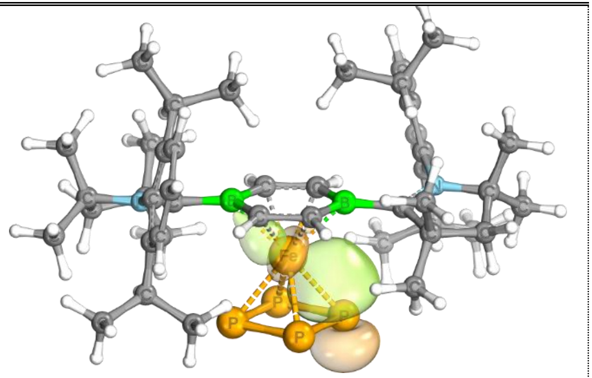<br>Fe (0.83)<br>P (0.99)
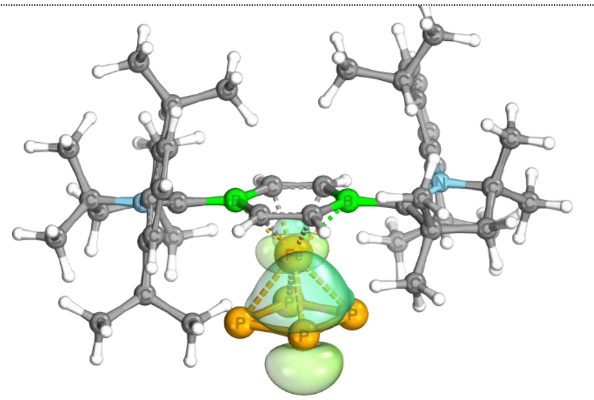<br>Fe (0.71)<br>P (1.10)	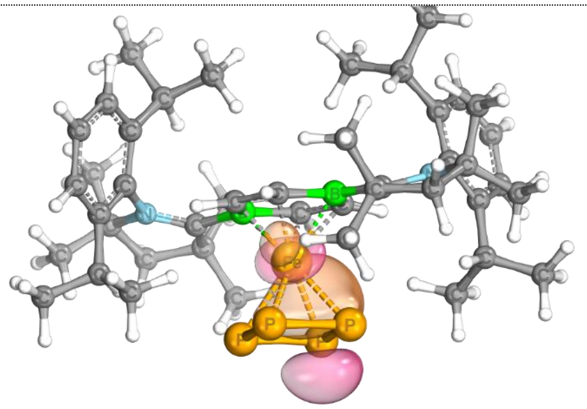<br>Fe (0.74)<br>P (1.07)
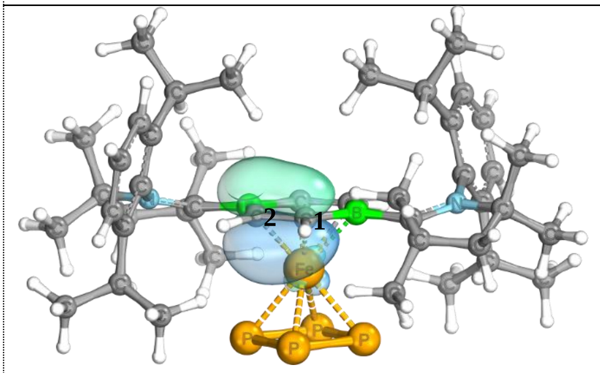<br>Fe (0.18) C1 (0.57)<br>C2 (0.92) B (0.21)	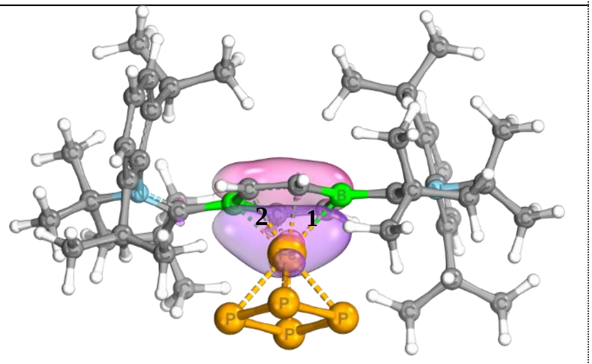<br>Fe (0.19) C1 (0.49)<br>C2 (0.94) B (0.26)

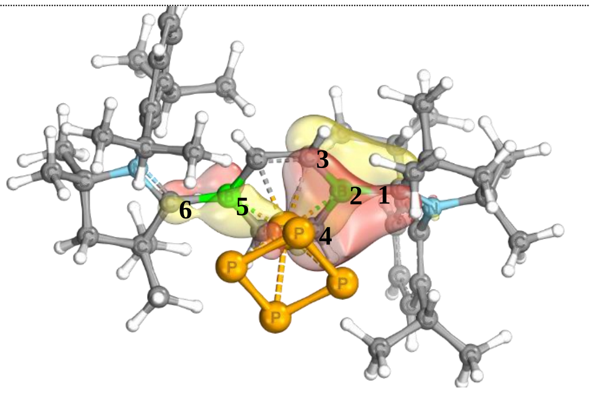

Fe (0.29) C1 (0.15) B2 (0.69)

C3 (0.28) C4 (0.33) B5 (0.12) C6 (0.04)

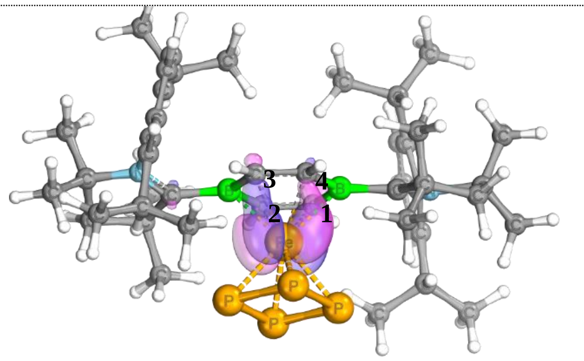

Fe (1.60) C1 (0.15) C2 (0.06)

C3 (0.02) C4 (0.04)

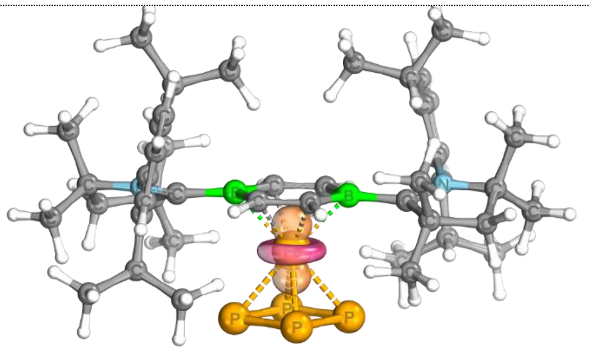

Fe (1.97)

Figure S30. Most important intrinsic bond orbitals of **3-P**.

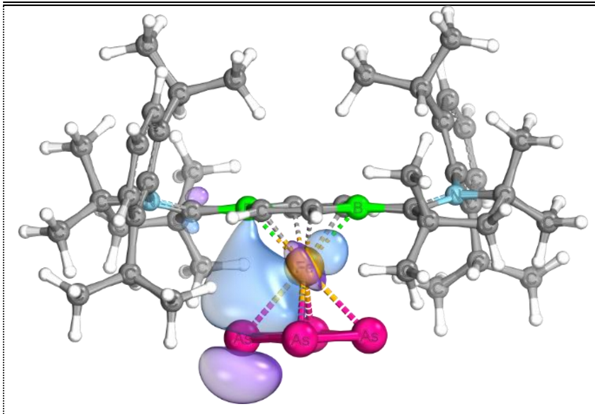<br>Fe (0.70)<br>As (1.06)	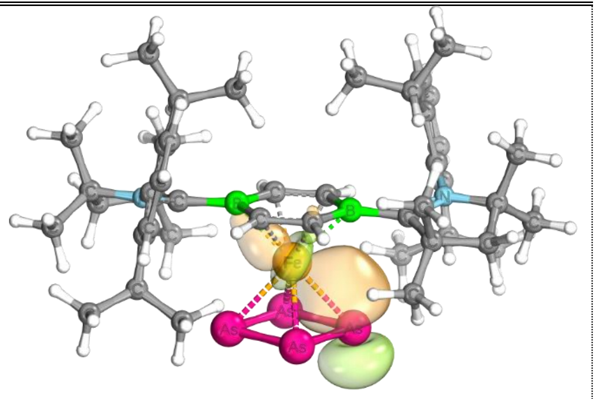<br>Fe (0.88)<br>As (0.94)
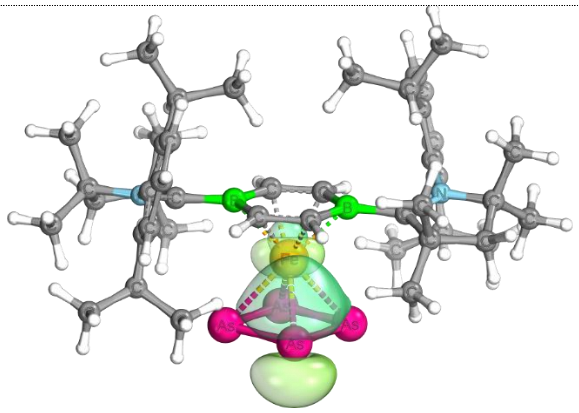<br>Fe (0.69)<br>As (1.11)	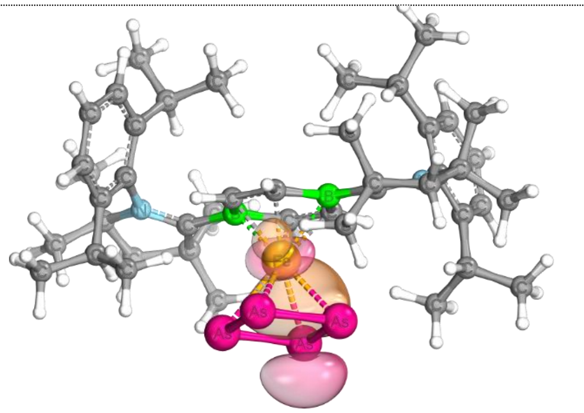<br>Fe (0.71)<br>As (1.09)
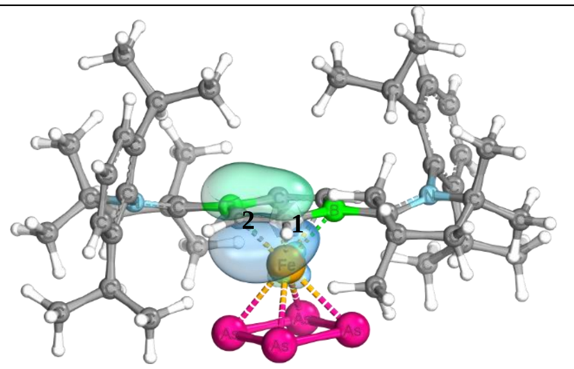<br>Fe (0.18) C1 (0.59)<br>C2 (0.91) B (0.18)	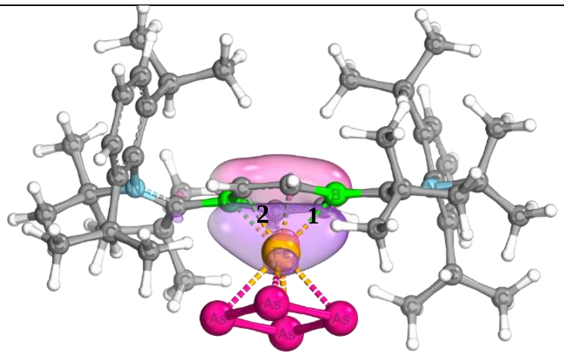<br>Fe (0.19) C1 (0.48)<br>C2 (0.95) B (0.26)

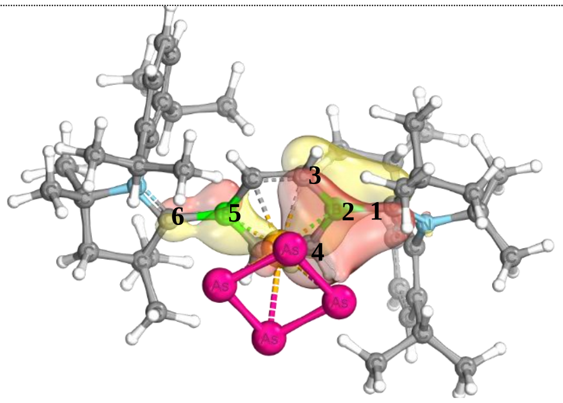 Fe (0.28) C1 (0.16) B2 (0.69)<br>C3 (0.26) C4 (0.34) B5 (0.12) C6 (0.04)	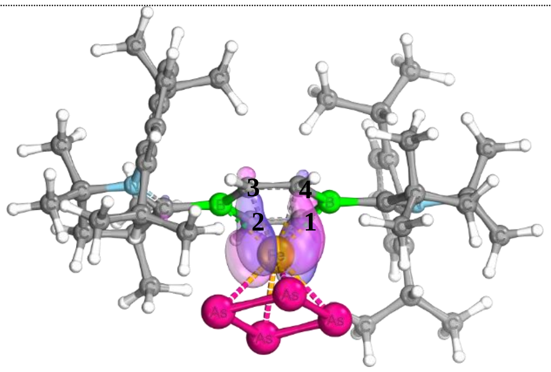 Fe (1.60) C1 (0.15) C2 (0.06)<br>C3 (0.03) C4 (0.05)
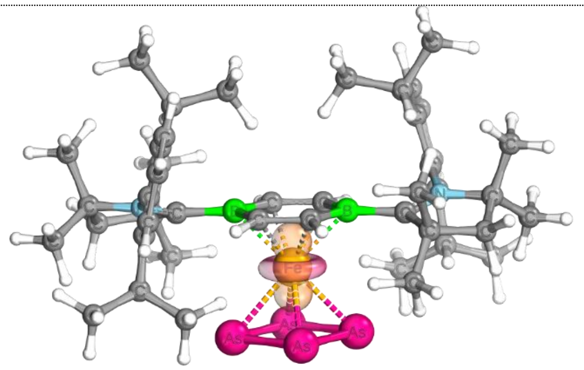 Fe (1.96)	

Figure S31. Most important intrinsic bond orbitals of **3-As**.

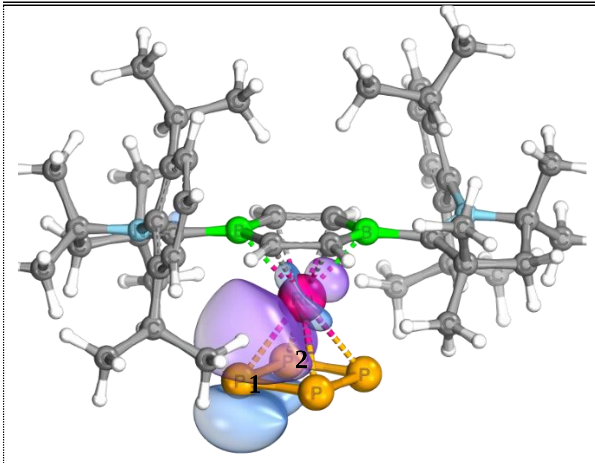<br>Ru (0.43)<br>P1 (1.13) P2 (0.27)	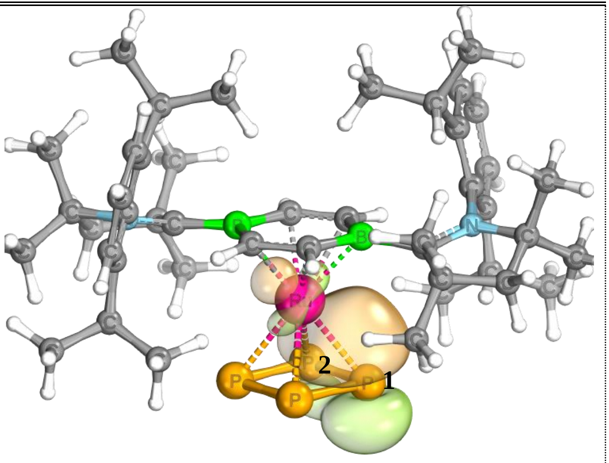<br>Ru (0.47)<br>P1 (1.10) P2 (0.31)
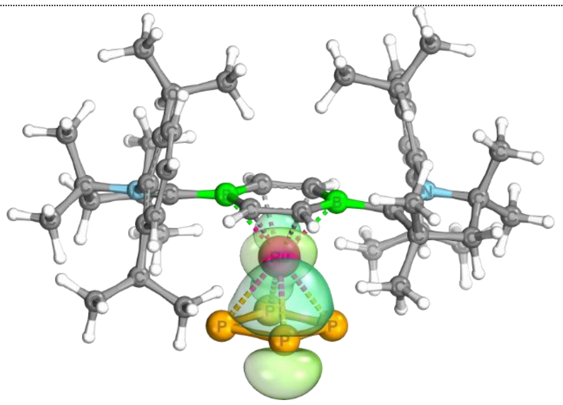<br>Ru (0.77)<br>P (1.04)	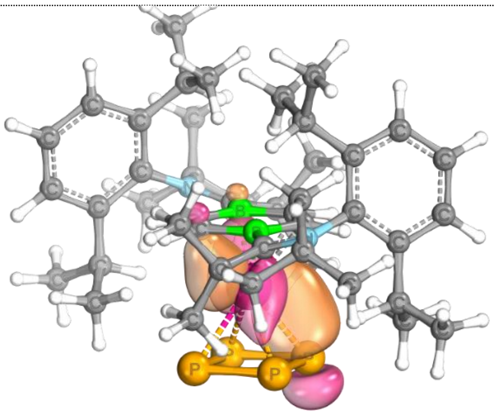<br>Ru (1.32)<br>P (0.43)
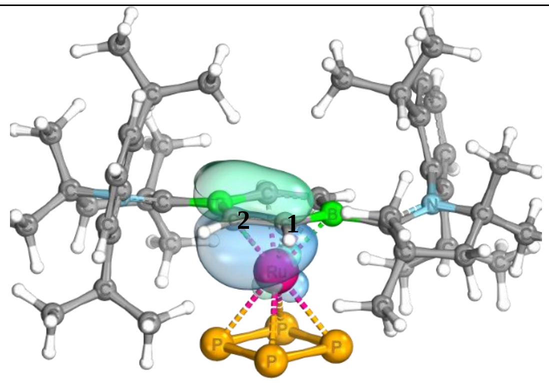<br>Ru (0.20) C1 (0.51)<br>C2 (0.91) B (0.24)	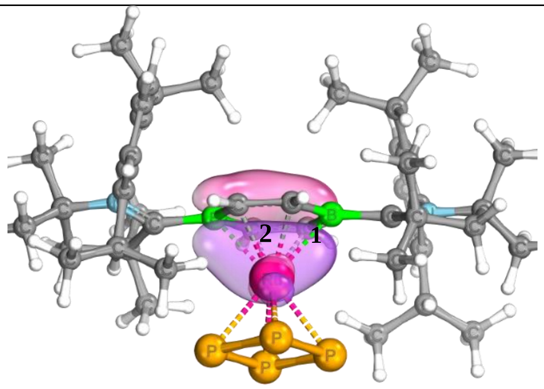<br>Ru (0.17) C1 (0.54)<br>C2 (0.92) B (0.22)

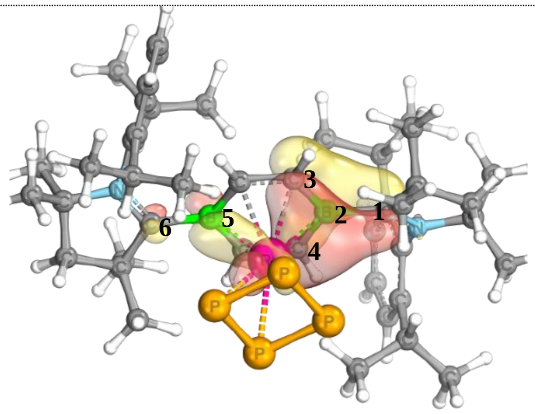

Ru (0.44) C1 (0.14) B2 (0.67)

C3 (0.30) C4 (0.25) B5 (0.10) C6 (0.04)

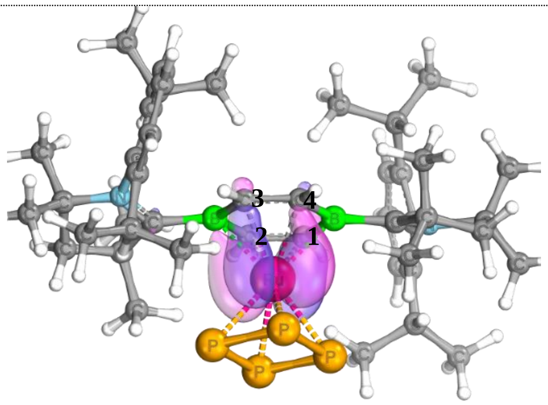

Ru (1.60) C1 (0.16) C2 (0.03)

C3 (0.06) C4 (0.04)

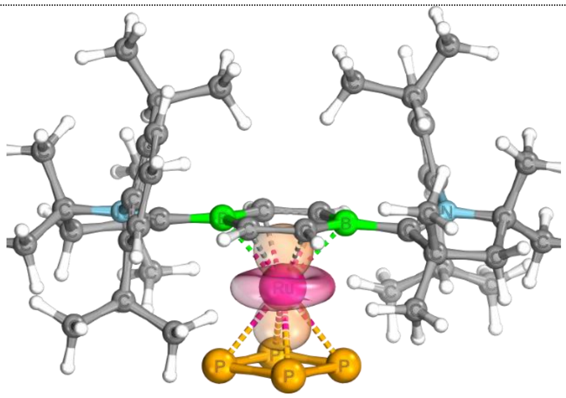

Ru (1.96)

Figure S32. Most important intrinsic bond orbitals of compound **(DBB)RuP$_4$**.

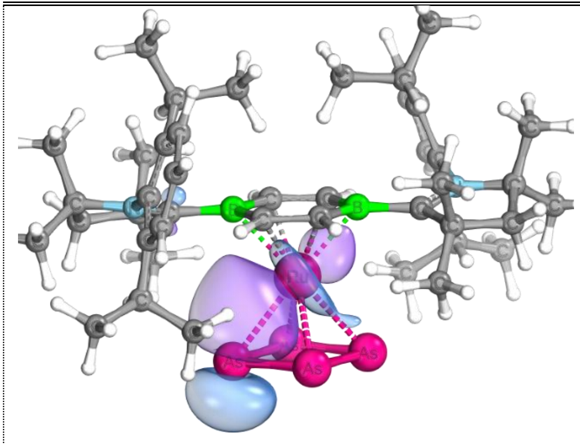<br>Ru (0.80)<br>As (0.99)	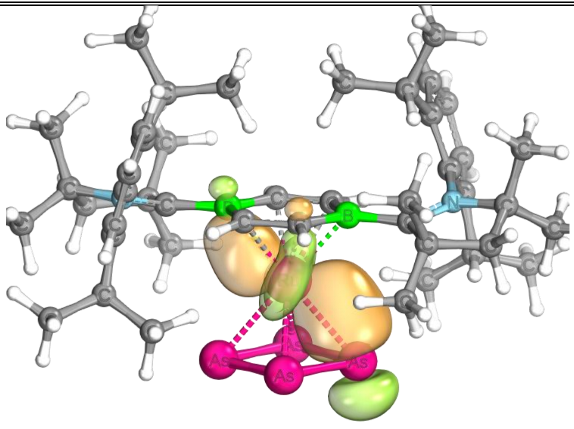<br>Ru (1.24)<br>As (0.51)
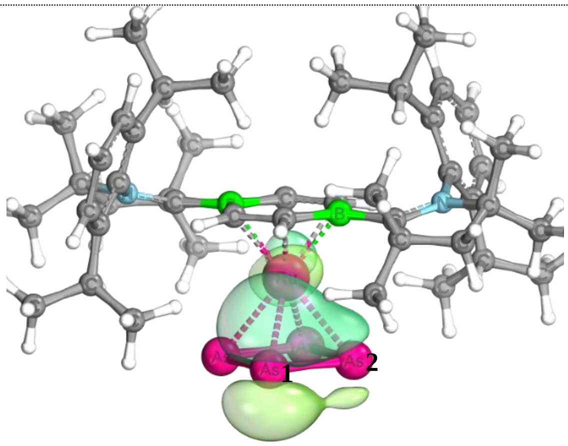<br>Ru (0.56)<br>As1 (1.13) As2 (0.20)	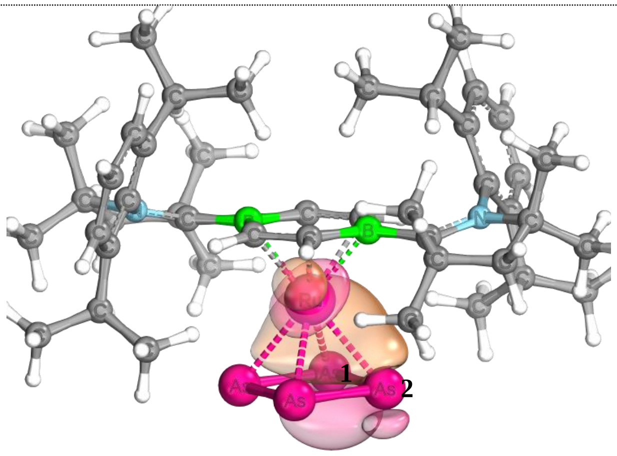<br>Ru (1.12)<br>As1 (0.55) As2 (0.23)
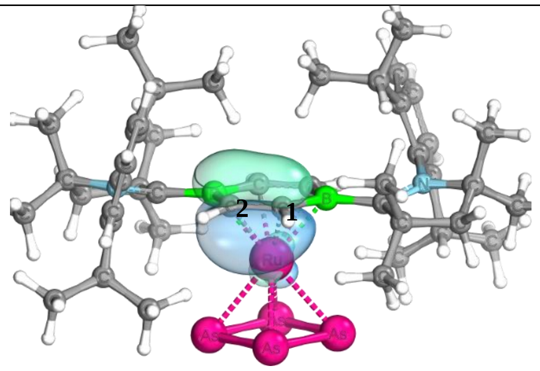<br>Ru (0.20) C1 (0.61)<br>C2 (0.88) B (0.18)	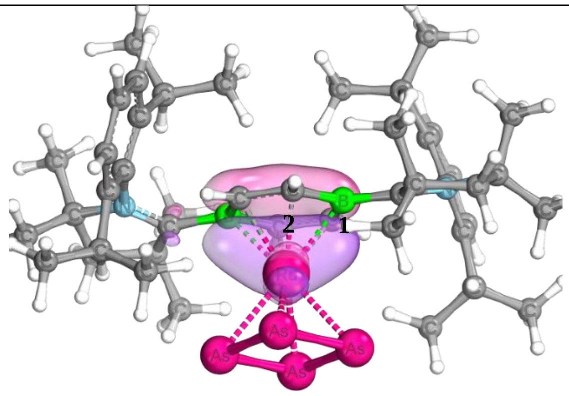<br>Ru (0.22) C1 (0.45)<br>C2 (0.93) B (0.28)

<table>
<tr>
<td>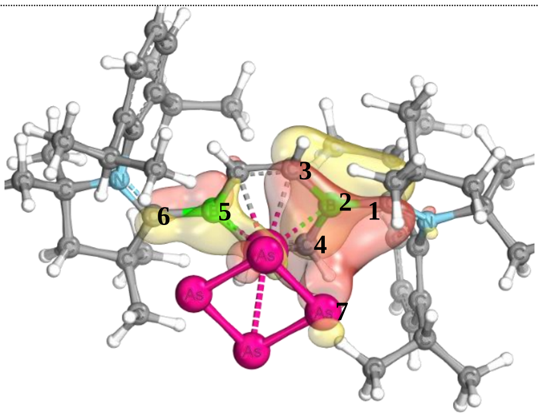

Ru (0.21) C1 (0.18) B2 (0.69)
C3 (0.21) C4 (0.34) B5 (0.10) C6 (0.05) As7 (0.20)</td>
<td>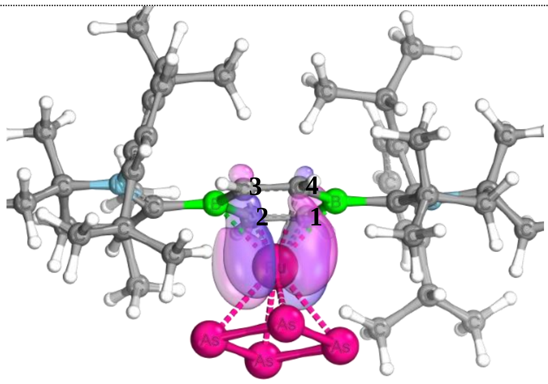

Ru (1.61) C1 (0.15) C2 (0.02)
C3 (0.07) C4 (0.05)</td>
</tr>
<tr>
<td>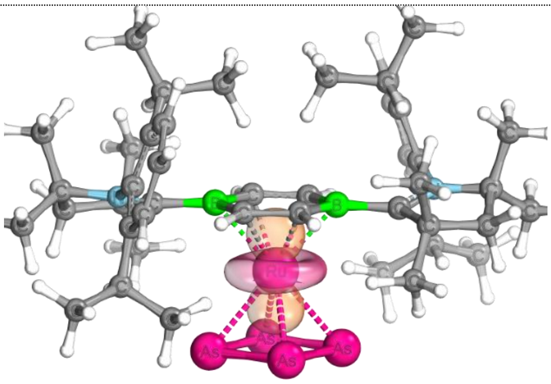
Ru (1.96)</td>
<td></td>
</tr>
</table>

Figure S33. Most important intrinsic bond orbitals of compound **(DBB)RuAs$_4$**.

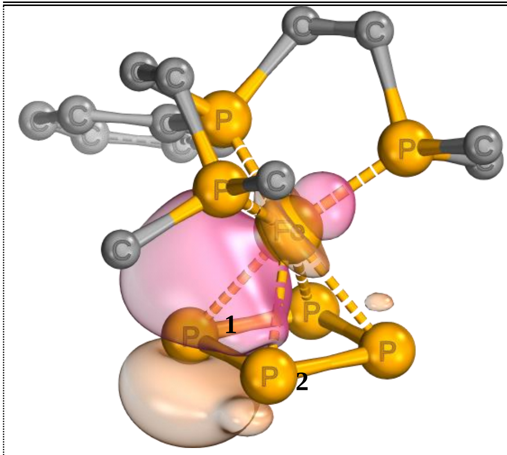 Fe (0.57), P1 (1.18), P2 (0.11)	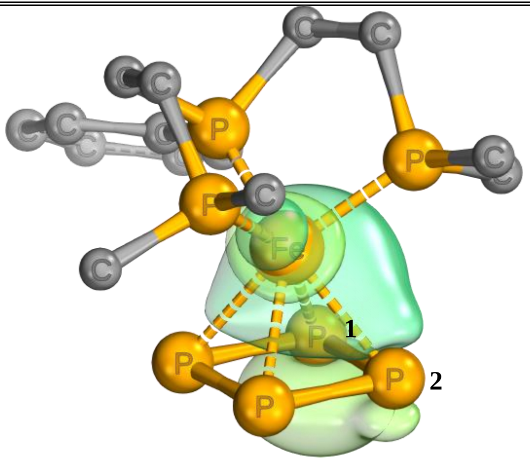 Fe (0.56), P1 (1.19), P2 (0.12)
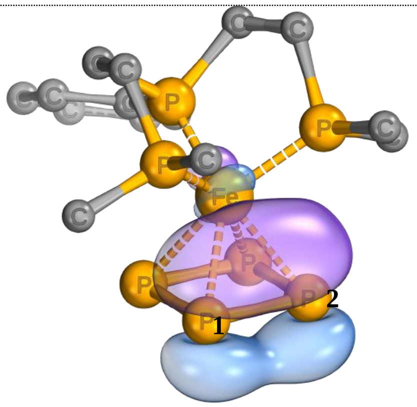 Fe (0.37), P1 (0.79), P2 (0.80)	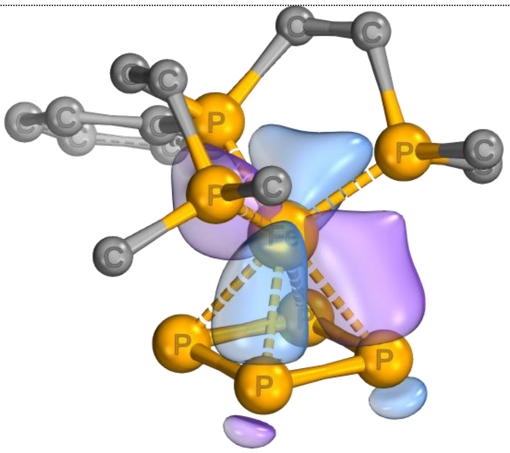 Fe (1.49), P1 (0.17), P2 (0.16)
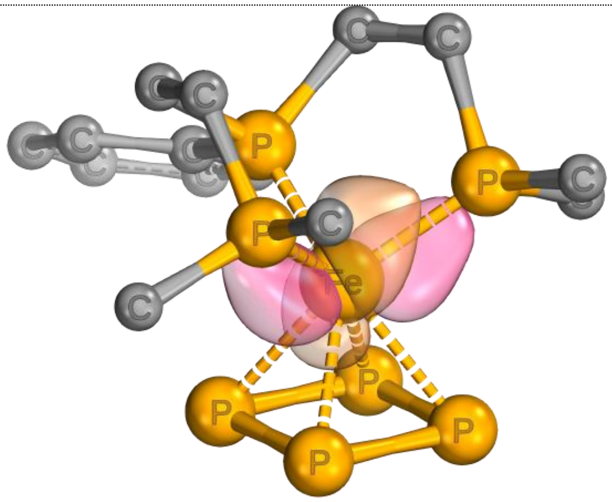 Fe (1.78), P1 (0.06), P2 (0.06)	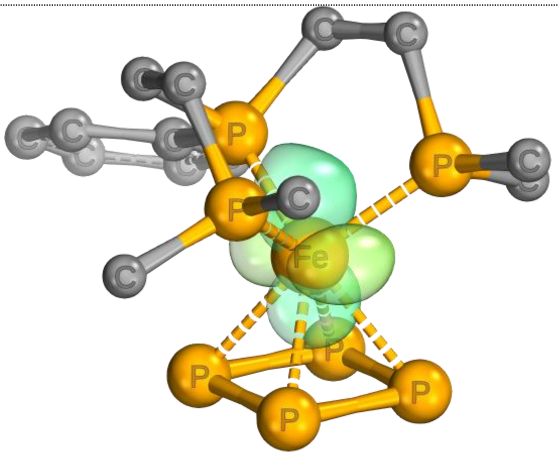 Fe (1.78)

Figure S34. Most important intrinsic bond orbitals of complex **4**.

<table>
<tr>
<td>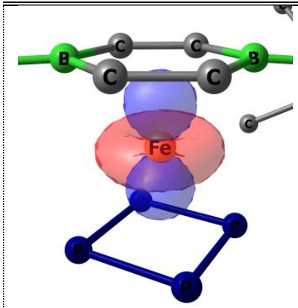

d orbital
(occ. = 1.98e^{-})</td>
<td>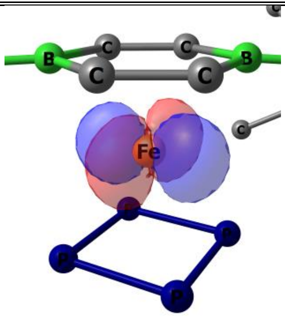

d orbital
(occ. = 1.76e^{-})</td>
<td>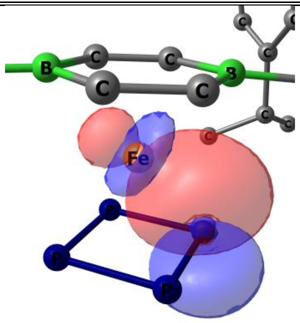

σ(P–Fe)
(occ. = 1.78e^{-})</td>
<td>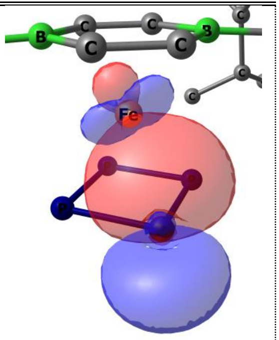

σ(P–Fe)
(occ. = 1.81e^{-})</td>
</tr>
<tr>
<td></td>
<td colspan="2">Fe “Lone Vacant (LV)”
s orbital
(occ. = 0.38e^{-})

P “Lone Pair (LP)”
(occ. = 1.19e^{-})

n(P) → s(Fe)
E(2)= +76.1 kcal/mol
= σ(P–Fe)
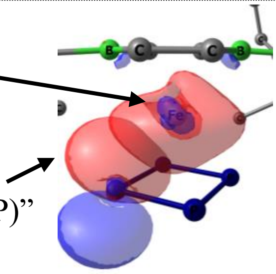
</td>
<td>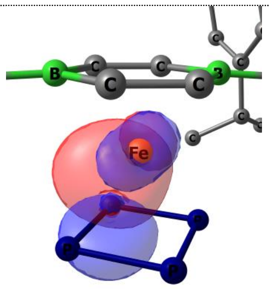

σ(P–Fe)
(occ. = 1.80e^{-})</td>
</tr>
</table>

Figure S35. Most important NBOs of complex **3-P**, along with the relevant NBO donor-acceptor interactions. Isodensity value of 0.03.

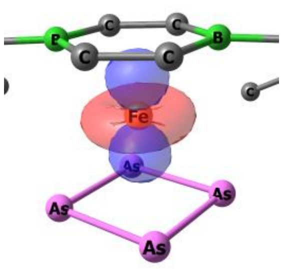

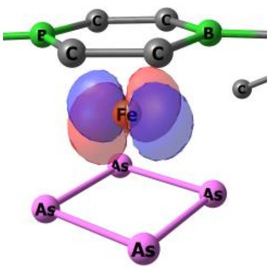

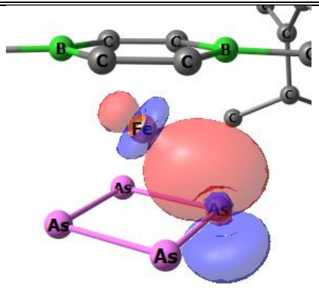

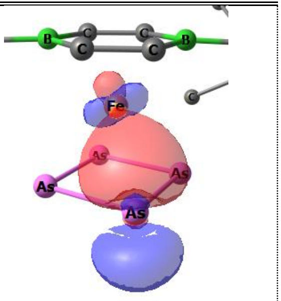

d orbital<br>(occ. = 1.97e⁻)	*d* orbital<br>(occ. = 1.76e⁻)	σ(P–As)<br>(occ. = 1.77e⁻)	σ(P–As)<br>(occ. = 1.82e⁻)
	Fe "Lone Vacant (LV)"<br>*s* orbital<br>(occ. = 0.39e⁻)<br>As "Lone Pair (LP)"<br>(occ. = 1.17e⁻)<br>n(P) → *s*(Fe)<br>E(2)= +76.1 kcal/mol<br>= σ(P–Fe)		σ(P–As)<br>(occ. = 1.81e⁻)

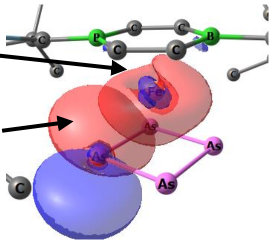

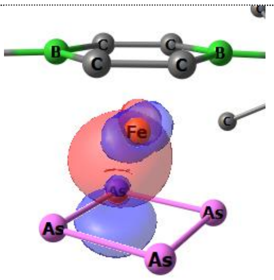

Figure S36. Most important NBOs of complex **3-As**, along with the relevant NBO donor-acceptor interactions. Isodensity value of 0.03. Occupancies (occ.) are given in electrons.

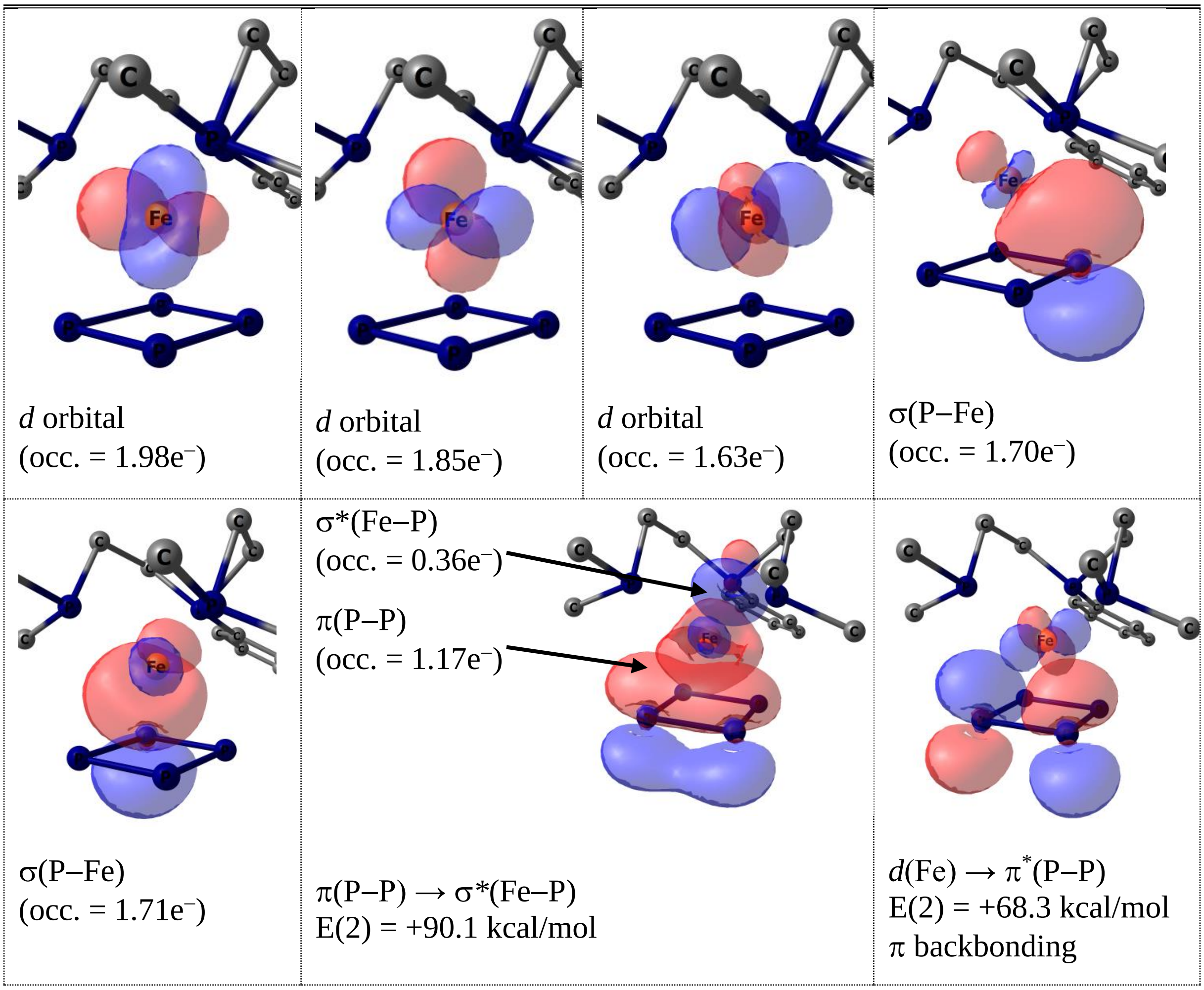

Figure S37. Most important NBOs of complex **4**, along with the relevant NBO donor-acceptor interactions. Isodensity value of 0.03. Occupancies (occ.) are given in electrons.

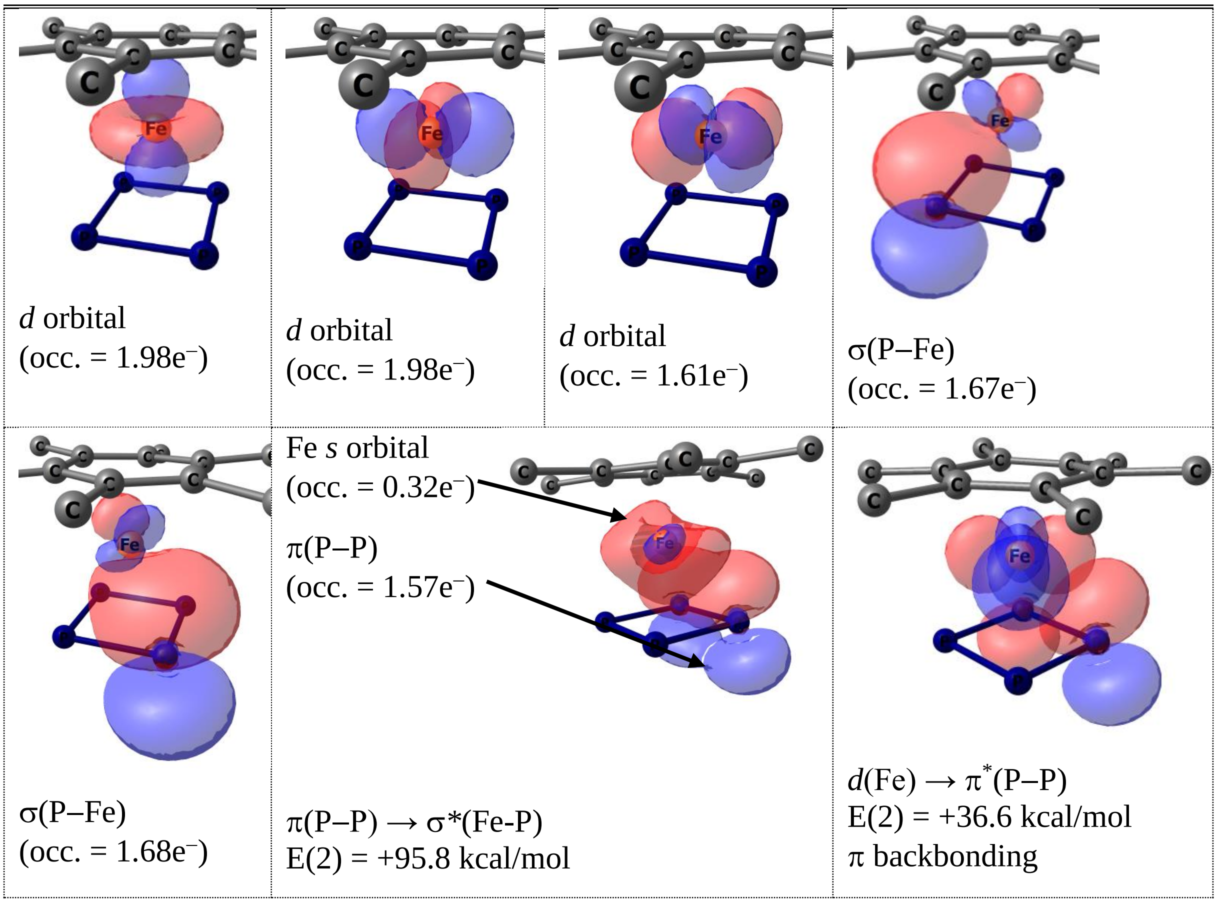

Figure S38. Most important NBOs of complex **5**, along with the relevant NBO donor-acceptor interactions. Isodensity value of 0.03. Occupancies (occ.) are given in electrons.

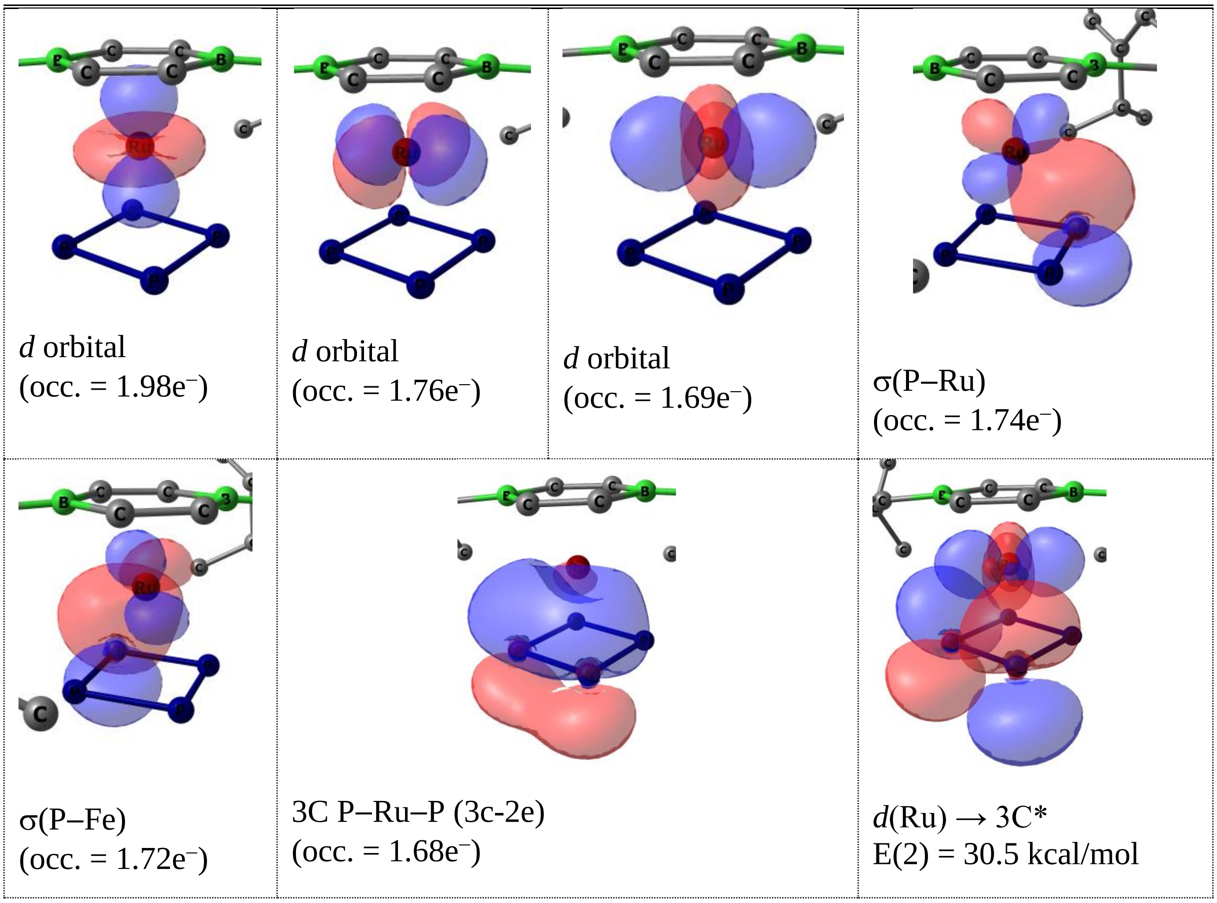

Figure S39. Most important NBOs of complex **(DBB)RuP$_4$**, along with the relevant NBO donor-acceptor interactions. Isodensity value of 0.03. Occupancies (occ.) are given in electrons.

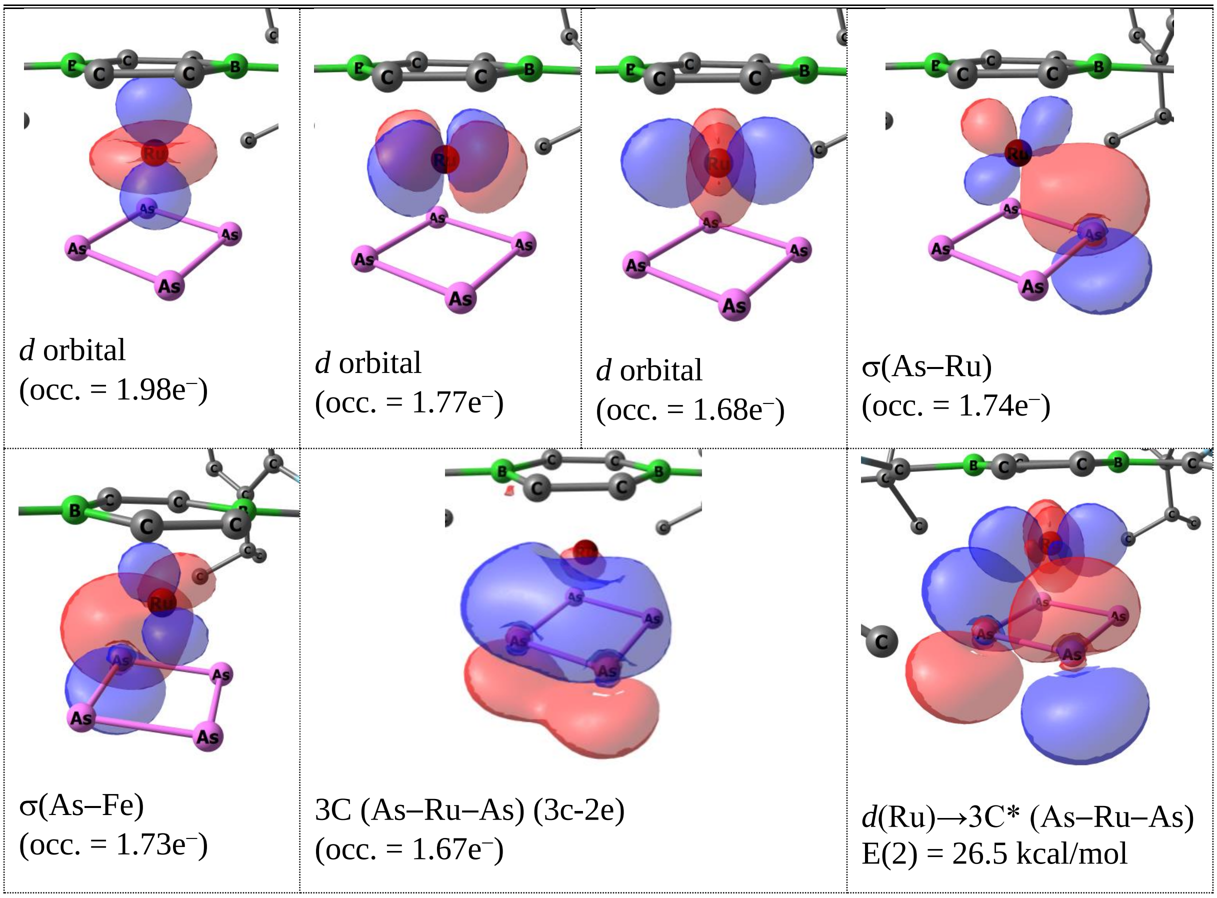

Figure S40. Most important NBOs of complex **(DBB)RuAs₄**, along with the relevant NBO donor-acceptor interactions. Isodensity value of 0.03. Occupancies (occ.) are given in electrons.

Table S7. Optimized geometries of the studied complexes, with different oxidation states and multiplicities, and fragments with different symmetries and charges as indicated, calculated at the ωB97X-D/def2-svpp level of theory.

2-Fe (*singlet*)

E(scf) = -3363.73354124 a.u.

B	-1.419129	-0.063880	-0.591539
O	0.046179	-3.703278	-2.024449
B	1.419171	-0.063649	0.591414
O	-0.048366	-3.701487	2.026527
C	-0.141518	-0.066465	-1.447894
H	-0.179291	-0.227347	-2.531464
C	0.141553	-0.066101	1.447749
H	0.179352	-0.226715	2.531358
C	1.161637	-0.048107	-0.914529
H	1.960031	-0.170359	-1.652295
C	-1.161615	-0.047988	0.914377
H	-1.960019	-0.170232	1.652148
Fe	0.000067	-1.625236	0.000152
C	0.034508	-2.876073	-1.212296
C	-0.035433	-2.875121	1.213548
C	-2.835608	-0.083738	-1.278820
N	-3.986682	0.236510	-0.703038
C	-5.180883	0.298195	-1.617863
C	-4.639404	-0.462353	-2.829216
H	-5.038683	-0.067880	-3.777193
H	-4.945728	-1.519618	-2.757633
C	-3.100002	-0.374015	-2.758533
C	-5.540987	1.756193	-1.928609
H	-6.377371	1.775108	-2.646910
H	-4.705577	2.319438	-2.367596
H	-5.869154	2.274211	-1.013862
C	-6.429799	-0.365176	-1.036138
H	-7.258610	-0.229174	-1.751027
H	-6.727384	0.096639	-0.080204
H	-6.299980	-1.445497	-0.883826
C	-2.573966	0.784928	-3.630421

2-Fe (*triplet*)

E(scf) = -3363.72679219 a.u.

B	-1.439349	-0.151614	-0.624428
O	0.460866	-3.856408	-2.234750
B	1.423216	-0.160416	0.622716
O	-0.504813	-3.995471	1.981157
C	-0.143153	-0.314223	-1.452226
H	-0.164777	-0.454321	-2.539252
C	0.129221	-0.400606	1.437153
H	0.153463	-0.606217	2.513618
C	1.153027	-0.311105	-0.894050
H	1.962017	-0.460167	-1.617130
C	-1.168293	-0.375420	0.880861
H	-1.975197	-0.570412	1.595256
Fe	-0.002904	-1.958421	-0.057501
C	0.287300	-3.127517	-1.366379
C	-0.315627	-3.210365	1.166766
C	-2.800619	0.097185	-1.298096
N	-3.938426	0.511359	-0.674768
C	-5.038592	0.933715	-1.586127
C	-4.619831	0.218284	-2.875689
H	-4.927899	0.773225	-3.776790
H	-5.114929	-0.767340	-2.913193
C	-3.087312	0.017926	-2.800998
C	-5.073910	2.460598	-1.755777
H	-5.829992	2.728923	-2.513298
H	-4.106244	2.868466	-2.082554
H	-5.355213	2.950636	-0.810958
C	-6.423328	0.494708	-1.103188
H	-7.185173	0.887046	-1.797742
H	-6.643803	0.892331	-0.097969
H	-6.530791	-0.598980	-1.077901
C	-2.346071	1.111198	-3.594674

H	-2.873345	0.613861	-4.679466	H	-2.646419	1.073565	-4.657577
H	-1.478475	0.856218	-3.590485	H	-1.255266	0.979842	-3.542659
H	-2.978773	1.760155	-3.315612	H	-2.570473	2.119588	-3.212449
C	-2.491075	-1.703185	-3.224686	C	-2.735630	-1.366401	-3.372877
H	-2.886532	-1.948416	-4.227187	H	-3.119799	-1.454271	-4.405930
H	-2.753161	-2.523059	-2.535959	H	-3.192937	-2.166307	-2.765202
H	-1.395744	-1.668276	-3.285800	H	-1.651046	-1.548133	-3.398057
C	-4.117236	0.610478	0.681748	C	-4.058984	0.573448	0.749154
C	-4.497099	-0.382694	1.607846	C	-4.554589	-0.565104	1.422403
C	-4.640001	-0.006621	2.946094	C	-4.644852	-0.526203	2.815974
H	-4.925784	-0.758302	3.686799	H	-5.019050	-1.399803	3.357221
C	-4.396253	1.297820	3.358140	C	-4.242748	0.596605	3.530636
H	-4.510940	1.570560	4.411074	H	-4.317285	0.608334	4.621849
C	-3.977690	2.247366	2.434619	C	-3.725622	1.694759	2.855348
H	-3.749198	3.260844	2.776185	H	-3.383584	2.561637	3.428259
C	-3.815929	1.928469	1.083132	C	-3.610902	1.705869	1.461360
C	-4.659251	-1.853202	1.241401	C	-4.910093	-1.857877	0.698743
H	-4.633147	-1.938717	0.146225	H	-4.913668	-1.650787	-0.380059
C	-5.992010	-2.433786	1.732602	C	-6.300092	-2.379452	1.083193
H	-6.142279	-3.443214	1.311816	H	-6.583503	-3.225183	0.432431
H	-6.853215	-1.808141	1.443497	H	-7.071249	-1.596364	0.986691
H	-6.005817	-2.536107	2.831825	H	-6.323704	-2.746938	2.124110
C	-3.478959	-2.687385	1.757195	C	-3.837832	-2.932196	0.922143
H	-3.640927	-3.755167	1.527155	H	-4.125298	-3.871562	0.416902
H	-3.360764	-2.590339	2.851151	H	-3.698206	-3.152245	1.995591
H	-2.539662	-2.378801	1.273559	H	-2.870640	-2.606277	0.509153
C	-3.247043	2.993670	0.153156	C	-2.953568	2.908543	0.798251
H	-3.204785	2.576266	-0.862298	H	-2.946397	2.732474	-0.285359
C	-1.804807	3.359820	0.535686	C	-1.487485	3.073181	1.225325
H	-1.400140	4.091430	-0.186046	H	-1.044697	3.945155	0.712221
H	-1.153054	2.472851	0.530554	H	-0.890720	2.184569	0.968400
H	-1.762689	3.818603	1.539902	H	-1.400320	3.245148	2.313226
C	-4.116560	4.258338	0.112941	C	-3.730722	4.203814	1.073979
H	-3.739436	4.954425	-0.656664	H	-3.326248	5.031778	0.465523
H	-4.089567	4.791990	1.079234	H	-3.646226	4.500826	2.134458
H	-5.172321	4.037753	-0.114055	H	-4.804868	4.103139	0.845566

N	3.986807	0.236170	0.702875	N	3.924232	0.513989	0.701563
C	2.835614	-0.083714	1.278693	C	2.780805	0.068608	1.307528
C	5.181043	0.297532	1.617698	C	5.005188	0.924440	1.636716
C	4.639254	-0.462426	2.829280	C	4.600584	0.138000	2.889656
H	5.038555	-0.067674	3.777133	H	4.899408	0.652266	3.817772
H	4.945322	-1.519794	2.758150	H	5.114039	-0.838975	2.877543
C	3.099893	-0.373733	2.758474	C	3.071219	-0.085689	2.804347
C	5.541903	1.755431	1.928037	C	4.995277	2.441750	1.885339
H	6.378443	1.774132	2.646155	H	5.723126	2.690834	2.676651
H	4.706849	2.319172	2.367073	H	4.007118	2.804187	2.205331
H	5.870139	2.273062	1.013090	H	5.286890	2.989586	0.976159
C	6.429646	-0.366598	1.036204	C	6.402699	0.548001	1.139240
H	7.258430	-0.230965	1.751185	H	7.151836	0.913715	1.861744
H	6.727616	0.094965	0.080266	H	6.619402	1.011070	0.161536
H	6.299245	-1.446872	0.884001	H	6.534103	-0.539593	1.046186
C	2.574029	0.785617	3.629941	C	2.311485	0.942375	3.664274
H	2.873276	0.614841	4.679073	H	2.604653	0.838554	4.724968
H	1.478547	0.857104	3.589872	H	1.222594	0.801147	3.595540
H	2.979053	1.760664	3.314857	H	2.525503	1.976579	3.351516
C	2.490660	-1.702590	3.225112	C	2.748042	-1.508122	3.295650
H	2.886148	-1.947572	4.227661	H	3.133194	-1.649419	4.322582
H	2.752475	-2.522765	2.536642	H	3.221494	-2.261861	2.642715
H	1.395349	-1.667380	3.286354	H	1.667021	-1.712661	3.308560
C	4.117455	0.610257	-0.681878	C	4.070124	0.575248	-0.718195
C	4.497266	-0.382811	-1.608123	C	4.556907	-0.572104	-1.384059
C	4.640310	-0.006535	-2.946305	C	4.666191	-0.537965	-2.776263
H	4.926056	-0.758139	-3.687104	H	5.032561	-1.418830	-3.311139
C	4.396747	1.297995	-3.358168	C	4.295622	0.591263	-3.498358
H	4.511566	1.570910	-4.411043	H	4.385734	0.599630	-4.588472
C	3.978191	2.247432	-2.434532	C	3.792298	1.700923	-2.831598
H	3.749822	3.260986	-2.775957	H	3.477143	2.574496	-3.409943
C	3.816283	1.928345	-1.083113	C	3.657023	1.714656	-1.439321
C	4.659118	-1.853419	-1.241957	C	4.887982	-1.865894	-0.651099
H	4.632838	-1.939113	-0.146802	H	4.891034	-1.651100	0.426363
C	5.991797	-2.434174	-1.733193	C	6.271148	-2.412621	-1.025194
H	6.141722	-3.443820	-1.312804	H	6.537123	-3.258959	-0.367853

H	6.853152	-1.808901	-1.443748
H	6.005688	-2.536034	-2.832461
C	3.478769	-2.687324	-1.758081
H	3.640490	-3.755151	-1.528083
H	3.360863	-2.590168	-2.852061
H	2.539438	-2.378630	-1.274580
C	3.247371	2.993480	-0.153071
H	3.205079	2.575995	0.862349
C	1.805160	3.359662	-0.535673
H	1.400455	4.091275	0.186036
H	1.153388	2.472704	-0.530605
H	1.763128	3.818444	-1.539892
C	4.116848	4.258168	-0.112706
H	3.739582	4.954214	0.656867
H	4.089968	4.791856	-1.078983
H	5.172583	4.037621	0.114446

3-P (*singlet*)

E(scf) = -4502.35460658 a.u.

B	-1.394851	0.153632	-0.616312
Fe	-0.000422	-1.376821	-0.000018
P	-1.505022	-3.187449	-0.242884
P	0.245649	-3.189433	-1.513338
B	1.394780	0.153019	0.616311
C	-0.126648	0.139819	-1.464496
H	-0.143138	-0.023136	-2.546972
P	1.505891	-3.186184	0.242613
C	-1.160504	0.145032	0.890290
H	-1.980768	-0.005773	1.595603
C	0.126558	0.139675	1.464480
H	0.142989	-0.023393	2.546941
P	-0.244796	-3.189800	1.513089
C	1.160391	0.144572	-0.890279
H	1.980590	-0.006739	-1.595566
N	-3.971287	0.375772	-0.717175
C	-2.826687	0.065482	-1.294265

H	7.054469	-1.641496	-0.928898
H	6.294882	-2.786495	-2.063874
C	3.799923	-2.924029	-0.873842
H	4.063922	-3.861856	-0.353033
H	3.670408	-3.154826	-1.946436
H	2.832538	-2.576117	-0.479005
C	3.015971	2.929169	-0.783844
H	2.983025	2.746092	0.298003
C	1.563233	3.131195	-1.238459
H	1.130321	4.010146	-0.728571
H	0.942226	2.254371	-0.998007
H	1.500717	3.311514	-2.326788
C	3.832581	4.204511	-1.036506
H	3.439856	5.040465	-0.431166
H	3.778018	4.508810	-2.097014
H	4.898935	4.071564	-0.787905

3-P (*triplet*)

E(scf) = -4502.30492333 a.u.

B	-1.426350	0.133711	-0.636232
Fe	-0.000083	-1.453913	0.000229
P	-1.525174	-3.222886	-0.267687
P	0.263685	-3.219349	-1.511696
B	1.426375	0.133784	0.636185
C	-0.118824	0.085534	-1.466334
H	-0.131477	-0.074276	-2.549181
P	1.525793	-3.222173	0.268442
C	-1.162673	0.095681	0.888052
H	-1.989928	-0.048395	1.587980
C	0.118835	0.086003	1.466291
H	0.131474	-0.073421	2.549192
P	-0.263091	-3.219203	1.512456
C	1.162688	0.095251	-0.888096
H	1.989946	-0.049166	-1.587958
N	-4.009233	0.388367	-0.705886
C	-2.827617	0.085353	-1.302180

C	-5.180024	0.349033	-1.623347	C	-5.190733	0.412338	-1.623722
C	-4.612910	-0.388416	-2.836882	C	-4.623396	-0.301926	-2.853610
H	-5.028580	-0.003922	-3.781787	H	-5.041006	0.099723	-3.791256
H	-4.878516	-1.456234	-2.768997	H	-4.894474	-1.369879	-2.804772
C	-3.081073	-0.244425	-2.770011	C	-3.088992	-0.168165	-2.789018
C	-5.646328	1.773752	-1.939543	C	-5.643070	1.847894	-1.923692
H	-6.507084	1.720771	-2.626485	H	-6.499142	1.820856	-2.618797
H	-4.868351	2.382913	-2.420849	H	-4.853464	2.456586	-2.386448
H	-5.977073	2.286343	-1.022094	H	-5.974548	2.348808	-0.999675
C	-6.359850	-0.404012	-1.018148	C	-6.398360	-0.336815	-1.063275
H	-7.179642	-0.410269	-1.756277	H	-7.210050	-0.310491	-1.810242
H	-6.729853	0.088591	-0.104763	H	-6.772392	0.137836	-0.141418
H	-6.107090	-1.449072	-0.790064	H	-6.167034	-1.391905	-0.856870
C	-2.600503	0.951221	-3.621702	C	-2.601523	1.031365	-3.628870
H	-2.870566	0.768916	-4.676497	H	-2.866675	0.876444	-4.690422
H	-1.511042	1.080353	-3.560794	H	-1.509895	1.152858	-3.562518
H	-3.065674	1.901080	-3.312605	H	-3.057777	1.979072	-3.300942
C	-2.423971	-1.536085	-3.270254	C	-2.456881	-1.459044	-3.328977
H	-2.777461	-1.738439	-4.297344	H	-2.814926	-1.639594	-4.359002
H	-2.704416	-2.390985	-2.632592	H	-2.748043	-2.324100	-2.709046
H	-1.326842	-1.481256	-3.286019	H	-1.358314	-1.420326	-3.356486
C	-4.114161	0.897502	0.625785	C	-4.133411	0.892691	0.634413
C	-4.537232	0.042548	1.667085	C	-4.561867	0.032375	1.672246
C	-4.758936	0.611444	2.925283	C	-4.746828	0.573622	2.948570
H	-5.090033	-0.028179	3.747801	H	-5.084629	-0.075615	3.761358
C	-4.538265	1.961394	3.162092	C	-4.481974	1.910402	3.214304
H	-4.716476	2.381438	4.155987	H	-4.630393	2.311846	4.220826
C	-4.056639	2.767844	2.139510	C	-3.997844	2.726437	2.200200
H	-3.839575	3.819662	2.344956	H	-3.751491	3.768196	2.424556
C	-3.825872	2.261047	0.857646	C	-3.807873	2.243440	0.902217
C	-4.661008	-1.472961	1.542811	C	-4.731067	-1.474341	1.508736
H	-4.447771	-1.756778	0.500921	H	-4.534952	-1.726298	0.455781
C	-6.053346	-1.993420	1.932617	C	-6.135201	-1.965831	1.891106
H	-6.132459	-3.067423	1.690831	H	-6.245746	-3.033396	1.632212
H	-6.872963	-1.465804	1.421109	H	-6.936909	-1.406687	1.385283
H	-6.222077	-1.891267	3.019125	H	-6.303045	-1.874179	2.978838

C	-3.608834	-2.181729	2.409092	C	-3.694250	-2.237191	2.346758
H	-3.660748	-3.271856	2.246078	H	-3.768119	-3.320807	2.148534
H	-3.778390	-1.989607	3.483733	H	-3.855466	-2.080345	3.428254
H	-2.586437	-1.860900	2.162237	H	-2.666485	-1.921420	2.113045
C	-3.202749	3.195770	-0.173719	C	-3.195267	3.188526	-0.124842
H	-3.126287	2.659856	-1.129543	H	-3.155899	2.666326	-1.089632
C	-1.770763	3.578332	0.229249	C	-1.747028	3.539509	0.243981
H	-1.305826	4.189321	-0.564476	H	-1.293177	4.160771	-0.548228
H	-1.148438	2.684091	0.383569	H	-1.133324	2.633968	0.360531
H	-1.765310	4.173067	1.160332	H	-1.701409	4.111157	1.188458
C	-4.037617	4.462201	-0.409932	C	-4.013606	4.473601	-0.312043
H	-3.605327	5.049581	-1.238847	H	-3.597317	5.069250	-1.143467
H	-4.041893	5.111771	0.482764	H	-3.980483	5.106560	0.592169
H	-5.086026	4.236323	-0.662133	H	-5.072464	4.268387	-0.536422
N	3.971114	0.375815	0.717203	N	4.009238	0.388408	0.705690
C	2.826625	0.065149	1.294293	C	2.827648	0.085550	1.302148
C	5.179877	0.349313	1.623399	C	5.190835	0.412433	1.623383
C	4.612954	-0.388540	2.836777	C	4.623472	-0.301193	2.853627
H	5.028663	-0.004285	3.781765	H	5.041235	0.100773	3.791070
H	4.878649	-1.456316	2.768548	H	4.894357	-1.369214	2.805207
C	3.081108	-0.244693	2.770039	C	3.089096	-0.167220	2.789114
C	5.645763	1.774093	1.939891	C	5.643658	1.847977	1.922693
H	6.506364	1.721214	2.627045	H	6.499399	1.820992	2.618207
H	4.867540	2.383028	2.421079	H	4.854093	2.457333	2.384647
H	5.976614	2.286884	1.022597	H	5.975809	2.348139	0.998509
C	6.359950	-0.403249	1.018116	C	6.398158	-0.337322	1.063103
H	7.179684	-0.409465	1.756313	H	7.210000	-0.310871	1.809901
H	6.729902	0.089688	0.104892	H	6.772147	0.136748	0.140931
H	6.107494	-1.448318	0.789756	H	6.166470	-1.392440	0.857232
C	2.600521	0.950920	3.621778	C	2.601960	1.032874	3.628349
H	2.870862	0.768699	4.676512	H	2.867128	0.878452	4.689969
H	1.511023	1.079838	3.561128	H	1.510365	1.154597	3.561981
H	3.065404	1.900881	3.312574	H	3.058441	1.980290	3.299897
C	2.424057	-1.536315	3.270405	C	2.456879	-1.457676	3.329957
H	2.777384	-1.738457	4.297590	H	2.815093	-1.637660	4.360021
H	2.704666	-2.391332	2.632977	H	2.747808	-2.323153	2.710507

H	1.326914	-1.481537	3.285974
C	4.113896	0.897688	-0.625723
C	4.537388	0.043056	-1.667123
C	4.759182	0.612261	-2.925170
H	5.090607	-0.027130	-3.747734
C	4.538271	1.962196	-3.161757
H	4.716637	2.382479	-4.155523
C	4.056242	2.768347	-2.139109
H	3.839067	3.820188	-2.344343
C	3.825296	2.261224	-0.857415
C	4.661705	-1.472457	-1.543114
H	4.448471	-1.756532	-0.501291
C	6.054254	-1.992325	-1.932929
H	6.133782	-3.066295	-1.691152
H	6.873643	-1.464379	-1.421401
H	6.222930	-1.890085	-3.019442
C	3.609938	-2.181414	-2.409716
H	3.662182	-3.271563	-2.246970
H	3.779599	-1.988976	-3.484281
H	2.587382	-1.860971	-2.163013
C	3.201833	3.195702	0.173943
H	3.125239	2.659738	1.129713
C	1.769837	3.578103	-0.229199
H	1.304713	4.188919	0.564540
H	1.147611	2.683809	-0.383579
H	1.764472	4.172993	-1.160198
C	4.036365	4.462323	0.410406
H	3.604268	5.049156	1.239817
H	4.039934	5.112336	-0.481968
H	5.084995	4.236739	0.661948

3-As (*singlet*)

E(scf) = -12080.0827124 a.u.

B	1.395098	0.574765	0.618405
Fe	0.000264	-0.967326	0.000055
As	1.646915	-2.806578	0.280361

H	1.358328	-1.418767	3.357671
C	4.133236	0.892692	-0.634643
C	4.561619	0.032386	-1.672512
C	4.746421	0.573628	-2.948865
H	5.084124	-0.075596	-3.761702
C	4.481465	1.910390	-3.214588
H	4.629742	2.311829	-4.221134
C	3.997360	2.726401	-2.200455
H	3.750885	3.768131	-2.424818
C	3.807553	2.243411	-0.902445
C	4.730802	-1.474325	-1.508972
H	4.534244	-1.726269	-0.456089
C	6.135130	-1.965788	-1.890692
H	6.245493	-3.033413	-1.631960
H	6.936549	-1.406785	-1.384254
H	6.303613	-1.873918	-2.978305
C	3.694356	-2.237189	-2.347438
H	3.767860	-3.320766	-2.148874
H	3.856293	-2.080692	-3.428877
H	2.666496	-1.921113	-2.114517
C	3.194903	3.188448	0.124636
H	3.155552	2.666227	1.089412
C	1.746636	3.539338	-0.244165
H	1.292732	4.160489	0.548099
H	1.133007	2.633748	-0.360792
H	1.700958	4.111066	-1.188592
C	4.013186	4.473550	0.311890
H	3.596812	5.069189	1.143281
H	3.980128	5.106514	-0.592320
H	5.072028	4.268366	0.536365

3-As (*triplet*)

E(scf) = -12080.0608500 a.u.

B	1.393081	0.651700	0.615116
Fe	-0.001180	-0.980069	-0.000831
As	1.634175	-2.852195	0.287743

B	-1.395006	0.574441	-0.618383	B	-1.392684	0.653726	-0.615589
As	-0.283420	-2.811498	1.652812	As	-0.301048	-3.027531	1.675752
C	-0.123733	0.557792	-1.463857	C	-0.120572	0.732228	-1.475211
H	-0.138639	0.401999	-2.547406	H	-0.142413	0.651431	-2.567328
C	-1.162003	0.562088	0.888088	C	-1.154353	0.693802	0.910632
H	-1.984969	0.413751	1.591299	H	-1.983105	0.623880	1.618346
As	0.282256	-2.811816	-1.652577	As	0.302028	-3.022792	-1.677826
C	0.123822	0.557879	1.463884	C	0.121126	0.729011	1.474771
H	0.138720	0.402155	2.547442	H	0.142943	0.645151	2.566646
As	-1.648076	-2.805227	-0.280117	As	-1.633513	-2.854447	-0.289289
C	1.162118	0.562305	-0.888077	C	1.154730	0.695211	-0.911113
H	1.985102	0.414214	-1.591312	H	1.983473	0.626451	-1.619008
C	2.827600	0.517814	1.299691	C	2.815328	0.579287	1.293380
N	3.970794	0.840477	0.722717	N	3.973083	0.867956	0.715849
C	5.177589	0.836665	1.632795	C	5.177582	0.816460	1.623305
C	4.613225	0.118194	2.858518	C	4.590037	0.105671	2.843208
H	5.023479	0.523706	3.797029	H	5.020345	0.482772	3.784658
H	4.886871	-0.948755	2.811750	H	4.821605	-0.970457	2.781437
C	3.081504	0.248805	2.785071	C	3.063751	0.296517	2.779038
C	5.640829	2.267057	1.927027	C	5.690129	2.228132	1.929552
H	6.504957	2.224887	2.610497	H	6.555819	2.151846	2.608110
H	4.864148	2.880172	2.405077	H	4.936577	2.862206	2.416964
H	5.966265	2.769002	1.001830	H	6.026846	2.728503	1.007272
C	6.360447	0.076360	1.043342	C	6.335588	0.022124	1.027824
H	7.173931	0.072899	1.788469	H	7.147832	-0.015948	1.773394
H	6.739560	0.562624	0.130529	H	6.732257	0.504565	0.120411
H	6.106194	-0.969556	0.820015	H	6.047285	-1.012560	0.793433
C	2.589179	1.467963	3.597007	C	2.629260	1.527841	3.605599
H	2.858280	1.321776	4.657649	H	2.901974	1.362215	4.662515
H	1.498594	1.584994	3.529109	H	1.544073	1.691921	3.552113
H	3.046407	2.411696	3.259563	H	3.121573	2.454889	3.272170
C	2.435113	-1.026409	3.336495	C	2.369540	-0.955538	3.325634
H	2.776584	-1.173880	4.376854	H	2.688699	-1.110418	4.372041
H	2.737916	-1.908121	2.748002	H	2.656005	-1.847692	2.745300
H	1.337422	-0.984975	3.335639	H	1.273187	-0.885746	3.303714
C	4.114790	1.366852	-0.619508	C	4.124588	1.414268	-0.615015

C	4.566125	0.527625	-1.661984	C	4.549135	0.582975	-1.674703
C	4.796213	1.109308	-2.913035	C	4.779939	1.178648	-2.919075
H	5.149789	0.480993	-3.734871	H	5.113762	0.555995	-3.753449
C	4.557153	2.456716	-3.144473	C	4.563866	2.533729	-3.127937
H	4.742296	2.886440	-4.132925	H	4.748245	2.974961	-4.111465
C	4.049853	3.247787	-2.122502	C	4.079568	3.318637	-2.089824
H	3.820321	4.297956	-2.322545	H	3.867375	4.375621	-2.272618
C	3.811586	2.728437	-0.847202	C	3.843341	2.784624	-0.820253
C	4.712688	-0.986592	-1.551388	C	4.658553	-0.935442	-1.586891
H	4.481953	-1.284384	-0.516823	H	4.435474	-1.241229	-0.553238
C	6.118662	-1.485525	-1.919474	C	6.046761	-1.463631	-1.979357
H	6.203527	-2.562598	-1.693772	H	6.107804	-2.545576	-1.769926
H	6.921924	-0.959212	-1.381931	H	6.868427	-0.964659	-1.443224
H	6.309399	-1.363427	-3.000250	H	6.227967	-1.330447	-3.060512
C	3.689672	-1.697890	-2.449776	C	3.603892	-1.607186	-2.479012
H	3.742492	-2.788732	-2.290773	H	3.643382	-2.702612	-2.347443
H	3.888746	-1.500950	-3.518332	H	3.784013	-1.389219	-3.546940
H	2.659410	-1.380485	-2.231975	H	2.582735	-1.281527	-2.230939
C	3.169174	3.650359	0.183105	C	3.226932	3.701551	0.230672
H	3.097868	3.111301	1.137113	H	3.157127	3.148690	1.176538
C	1.733168	4.008324	-0.225303	C	1.792370	4.092289	-0.154820
H	1.253604	4.608355	0.567953	H	1.334742	4.694289	0.649989
H	1.128112	3.103401	-0.385097	H	1.165189	3.202618	-0.315787
H	1.721336	4.605524	-1.154841	H	1.779767	4.697901	-1.078773
C	3.980342	4.930954	0.425792	C	4.065589	4.961852	0.485164
H	3.537356	5.505233	1.258262	H	3.643680	5.530922	1.332052
H	3.971317	5.585926	-0.462876	H	4.060998	5.631753	-0.392366
H	5.033115	4.724024	0.675890	H	5.116312	4.728037	0.720283
N	-3.970569	0.841060	-0.722789	N	-3.972939	0.868089	-0.715167
C	-2.827512	0.517762	-1.299661	C	-2.814996	0.581167	-1.293358
C	-5.177266	0.837894	-1.632934	C	-5.177429	0.816889	-1.622543
C	-4.613223	0.119013	-2.858560	C	-4.589666	0.107685	-2.843259
H	-5.023308	0.524516	-3.797143	H	-5.020446	0.485443	-3.784225
H	-4.887305	-0.947814	-2.811539	H	-4.820490	-0.968666	-2.782513
C	-3.081447	0.248850	-2.785052	C	-3.063506	0.299512	-2.779174
C	-5.639642	2.268566	-1.927265	C	-5.690590	2.228665	-1.927331

H	-6.503522	2.226932	-2.611082	H	-6.556447	2.152678	-2.605712
H	-4.862469	2.881340	-2.404917	H	-4.937391	2.863452	-2.414388
H	-5.965170	2.770580	-1.002128	H	-6.027228	2.728039	-1.004484
C	-6.360674	0.078387	-1.043530	C	-6.335053	0.021379	-1.027877
H	-7.174135	0.075462	-1.788695	H	-7.147153	-0.016696	-1.773607
H	-6.739484	0.564971	-0.130766	H	-6.732188	0.502920	-0.120192
H	-6.107178	-0.967692	-0.820120	H	-6.046094	-1.013279	-0.794199
C	-2.588248	1.467685	-3.596919	C	-2.629838	1.531689	-3.604894
H	-2.857775	1.321934	-4.657513	H	-2.902589	1.366683	-4.661896
H	-1.497520	1.583588	-3.529301	H	-1.544716	1.696264	-3.551465
H	-3.044370	2.411846	-3.259210	H	-3.122553	2.458248	-3.270697
C	-2.435574	-1.026627	-3.336459	C	-2.368394	-0.951646	-3.326625
H	-2.776563	-1.173619	-4.377046	H	-2.688085	-1.106598	-4.372857
H	-2.739202	-1.908340	-2.748400	H	-2.653497	-1.844272	-2.746323
H	-1.337853	-0.985825	-3.334997	H	-1.272105	-0.880597	-3.305366
C	-4.114350	1.367527	0.619431	C	-4.124714	1.413216	0.616160
C	-4.566201	0.528503	1.661823	C	-4.548917	0.580897	1.675183
C	-4.795937	1.110219	2.912920	C	-4.780385	1.175577	2.919918
H	-5.149907	0.482058	3.734705	H	-5.113906	0.552128	3.753815
C	-4.556012	2.457465	3.144456	C	-4.565224	2.530636	3.129811
H	-4.740859	2.887225	4.132947	H	-4.750087	2.971023	4.113626
C	-4.048245	3.248302	2.122535	C	-4.081201	3.316590	2.092359
H	-3.818045	4.298303	2.322672	H	-3.869775	4.373594	2.275909
C	-3.810343	2.728921	0.847176	C	-3.844449	2.783630	0.822445
C	-4.713657	-0.985626	1.551060	C	-4.657296	-0.937574	1.586385
H	-4.483022	-1.283413	0.516467	H	-4.433641	-1.242548	0.552592
C	-6.119968	-1.483789	1.918895	C	-6.045315	-1.466945	1.977964
H	-6.205405	-2.560809	1.693146	H	-6.105551	-2.548785	1.767768
H	-6.922836	-0.957021	1.381216	H	-6.867114	-0.968187	1.441831
H	-6.310847	-1.361625	2.999633	H	-6.227043	-1.334617	3.059129
C	-3.691120	-1.697671	2.449431	C	-3.602624	-1.609254	2.478556
H	-3.744319	-2.788437	2.290060	H	-3.641861	-2.704663	2.346742
H	-3.890377	-1.501009	3.518004	H	-3.783032	-1.391575	3.546494
H	-2.660668	-1.380612	2.232011	H	-2.581485	-1.283411	2.230727
C	-3.167397	3.650518	-0.183107	C	-3.228585	3.701779	-0.227741
H	-3.096225	3.111350	-1.137070	H	-3.158555	3.149789	-1.174088

C	-1.731270	4.007879	0.225443
H	-1.251303	4.607469	-0.567900
H	-1.126690	3.102693	0.385570
H	-1.719283	4.605322	1.154822
C	-3.977978	4.931431	-0.425996
H	-3.534466	5.505553	-1.258293
H	-3.969008	5.586354	0.462705
H	-5.030739	4.724937	-0.676476

2-Ru (*singlet*)

E(scf) = -2195.12972342 a.u.

B	-1.420000	0.039238	-0.602151
Ru	-0.000092	-1.700393	-0.000024
O	0.371977	-3.866360	-2.067734
O	-0.371458	-3.866809	2.067332
B	1.419914	0.039085	0.602087
C	-0.134622	0.061341	-1.457104
H	-0.162174	-0.062071	-2.545842
C	0.134532	0.061298	1.457036
H	0.162062	-0.062184	2.545774
C	1.163297	0.106981	-0.911050
H	1.973948	0.044508	-1.642414
C	-1.163382	0.107085	0.910982
H	-1.974048	0.044631	1.642347
C	0.233770	-3.038213	-1.268792
C	-0.233509	-3.038452	1.268561
C	-2.832705	-0.065728	-1.276271
N	-3.999409	0.229223	-0.711046
C	-5.196914	0.154692	-1.619864
C	-4.612290	-0.638093	-2.789887
H	-5.041186	-0.325510	-3.755386
H	-4.848438	-1.706960	-2.654805
C	-3.082937	-0.448565	-2.737606
C	-5.663664	1.563214	-2.008096
H	-6.510752	1.481523	-2.709255
H	-4.878012	2.156877	-2.495700

C	-1.794204	4.092871	0.158067
H	-1.336813	4.695630	-0.646308
H	-1.166632	3.203354	0.318423
H	-1.781917	4.697840	1.082449
C	-4.067893	4.961837	-0.481206
H	-3.646262	5.531832	-1.327612
H	-4.063754	5.631065	0.396838
H	-5.118469	4.727611	-0.716600

(DBB)RuP₄ (*singlet*)

E(scf) = -3333.74801058 a.u.

B	-1.400133	0.290561	-0.614766
Ru	-0.000134	-1.387092	0.000054
P	-1.521045	-3.338538	-0.219884
P	0.221025	-3.324383	-1.522942
B	1.400151	0.290423	0.614677
C	-0.133182	0.291911	-1.472996
H	-0.154771	0.152968	-2.559268
P	1.520741	-3.338552	0.220149
C	-1.163543	0.279962	0.902371
H	-1.981051	0.152296	1.615073
C	0.133190	0.292068	1.472900
H	0.154747	0.153243	2.559187
P	-0.221326	-3.324261	1.523208
C	1.163546	0.279645	-0.902463
H	1.981023	0.151726	-1.615149
N	-3.979287	0.469066	-0.709283
C	-2.832334	0.176019	-1.289614
C	-5.192241	0.407155	-1.607324
C	-4.619045	-0.343640	-2.810042
H	-5.048191	0.014093	-3.759390
H	-4.864200	-1.414602	-2.719023
C	-3.089011	-0.169655	-2.756478
C	-5.678336	1.820613	-1.944974
H	-6.541359	1.746173	-2.627039
H	-4.910083	2.431824	-2.439219

H	-6.012961	2.110768	-1.117958	H	-6.011183	2.343754	-1.034185
C	-6.391718	-0.564281	-0.996582	C	-6.359680	-0.350086	-0.983044
H	-7.216042	-0.565212	-1.729623	H	-7.185203	-0.373323	-1.714369
H	-6.746470	-0.048333	-0.089539	H	-6.727437	0.150172	-0.072847
H	-6.166861	-1.611215	-0.749448	H	-6.096059	-1.389915	-0.743988
C	-2.641866	0.705633	-3.663306	C	-2.638025	1.013412	-3.641077
H	-2.928569	0.465402	-4.702235	H	-2.919075	0.803276	-4.687704
H	-1.553971	0.856577	-3.632537	H	-1.550036	1.160610	-3.599418
H	-3.115138	1.663404	-3.394302	H	-3.113460	1.963110	-3.347329
C	-2.390797	-1.750024	-3.164120	C	-2.408230	-1.461048	-3.225265
H	-2.770713	-2.049963	-4.157876	H	-2.755305	-1.694569	-4.247880
H	-2.596398	-2.560096	-2.446247	H	-2.675113	-2.304385	-2.566824
H	-1.299858	-1.644042	-3.227654	H	-1.312491	-1.386093	-3.235774
C	-4.142983	0.765623	0.619423	C	-4.120066	1.001720	0.628739
C	-4.527120	-0.095849	1.669234	C	-4.515591	0.146792	1.680403
C	-4.712329	0.461277	2.938090	C	-4.728182	0.720469	2.937918
H	-5.008484	-0.185977	3.768153	H	-5.037919	0.081388	3.769139
C	-4.497991	1.813244	3.172825	C	-4.523762	2.075182	3.162754
H	-4.646400	2.227182	4.174216	H	-4.694115	2.499036	4.156404
C	-4.063308	2.631037	2.137603	C	-4.067946	2.882230	2.128695
H	-3.854986	3.685214	2.340757	H	-3.862959	3.938232	2.324901
C	-3.865665	2.131762	0.847086	C	-3.847166	2.370303	0.847079
C	-4.647265	-1.608758	1.523817	C	-4.618979	-1.371028	1.565224
H	-4.519516	-1.861896	0.461450	H	-4.427905	-1.656577	0.519729
C	-6.006968	-2.141885	1.997863	C	-5.995631	-1.906598	1.988414
H	-6.105009	-3.210121	1.737415	H	-6.070061	-2.980706	1.745842
H	-6.856434	-1.600235	1.551661	H	-6.832404	-1.385329	1.497935
H	-6.102800	-2.065475	3.095241	H	-6.138442	-1.808755	3.079041
C	-3.517326	-2.326278	2.275068	C	-3.535183	-2.062266	2.406266
H	-3.607400	-3.418418	2.144812	H	-3.605838	-3.156386	2.280299
H	-3.553161	-2.109566	3.357813	H	-3.654509	-1.835768	3.480887
H	-2.533486	-2.029122	1.886354	H	-2.523810	-1.759186	2.098060
C	-3.287420	3.073548	-0.203603	C	-3.248611	3.304784	-0.199165
H	-3.224179	2.531562	-1.157069	H	-3.178965	2.762999	-1.152336
C	-1.854197	3.492744	0.158356	C	-1.815778	3.707524	0.182266
H	-1.429889	4.119975	-0.645597	H	-1.369243	4.320807	-0.620272

H	-1.202757	2.615140	0.288053	H	-1.179483	2.822229	0.332418
H	-1.835203	4.083079	1.091991	H	-1.805355	4.306396	1.110550
C	-4.160179	4.316808	-0.424583	C	-4.103260	4.558012	-0.434216
H	-3.763584	4.913311	-1.264822	H	-3.688342	5.146281	-1.271326
H	-4.162828	4.968430	0.466783	H	-4.107924	5.213032	0.454467
H	-5.207372	4.060452	-0.652464	H	-5.150794	4.314798	-0.674135
N	3.999373	0.229358	0.711103	N	3.979329	0.468952	0.709228
C	2.832662	-0.065642	1.276271	C	2.832366	0.175947	1.289556
C	5.196825	0.155036	1.620055	C	5.192262	0.406999	1.607310
C	4.612167	-0.637518	2.790231	C	4.619017	-0.343875	2.809941
H	5.040799	-0.324421	3.755680	H	5.048341	0.013624	3.759296
H	4.848640	-1.706371	2.655661	H	4.863865	-1.414893	2.718752
C	3.082781	-0.448301	2.737660	C	3.089049	-0.169358	2.756514
C	5.663504	1.563639	2.008043	C	5.678291	1.820436	1.945131
H	6.510499	1.482093	2.709326	H	6.541322	1.745957	2.627182
H	4.877765	2.157364	2.495456	H	4.909998	2.431533	2.439461
H	6.012935	2.111020	1.117858	H	6.011091	2.343712	1.034401
C	6.391650	-0.564052	0.996989	C	6.359731	-0.350165	0.983000
H	7.215819	-0.565111	1.730197	H	7.185236	-0.373472	1.714343
H	6.746645	-0.048094	0.090047	H	6.727506	0.150199	0.072871
H	6.166710	-1.610948	0.749775	H	6.096123	-1.389970	0.743817
C	2.641437	0.705910	3.663216	C	2.638730	1.014166	3.640833
H	2.927976	0.465723	4.702199	H	2.919902	0.804236	4.687468
H	1.553555	0.856839	3.632250	H	1.550801	1.161815	3.599354
H	3.114764	1.663667	3.394243	H	3.114515	1.963571	3.346688
C	2.390740	-1.749841	3.164099	C	2.407805	-1.460337	3.225771
H	2.770658	-2.049825	4.157844	H	2.754808	-1.693637	4.248462
H	2.596439	-2.559853	2.446181	H	2.674348	-2.304003	2.567612
H	1.299795	-1.643932	3.227598	H	1.312093	-1.384950	3.236266
C	4.143011	0.765687	-0.619401	C	4.120180	1.001541	-0.628818
C	4.527453	-0.095785	-1.669101	C	4.515732	0.146551	-1.680420
C	4.712697	0.461291	-2.937974	C	4.728389	0.720150	-2.937958
H	5.009103	-0.185946	-3.767962	H	5.038132	0.081013	-3.769135
C	4.498123	1.813198	-3.172845	C	4.524021	2.074855	-3.162875
H	4.646554	2.227076	-4.174258	H	4.694423	2.498648	-4.156542
C	4.063178	2.630981	-2.137734	C	4.068189	2.881973	-2.128880

H	3.854670	3.685102	-2.340987	H	3.863253	3.937974	-2.325152
C	3.865497	2.131770	-0.847197	C	3.847330	2.370123	-0.847246
C	4.647878	-1.608664	-1.523561	C	4.619037	-1.371265	-1.565171
H	4.520117	-1.861701	-0.461166	H	4.427965	-1.656738	-0.519655
C	6.007685	-2.141650	-1.997485	C	5.995651	-1.906928	-1.988374
H	6.105716	-3.209923	-1.737181	H	6.070057	-2.981016	-1.745705
H	6.857090	-1.600053	-1.551113	H	6.832468	-1.385641	-1.497989
H	6.103641	-2.065082	-3.094839	H	6.138414	-1.809194	-3.079017
C	3.518111	-2.326531	-2.274731	C	3.535186	-2.062465	-2.406172
H	3.608437	-3.418631	-2.144352	H	3.605592	-3.156572	-2.279955
H	3.553845	-2.109941	-3.357504	H	3.654685	-1.836225	-3.480827
H	2.534223	-2.029590	-1.886008	H	2.523847	-1.759074	-2.098163
C	3.287008	3.073596	0.203342	C	3.248785	3.304703	0.198911
H	3.223730	2.531710	1.156859	H	3.179207	2.763035	1.152154
C	1.853783	3.492614	-0.158810	C	1.815914	3.707342	-0.182490
H	1.429340	4.119897	0.645030	H	1.369402	4.320696	0.620006
H	1.202411	2.614961	-0.288478	H	1.179642	2.822007	-0.332508
H	1.834849	4.082860	-1.092506	H	1.805415	4.306114	-1.110837
C	4.159564	4.317016	0.424299	C	4.103389	4.558002	0.433736
H	3.762761	4.913545	1.264422	H	3.688484	5.146382	1.270774
H	4.162225	4.968544	-0.467136	H	4.107975	5.212880	-0.455054
H	5.206776	4.060878	0.652350	H	5.150949	4.314880	0.673636

(DBB)RuAs$_4$ (*singlet*)

E(scf) = -10911.4789200 a.u.

B	-1.399535	0.714119	-0.617281
Ru	-0.000044	-0.966726	-0.000025
As	-1.663983	-2.938719	-0.255211
B	1.399507	0.714067	0.617339
As	0.256431	-2.921651	-1.663837
C	0.130150	0.711093	1.473809
H	0.150210	0.576042	2.560637
C	1.165897	0.697349	-0.900464
H	1.986098	0.569881	-1.610364
As	-0.256431	-2.921612	1.663858
C	-0.130180	0.711206	-1.473759

2-Os (*singlet*)

E(scf) = -2190.95334555 a.u.

B	1.418856	-0.203705	-0.606657
Os	-0.000007	1.546019	0.000001
O	-0.466518	3.731087	-2.055944
O	0.466298	3.731295	2.055772
B	-1.418968	-0.203567	0.606753
C	0.132614	-0.259889	-1.461946
H	0.157726	-0.146283	-2.551661
C	-0.132730	-0.259811	1.462047
H	-0.157831	-0.146156	2.551756
C	-1.161575	-0.299563	-0.909170
H	-1.976151	-0.241291	-1.635837

H	-0.150244	0.576252	-2.560600	C	1.161456	-0.299601	0.909275
As	1.663982	-2.938638	0.255235	H	1.976032	-0.241379	1.635943
C	-1.165928	0.697324	0.900521	C	-0.297308	2.898425	-1.263832
H	-1.986135	0.569842	1.610416	C	0.297277	2.898515	1.263744
C	-2.832545	0.629501	-1.295392	C	2.826123	-0.041810	-1.275226
N	-3.977688	0.934871	-0.714570	N	4.000618	-0.301880	-0.708794
C	-5.189526	0.897964	-1.616088	C	5.197852	-0.160144	-1.609559
C	-4.620942	0.165859	-2.832198	C	4.585453	0.622345	-2.772358
H	-5.044635	0.546268	-3.775207	H	5.031354	0.339974	-3.739502
H	-4.876007	-0.904452	-2.762994	H	4.775937	1.698310	-2.622933
C	-3.090403	0.324263	-2.772103	C	3.065376	0.369598	-2.730719
C	-5.670064	2.318689	-1.930053	C	5.731267	-1.540014	-2.014175
H	-6.534891	2.257966	-2.611217	H	6.582161	-1.409262	-2.703207
H	-4.900777	2.934029	-2.417241	H	4.978560	-2.159421	-2.521485
H	-5.998968	2.829583	-1.010896	H	6.093774	-2.086908	-1.128728
C	-6.360921	0.134444	-1.007405	C	6.352779	0.606138	-0.969514
H	-7.181542	0.118066	-1.744465	H	7.173612	0.672783	-1.703532
H	-6.734887	0.626476	-0.095452	H	6.738176	0.085870	-0.077761
H	-6.098020	-0.907591	-0.776526	H	6.069110	1.631559	-0.693827
C	-2.626173	1.530465	-3.618698	C	2.676949	-0.793632	-3.668937
H	-2.901747	1.353172	-4.672853	H	2.950359	-0.528070	-4.705239
H	-1.537368	1.668283	-3.564523	H	1.597051	-0.994754	-3.639231
H	-3.096924	2.474431	-3.300764	H	3.194600	-1.731683	-3.412876
C	-2.422579	-0.954652	-3.288657	C	2.324301	1.644343	-3.155037
H	-2.763733	-1.140005	-4.323010	H	2.701036	1.964906	-4.143530
H	-2.707560	-1.822155	-2.670963	H	2.488888	2.456626	-2.429592
H	-1.326111	-0.893071	-3.287954	H	1.239161	1.494488	-3.230249
C	-4.117921	1.470002	0.623502	C	4.158070	-0.879534	0.603305
C	-4.541503	0.628211	1.675020	C	4.521395	-0.049728	1.685961
C	-4.761621	1.211936	2.926788	C	4.731374	-0.652219	2.930221
H	-5.093779	0.582320	3.756553	H	5.014664	-0.030205	3.783776
C	-4.538001	2.563821	3.148562	C	4.555837	-2.017564	3.112645
H	-4.714853	2.995327	4.137760	H	4.722583	-2.466526	4.095898
C	-4.055606	3.357593	2.116481	C	4.134789	-2.804842	2.048534
H	-3.836627	4.411328	2.309579	H	3.955016	-3.871248	2.211005
C	-3.827595	2.835795	0.840244	C	3.917362	-2.260704	0.779674

C	-4.667800	-0.888371	1.570157	C	4.587555	1.471653	1.607622
H	-4.461275	-1.185232	0.530491	H	4.419586	1.768736	0.562055
C	-6.058347	-1.402834	1.973490	C	5.938106	2.033553	2.075430
H	-6.140915	-2.478510	1.740595	H	5.988069	3.116439	1.867162
H	-6.880125	-0.877718	1.462755	H	6.797626	1.549801	1.585228
H	-6.219682	-1.291856	3.060255	H	6.062918	1.906615	3.165222
C	-3.610590	-1.585684	2.439414	C	3.457408	2.112122	2.425486
H	-3.687935	-2.680005	2.317686	H	3.482774	3.209708	2.317715
H	-3.753245	-1.353566	3.509872	H	3.554305	1.873473	3.499805
H	-2.589691	-1.290759	2.154897	H	2.473375	1.773527	2.075114
C	-3.205896	3.758559	-0.202431	C	3.361070	-3.177386	-0.304652
H	-3.136570	3.213921	-1.153656	H	3.283230	-2.602750	-1.237462
C	-1.770384	4.137611	0.190899	C	1.939249	-3.646585	0.041677
H	-1.304542	4.737670	-0.610574	H	1.533845	-4.260167	-0.782269
H	-1.152334	3.241639	0.352466	H	1.262409	-2.792589	0.197240
H	-1.758216	4.741676	1.115885	H	1.935899	-4.266048	0.956363
C	-4.036941	5.025759	-0.447249	C	4.265130	-4.388720	-0.572480
H	-3.606232	5.603240	-1.283918	H	3.883906	-4.962081	-1.435633
H	-4.035086	5.684412	0.438738	H	4.284489	-5.074305	0.292819
H	-5.087200	4.800703	-0.692644	H	5.305249	-4.096526	-0.789273
N	3.977678	0.934798	0.714564	N	-4.000679	-0.301872	0.708762
C	2.832543	0.629447	1.295419	C	-2.826264	-0.041660	1.275267
C	5.189530	0.897879	1.616064	C	-5.198019	-0.160118	1.609382
C	4.620983	0.165723	2.832156	C	-4.585755	0.622399	2.772234
H	5.044710	0.546101	3.775163	H	-5.031749	0.340026	3.739334
H	4.876026	-0.904589	2.762908	H	-4.776258	1.698357	2.622774
C	3.090454	0.324196	2.772128	C	-3.065662	0.369705	2.730748
C	5.670048	2.318593	1.930115	C	-5.731474	-1.539981	2.013970
H	6.534951	2.257821	2.611179	H	-6.582462	-1.409214	2.702884
H	4.900793	2.933845	2.417464	H	-4.978831	-2.159366	2.521401
H	5.998827	2.829616	1.010981	H	-6.093858	-2.086903	1.128492
C	6.360920	0.134391	1.007337	C	-6.352865	0.606163	0.969187
H	7.181564	0.117993	1.744370	H	-7.173799	0.672766	1.703096
H	6.734850	0.626478	0.095400	H	-6.738124	0.085928	0.077355
H	6.098023	-0.907637	0.776421	H	-6.069172	1.631599	0.693588
C	2.626369	1.530438	3.618740	C	-2.677297	-0.793536	3.668983

H	2.901866	1.353058	4.672901	H	-2.950872	-0.528034	4.705256
H	1.537592	1.668429	3.564520	H	-1.597382	-0.994587	3.639426
H	3.097292	2.474344	3.300881	H	-3.194844	-1.731615	3.412809
C	2.422601	-0.954674	3.288762	C	-2.324685	1.644472	3.155170
H	2.763791	-1.140001	4.323107	H	-2.701577	1.965023	4.143607
H	2.707511	-1.822216	2.671090	H	-2.489193	2.456753	2.429705
H	1.326136	-0.893030	3.288115	H	-1.239554	1.494653	3.230544
C	4.117886	1.469952	-0.623508	C	-4.157969	-0.879656	-0.603295
C	4.541444	0.628182	-1.675056	C	-4.521248	-0.049958	-1.686039
C	4.761592	1.211938	-2.926804	C	-4.731073	-0.652554	-2.930274
H	5.093735	0.582339	-3.756588	H	-5.014336	-0.030625	-3.783899
C	4.538024	2.563835	-3.148541	C	-4.555424	-2.017902	-3.112579
H	4.714903	2.995364	-4.137724	H	-4.722054	-2.466951	-4.095812
C	4.055654	3.357592	-2.116438	C	-4.134404	-2.805067	-2.048372
H	3.836745	4.411349	-2.309494	H	-3.954518	-3.871467	-2.210754
C	3.827610	2.835766	-0.840219	C	-3.917135	-2.260819	-0.779530
C	4.667739	-0.888404	-1.570255	C	-4.587520	1.471422	-1.607791
H	4.461296	-1.185305	-0.530584	H	-4.419738	1.768563	-0.562207
C	6.058238	-1.402866	-1.973739	C	-5.938031	2.033218	-2.075831
H	6.140757	-2.478575	-1.740984	H	-5.988096	3.116107	-1.867595
H	6.880070	-0.877866	-1.462982	H	-6.797596	1.549421	-1.585749
H	6.219526	-1.291752	-3.060498	H	-6.062667	1.906253	-3.165639
C	3.610470	-1.585673	-2.439470	C	-3.457272	2.111898	-2.425510
H	3.687802	-2.679998	-2.317783	H	-3.482693	3.209490	-2.317791
H	3.753061	-1.353533	-3.509930	H	-3.554001	1.873193	-3.499831
H	2.589592	-1.290753	-2.154876	H	-2.473281	1.773352	-2.074975
C	3.205969	3.758561	0.202466	C	-3.360811	-3.177353	0.304909
H	3.136595	3.213921	1.153684	H	-3.283028	-2.602603	1.237657
C	1.770483	4.137711	-0.190874	C	-1.938951	-3.646499	-0.041332
H	1.304641	4.737725	0.610634	H	-1.533538	-4.259982	0.782684
H	1.152401	3.241778	-0.352522	H	-1.262149	-2.792485	-0.196953
H	1.758361	4.741851	-1.115813	H	-1.935545	-4.266049	-0.955959
C	4.037084	5.025713	0.447277	C	-4.264783	-4.388729	0.572852
H	3.606412	5.603217	1.283949	H	-3.883542	-4.961947	1.436093
H	4.035244	5.684359	-0.438715	H	-4.284035	-5.074427	-0.292359
H	5.087336	4.800612	0.692653	H	-5.304940	-4.096612	0.789561

DBB

E(scf) = -1873.66165568 a.u.

B	-1.382205	0.596225	-0.515210
B	1.382161	-0.596227	-0.515026
C	-0.121892	1.460374	-0.548670
H	-0.151643	2.558952	-0.554611
C	0.121858	-1.460388	-0.548296
H	0.151612	-2.558964	-0.553967
C	1.155440	0.916510	-0.509411
H	1.969695	1.649095	-0.486165
C	-1.155475	-0.916509	-0.509202
H	-1.969726	-1.649094	-0.485752
C	-2.788073	1.285566	-0.437189
N	-3.951707	0.709996	-0.111527
C	-5.132359	1.629108	-0.006231
C	-4.598220	2.857408	-0.745633
H	-4.984546	3.796889	-0.318482
H	-4.928731	2.816013	-1.797480
C	-3.057520	2.775322	-0.689561
C	-5.477151	1.913738	1.461196
H	-6.312302	2.632876	1.504926
H	-4.632855	2.340522	2.021127
H	-5.799270	0.989798	1.966549
C	-6.390987	1.070871	-0.672644
H	-7.212493	1.793145	-0.529641
H	-6.699090	0.112117	-0.223277
H	-6.259908	0.929557	-1.754697
C	-2.509819	3.636107	0.466701
H	-2.805366	4.689536	0.312547
H	-1.414096	3.585641	0.524488
H	-2.904875	3.311054	1.442730
C	-2.477102	3.252558	-2.029667
H	-2.844584	4.272661	-2.246277
H	-2.797819	2.588780	-2.851130
H	-1.378670	3.267516	-2.025797

DBB^{2-}

E(scf) = -1873.59838090 a.u.

B	1.434596	-0.640319	-0.475266
B	-1.434588	0.640209	-0.475289
C	0.083955	-1.473777	-0.433368
H	0.107448	-2.577267	-0.354702
C	-0.083945	1.473667	-0.433398
H	-0.107434	2.577158	-0.354772
C	-1.162885	-0.922581	-0.396475
H	-1.991428	-1.641050	-0.287394
C	1.162893	0.922470	-0.396454
H	1.991436	1.640935	-0.287348
C	2.764196	-1.304407	-0.496080
N	4.081384	-0.666705	-0.350891
C	5.208476	-1.594717	-0.358207
C	4.576663	-2.834604	-1.005374
H	5.061975	-3.770699	-0.668316
H	4.722294	-2.766851	-2.098616
C	3.061085	-2.796870	-0.703444
C	5.769455	-1.910457	1.048114
H	6.597264	-2.642463	0.981764
H	4.999708	-2.327136	1.712682
H	6.162776	-0.990328	1.515313
C	6.405041	-1.102935	-1.191734
H	7.210548	-1.862043	-1.187588
H	6.817569	-0.167391	-0.773847
H	6.116934	-0.918855	-2.238895
C	2.726159	-3.657932	0.535072
H	2.994269	-4.726323	0.380184
H	1.647702	-3.593118	0.751769
H	3.257389	-3.303590	1.434157
C	2.329155	-3.402707	-1.915848
H	2.677851	-4.440027	-2.113172
H	2.516194	-2.786893	-2.812416
H	1.240539	-3.424644	-1.760925

C	-4.098576	-0.681268	0.214285	C	4.231310	0.619965	0.206474
C	-4.479292	-1.575306	-0.808794	C	4.635142	1.687811	-0.639102
C	-4.660557	-2.919202	-0.472986	C	4.877653	2.952899	-0.094110
H	-4.953086	-3.632647	-1.248544	H	5.192535	3.772383	-0.750686
C	-4.446124	-3.372549	0.823280	C	4.673353	3.202404	1.257480
H	-4.591117	-4.429206	1.065932	H	4.857084	4.199709	1.672621
C	-4.015291	-2.485816	1.801720	C	4.184959	2.181561	2.068479
H	-3.804260	-2.860711	2.807254	H	3.970788	2.392848	3.122093
C	-3.817988	-1.130138	1.521542	C	3.946505	0.897811	1.570666
C	-4.599279	-1.164954	-2.271908	C	4.665735	1.520596	-2.152432
H	-4.529420	-0.068925	-2.324977	H	4.517005	0.449690	-2.347245
C	-5.936958	-1.590450	-2.892669	C	5.986307	1.969466	-2.790379
H	-6.050957	-1.143258	-3.895784	H	5.995630	1.725640	-3.869611
H	-6.798710	-1.281655	-2.278030	H	6.858518	1.481557	-2.322994
H	-5.991745	-2.686270	-3.017072	H	6.126675	3.063662	-2.702070
C	-3.428219	-1.713521	-3.101743	C	3.482685	2.251237	-2.804686
H	-3.536421	-1.402642	-4.156307	H	3.485910	2.082506	-3.898660
H	-3.405652	-2.817898	-3.074685	H	3.538315	3.342600	-2.627207
H	-2.462069	-1.341018	-2.726677	H	2.525234	1.884199	-2.399497
C	-3.223708	-0.244850	2.610061	C	3.335779	-0.137738	2.505180
H	-3.173731	0.782766	2.225286	H	3.248815	-1.071580	1.935047
C	-1.780275	-0.659397	2.934914	C	1.904946	0.248709	2.904991
H	-1.352150	0.031290	3.683038	H	1.449557	-0.556713	3.510634
H	-1.145595	-0.633005	2.035042	H	1.277791	0.398318	2.010363
H	-1.744762	-1.679921	3.357572	H	1.892025	1.178025	3.507340
C	-4.070249	-0.236733	3.890447	C	4.188820	-0.383478	3.757860
H	-3.672073	0.505942	4.603955	H	3.770278	-1.224338	4.342265
H	-4.045225	-1.219563	4.393398	H	4.199840	0.502250	4.421260
H	-5.127191	0.009323	3.696307	H	5.235447	-0.628199	3.509369
N	3.951672	-0.710024	-0.111300	N	-4.081388	0.666678	-0.350964
C	2.788014	-1.285593	-0.436874	C	-2.764175	1.304328	-0.496144
C	5.132250	-1.629204	-0.005735	C	-5.208441	1.594733	-0.358276
C	4.598070	-2.857578	-0.744967	C	-4.576588	2.834575	-1.005486
H	4.984379	-3.797020	-0.317719	H	-5.061863	3.770697	-0.668452
H	4.928564	-2.816294	-1.796826	H	-4.722228	2.766796	-2.098725
C	3.057373	-2.775422	-0.688894	C	-3.061009	2.796798	-0.703555

C	5.476846	-1.913586	1.461788	C	-5.769385	1.910532	1.048044
H	6.311886	-2.632839	1.505763	H	-6.597137	2.642602	0.981684
H	4.632430	-2.340110	2.021728	H	-4.999605	2.327157	1.712608
H	5.799049	-0.989581	1.966967	H	-6.162776	0.990439	1.515252
C	6.391005	-1.071180	-0.672099	C	-6.405027	1.102945	-1.191769
H	7.212459	-1.793467	-0.528865	H	-7.210536	1.862052	-1.187629
H	6.699115	-0.112360	-0.222878	H	-6.817545	0.167411	-0.773850
H	6.260075	-0.930055	-1.754195	H	-6.116940	0.918842	-2.238933
C	2.509624	-3.635969	0.467517	C	-2.726069	3.657880	0.534943
H	2.805242	-4.689412	0.313614	H	-2.994157	4.726272	0.380027
H	1.413892	-3.585560	0.525192	H	-1.647616	3.593047	0.751646
H	2.904555	-3.310687	1.443516	H	-3.257316	3.303573	1.434032
C	2.476886	-3.252884	-2.028891	C	-2.329055	3.402574	-1.915977
H	2.844201	-4.273096	-2.245267	H	-2.677705	4.439906	-2.113323
H	2.797696	-2.589346	-2.850514	H	-2.516124	2.786746	-2.812528
H	1.378448	-3.267663	-2.024998	H	-1.240437	3.424468	-1.761056
C	4.098630	0.681321	0.214111	C	-4.231332	-0.619945	0.206512
C	4.479393	1.574997	-0.809264	C	-4.635220	-1.687844	-0.638965
C	4.660670	2.919004	-0.473921	C	-4.877726	-2.952890	-0.093870
H	4.953243	3.632174	-1.249716	H	-5.192654	-3.772416	-0.750371
C	4.446209	3.372802	0.822187	C	-4.673364	-3.202299	1.257728
H	4.591193	4.429546	1.064466	H	-4.857082	-4.199572	1.672950
C	4.015373	2.486410	1.800929	C	-4.184927	-2.181397	2.068629
H	3.804342	2.861645	2.806336	H	-3.970712	-2.392609	3.122249
C	3.818082	1.130628	1.521222	C	-3.946485	-0.897686	1.570713
C	4.599430	1.164105	-2.272223	C	-4.665898	-1.520737	-2.152306
H	4.529624	0.068052	-2.324883	H	-4.517133	-0.449853	-2.347206
C	5.937111	1.589447	-2.893103	C	-5.986537	-1.969584	-2.790128
H	6.051157	1.141939	-3.896072	H	-5.995903	-1.725880	-3.869388
H	6.798863	1.280892	-2.278340	H	-6.858681	-1.481550	-2.322749
H	5.991842	2.685232	-3.017854	H	-6.126987	-3.063758	-2.701681
C	3.428349	1.712267	-3.102296	C	-3.482924	-2.251480	-2.804581
H	3.536594	1.400972	-4.156735	H	-3.486193	-2.082810	-3.898564
H	3.405691	2.816652	-3.075688	H	-3.538602	-3.342831	-2.627041
H	2.462220	1.339857	-2.727085	H	-2.525433	-1.884476	-2.399457
C	3.223885	0.245697	2.610078	C	-3.335720	0.137928	2.505129

H	3.174098	-0.782089	2.225731
C	1.780370	0.660137	2.934707
H	1.352330	-0.030301	3.683111
H	1.145737	0.633262	2.034817
H	1.744658	1.680836	3.356926
C	4.070387	0.238266	3.890492
H	3.672368	-0.504230	4.604273
H	4.045116	1.221287	4.393059
H	5.127396	-0.007607	3.696472

DBB^{2+}

E(scf) = -1873.18739647 a.u.

B	-1.398763	-0.541342	-0.343619
B	1.398679	-0.540932	0.343943
C	-0.328187	-0.293192	-1.467647
H	-0.620138	-0.139933	-2.514028
C	0.328052	-0.292965	1.467983
H	0.619948	-0.139550	2.514351
C	0.984586	-0.314145	-1.147951
H	1.732431	-0.174859	-1.935689
C	-0.984712	-0.314585	1.148293
H	-1.732618	-0.175653	1.936021
C	-2.833891	-1.044307	-0.822969
N	-3.913827	-0.361980	-0.629335
C	-5.078582	-0.836024	-1.493229
C	-4.641442	-2.287699	-1.744967
H	-4.965232	-2.636129	-2.736944
H	-5.107514	-2.950062	-0.996991
C	-3.098747	-2.320970	-1.591459
C	-5.075968	0.005894	-2.774833
H	-5.844249	-0.389670	-3.458418
H	-4.107523	-0.024897	-3.298174
H	-5.327741	1.055546	-2.560682
C	-6.435432	-0.695673	-0.815624
H	-7.215644	-0.916053	-1.561716
H	-6.601306	0.333173	-0.455348

H	-3.248790	1.071732	1.934924
C	-1.904862	-0.248476	2.904898
H	-1.449449	0.557004	3.510447
H	-1.277759	-0.398171	2.010250
H	-1.891903	-1.177735	3.507332
C	-4.188705	0.383744	3.757833
H	-3.770157	1.224657	4.342158
H	-4.199666	-0.501937	4.421296
H	-5.235353	0.628417	3.509381

P_4 (T_d)

E(scf) = -1364.99463794 a.u.

P	0.779601	0.779601	0.779601
P	-0.779601	-0.779601	0.779601
P	-0.779601	0.779601	-0.779601
P	0.779601	-0.779601	-0.779601

P_4 (D_{2h})

E(scf) = -1364.90469811 a.u.

P	0.000000	1.018353	1.154950
P	0.000000	1.018353	-1.154950
P	0.000000	-1.018353	-1.154950
P	0.000000	-1.018353	1.154950

P_4^{2-} (D_{4h})

E(scf) = -1364.88234986 a.u.

P	0.000000	1.526668	0.000000
P	1.526668	0.000000	0.000000
P	0.000000	-1.526668	0.000000
P	-1.526668	0.000000	0.000000

P_4^{4-} (C_{2v})

E(scf) = -1363.91970777 a.u.

P	0.000000	1.715586	0.126023
P	1.715586	0.000000	-0.126023
P	0.000000	-1.715586	0.126023

H	-6.574717	-1.396105	0.018220
C	-2.357762	-2.396969	-2.937238
H	-2.671429	-3.314718	-3.461245
H	-1.266056	-2.453516	-2.797820
H	-2.583691	-1.542695	-3.595313
C	-2.638539	-3.483600	-0.688642
H	-2.918585	-4.442652	-1.156279
H	-3.111960	-3.440019	0.307388
H	-1.541138	-3.482472	-0.556745
C	-3.974737	0.736306	0.319798
C	-4.415957	0.411461	1.622322
C	-4.427149	1.440778	2.565111
H	-4.759722	1.231430	3.585025
C	-4.023349	2.730670	2.233347
H	-4.052182	3.521894	2.986862
C	-3.579827	3.011907	0.948541
H	-3.258033	4.027756	0.705417
C	-3.533222	2.022338	-0.040956
C	-4.846311	-0.988258	2.055874
H	-4.903594	-1.628798	1.163655
C	-6.238639	-0.971660	2.704384
H	-6.610418	-2.001947	2.833649
H	-6.970125	-0.413208	2.098088
H	-6.211572	-0.507699	3.704780
C	-3.828648	-1.652685	2.994985
H	-4.230546	-2.610680	3.365977
H	-3.612559	-1.019510	3.872935
H	-2.874900	-1.872440	2.485992
C	-2.987832	2.389957	-1.413534
H	-2.973243	1.483317	-2.039346
C	-1.542563	2.901230	-1.317117
H	-1.115167	3.037788	-2.325044
H	-0.894811	2.202637	-0.759886
H	-1.494336	3.875892	-0.802656
C	-3.877270	3.424769	-2.117366
H	-3.524928	3.597567	-3.148192
P	-1.715586	0.000000	-0.126023

H	-3.851440	4.393209	-1.589543
H	-4.931060	3.104805	-2.162449
N	3.913741	-0.361854	0.629464
C	2.833750	-1.043979	0.823404
C	5.078500	-0.835692	1.493416
C	4.641318	-2.287310	1.745457
H	4.965181	-2.635565	2.737468
H	5.107333	-2.949802	0.997558
C	3.098603	-2.320580	1.592043
C	5.075883	0.006562	2.774804
H	5.844099	-0.388858	3.458543
H	4.107404	-0.023998	3.298107
H	5.327735	1.056142	2.560363
C	6.435331	-0.695513	0.815737
H	7.215539	-0.915801	1.561864
H	6.601251	0.333262	0.455294
H	6.574578	-1.396079	-0.018006
C	2.357766	-2.396457	2.937912
H	2.671671	-3.314066	3.462015
H	1.266054	-2.453195	2.798639
H	2.583644	-1.542044	3.595834
C	2.638325	-3.483339	0.689421
H	2.918373	-4.442325	1.157193
H	3.111692	-3.439900	-0.306636
H	1.540914	-3.482199	0.557588
C	3.974852	0.736107	-0.319991
C	4.416079	0.410723	-1.622381
C	4.427616	1.439728	-2.565500
H	4.760257	1.229983	-3.585309
C	4.024151	2.729837	-2.234153
H	4.053285	3.520824	-2.987903
C	3.580613	3.011608	-0.949471
H	3.259073	4.027623	-0.706702
C	3.533670	2.022370	0.040341
C	4.846048	-0.989297	-2.055380
H	4.903903	-1.629310	-1.162816

C	6.237932	-0.973314	-2.704860
H	6.609502	-2.003734	-2.833650
H	6.969892	-0.414532	-2.099435
H	6.210263	-0.510069	-3.705573
C	3.827595	-1.654176	-2.993329
H	4.229218	-2.612276	-3.364352
H	3.610629	-1.021332	-3.871306
H	2.874349	-1.873805	-2.483353
C	2.988255	2.390566	1.412750
H	2.973768	1.484234	2.039017
C	1.542926	2.901614	1.316004
H	1.115412	3.038561	2.323831
H	0.895319	2.202700	0.759014
H	1.494626	3.876037	0.801098
C	3.877539	3.425800	2.116166
H	3.525139	3.598998	3.146905
H	3.851633	4.394021	1.587943
H	4.931349	3.105938	2.161425

As_4 (*T_d*)

E(scf) = -8942.73698852 a.u.

As	0.859206	0.859206	0.859206
As	-0.859206	-0.859206	0.859206
As	-0.859206	0.859206	-0.859206
As	0.859206	-0.859206	-0.859206

As_4 (*C_{2v}*)

E(scf) = -8942.65690338 a.u.

As	0.000000	1.313834	0.350691
As	1.822181	0.000000	-0.350691
As	-1.822181	0.000000	-0.350691
As	0.000000	-1.313834	0.350691

As_4^{2-} (*D_{4h}*)

E(scf) = -8942.62941198 a.u.

As	0.000000	1.676817	0.000000

$FeCO_2$ (*singlet*)

E(scf) = -1489.88135090 a.u.

O	2.021012	-1.150820	0.000016
O	-2.021009	-1.150822	-0.000016
Fe	0.000000	0.863760	0.000000
C	1.203174	-0.337054	-0.000035
C	-1.203177	-0.337051	0.000036

$FeCO_2$ (*triplet*)

E(scf) = -1489.93008734 a.u.

O	2.311499	-1.040482	0.000040
O	-2.311499	-1.040482	-0.000039
Fe	0.000000	0.772025	0.000000
C	1.440111	-0.285412	-0.000085
C	-1.440111	-0.285413	0.000083

$FeCO_2$ (*quintuplet*)

As	1.676817	0.000000	0.000000
As	-1.676817	0.000000	0.000000
As	0.000000	-1.676817	0.000000

As_4^{4-} (C_{2v})

E(scf) = -8941.76703504 a.u.

As	0.000000	1.857448	0.176675
As	1.857448	0.000000	-0.176675
As	-1.857448	0.000000	-0.176675
As	0.000000	-1.857448	0.176675

E(scf) = -1489.90524836 a.u.

O	2.732231	-0.727520	-0.000012
O	-2.732231	-0.727520	0.000012
Fe	0.000000	0.602253	0.000000
C	1.657655	-0.334856	-0.000001
C	-1.657655	-0.334856	0.000001

$RuCO_2$ (*singlet*)

E(scf) = -321.302809093 a.u.

Ru	0.000000	-0.691325	0.000005
O	2.052271	1.438355	-0.000002
O	-2.052371	1.438258	0.000050
C	1.243664	0.617052	0.000005
C	-1.243530	0.617179	-0.000109

$RuCO_2$ (*triplet*)

E(scf) = -321.299503309 a.u.

Ru	-0.000002	-0.651723	0.000010
O	2.237319	1.390487	0.000019
O	-2.237382	1.390430	0.000056
C	1.468876	0.535665	-0.000048
C	-1.468774	0.535748	-0.000124

$RuCO_2$ (*quintuplet*)

E(scf) = -321.213265018 a.u.

Ru	-0.000001	0.444836	0.000030
O	2.874891	-0.852825	0.000218
O	-2.874886	-0.852828	0.000219
C	1.790266	-0.493964	-0.000401
C	-1.790264	-0.493962	-0.000404

$OsCO_2$ (*singlet*)

E(scf) = -317.107295491 a.u.

Os	0.000000	0.474431	0.000000
O	2.082101	-1.638665	0.000342
O	-2.082113	-1.638654	-0.000341
C	1.267980	-0.819846	-0.000748
C	-1.267967	-0.819858	0.000746

$OsCO_2$ (*triplet*)

E(scf) = -317.093466881 a.u.

Os	0.000002	0.431707	0.000000
O	2.298475	-1.529320	0.000003
O	-2.298507	-1.529291	-0.000002
C	1.501925	-0.695067	-0.000006
C	-1.501914	-0.695078	0.000004

(DBB)Fe^0 (*singlet*)

E(scf) = -3137.19769495 a.u.

(DBB)Fe^0 (*triplet*)

E(scf) = -3137.24405218 a.u.

B	1.406851	-0.673237	1.084401
Fe	-0.001739	0.000917	-0.335476
B	-1.407283	0.673442	1.086821
C	-0.083888	1.454078	1.266534
H	-0.054320	2.549193	1.202686
C	-1.181384	-0.856760	1.240926
H	-1.997263	-1.578476	1.129885
C	0.083841	-1.454027	1.265645
H	0.054104	-2.549135	1.201549
C	1.181302	0.856837	1.239552
H	1.996988	1.578667	1.127759
C	2.557507	-1.315168	0.315558
N	3.714846	-0.708263	-0.111773
C	4.753609	-1.603618	-0.681539
C	3.935657	-2.866352	-0.956697
H	4.520591	-3.784509	-0.782836
H	3.634469	-2.872664	-2.017985
C	2.671592	-2.805726	-0.069004
C	5.893073	-1.874744	0.312551
H	6.603365	-2.594123	-0.130352
H	5.524640	-2.302151	1.257689
H	6.447930	-0.950786	0.537577
C	5.377684	-1.049037	-1.964984
H	6.142086	-1.755155	-2.331847
H	5.867965	-0.075805	-1.792227
H	4.627531	-0.928819	-2.760119
C	2.832909	-3.653553	1.205683
H	3.014997	-4.712316	0.945135
H	1.927415	-3.598022	1.830270
H	3.676725	-3.301330	1.821995
C	1.512371	-3.358383	-0.915169
H	1.785623	-4.362513	-1.290788
H	1.327702	-2.704749	-1.784127
H	0.571461	-3.452490	-0.358013
C	3.931748	0.699761	-0.036072
C	3.477994	1.504818	-1.106307

B	-1.421701	0.636650	-0.327709
Fe	-0.002466	0.026148	-1.965193
B	1.426000	-0.615122	-0.348417
C	0.123999	-1.468134	-0.499114
H	0.149251	-2.558680	-0.608577
C	1.173978	0.923301	-0.441522
H	1.996242	1.640526	-0.523631
C	-0.121047	1.491833	-0.471151
H	-0.147463	2.583971	-0.561102
C	-1.170276	-0.900636	-0.451014
H	-1.992695	-1.617298	-0.540301
C	-2.800075	1.305286	-0.194723
N	-3.973622	0.690920	0.130664
C	-5.124497	1.596822	0.399436
C	-4.632793	2.885199	-0.266371
H	-4.987569	3.785026	0.262533
H	-5.035120	2.932405	-1.292941
C	-3.089300	2.807444	-0.323599
C	-5.369860	1.774168	1.904882
H	-6.159517	2.528359	2.063612
H	-4.469705	2.108122	2.441120
H	-5.711132	0.828372	2.354292
C	-6.433911	1.101977	-0.221432
H	-7.244925	1.799136	0.050198
H	-6.706940	0.101212	0.154030
H	-6.384393	1.063241	-1.319297
C	-2.459235	3.607479	0.833211
H	-2.769796	4.666763	0.774046
H	-1.361111	3.565424	0.801641
H	-2.770418	3.214967	1.814689
C	-2.623110	3.379328	-1.673013
H	-3.006381	4.409532	-1.794701
H	-3.011013	2.767635	-2.506380
H	-1.528003	3.408834	-1.762369
C	-4.100945	-0.723749	0.287809
C	-4.502442	-1.488793	-0.828255

C	3.699621	2.881856	-1.052976	C	-4.654097	-2.868815	-0.671182
H	3.351830	3.519527	-1.871447	H	-4.968486	-3.478551	-1.523224
C	4.339751	3.462443	0.037031	C	-4.384751	-3.485768	0.545675
H	4.507973	4.542893	0.064259	H	-4.505954	-4.567738	0.652017
C	4.737492	2.667084	1.103905	C	-3.934335	-2.725470	1.617601
H	5.198819	3.137694	1.977063	H	-3.685985	-3.223962	2.559155
C	4.528906	1.283166	1.100343	C	-3.769447	-1.340659	1.511831
C	2.667517	0.935847	-2.263086	C	-4.688451	-0.878834	-2.211495
H	2.657986	-0.157797	-2.155502	H	-4.630494	0.213808	-2.108147
C	3.250359	1.289323	-3.636904	C	-6.048243	-1.225738	-2.830825
H	2.700285	0.762111	-4.436688	H	-6.200316	-0.656938	-3.765091
H	4.314521	1.013883	-3.712501	H	-6.880516	-0.991081	-2.146116
H	3.172647	2.371579	-3.841855	H	-6.116533	-2.298165	-3.085556
C	1.207880	1.401780	-2.188497	C	-3.547330	-1.286196	-3.155467
H	0.567362	0.841272	-2.886374	H	-3.694185	-0.827953	-4.150197
H	1.112025	2.469546	-2.455336	H	-3.509063	-2.382501	-3.288432
H	0.815262	1.402127	-1.141483	H	-2.573217	-0.956450	-2.756655
C	4.846413	0.496016	2.363595	C	-3.172595	-0.586914	2.692732
H	4.666101	-0.564930	2.149098	H	-3.149095	0.480089	2.433764
C	3.895178	0.878247	3.507781	C	-1.717961	-1.007662	2.951749
H	4.129531	0.281422	4.407246	H	-1.300224	-0.415953	3.785685
H	2.845478	0.688438	3.234112	H	-1.089133	-0.842992	2.062662
H	3.997399	1.945467	3.775256	H	-1.653550	-2.075021	3.230681
C	6.305567	0.660829	2.808798	C	-4.004130	-0.755948	3.971899
H	6.534097	-0.035443	3.634695	H	-3.605312	-0.110113	4.773926
H	6.500075	1.683168	3.178945	H	-3.966003	-1.797039	4.338908
H	7.017558	0.464688	1.989616	H	-5.065106	-0.496940	3.820883
N	-3.714183	0.707809	-0.112274	N	3.978264	-0.696788	0.109057
C	-2.556338	1.314719	0.315456	C	2.801664	-1.293805	-0.239192
C	-4.752331	1.603329	-0.682469	C	5.127607	-1.615571	0.334085
C	-3.933523	2.865234	-0.958731	C	4.622380	-2.880455	-0.367023
H	-4.518054	3.784004	-0.786693	H	4.976240	-3.798418	0.130292
H	-3.631505	2.869772	-2.019804	H	5.015555	-2.897037	-1.398146
C	-2.670101	2.805169	-0.070099	C	3.079099	-2.793448	-0.409670
C	-5.891623	1.876129	0.311449	C	5.392562	-1.841317	1.830304
H	-6.601228	2.596012	-0.131757	H	6.225997	-2.553875	1.952809

H	-5.522841	2.303547	1.256434
H	-6.447386	0.952811	0.536862
C	-5.377142	1.048311	-1.965412
H	-6.141401	1.754511	-2.332425
H	-5.867771	0.075385	-1.791877
H	-4.627382	0.927267	-2.760796
C	-2.832262	3.653880	1.203910
H	-3.014823	4.712391	0.942623
H	-1.926884	3.599256	1.828762
H	-3.675980	3.301632	1.820310
C	-1.510551	3.357734	-0.915846
H	-1.783733	4.361693	-1.292011
H	-1.325355	2.703783	-1.784451
H	-0.569906	3.452219	-0.358278
C	-3.931589	-0.699981	-0.035809
C	-3.478057	-1.505807	-1.105574
C	-3.699719	-2.882810	-1.051484
H	-3.352048	-3.520966	-1.869636
C	-4.339874	-3.462724	0.038867
H	-4.508182	-4.543149	0.066733
C	-4.737679	-2.666675	1.105210
H	-5.199185	-3.136726	1.978583
C	-4.528999	-1.282775	1.100828
C	-2.667938	-0.937330	-2.262809
H	-2.658839	0.156369	-2.155816
C	-3.250931	-1.291770	-3.636314
H	-2.701288	-0.764735	-4.436516
H	-4.315223	-1.016770	-3.711754
H	-3.172844	-2.374103	-3.840730
C	-1.208176	-1.402667	-2.188203
H	-0.568013	-0.842762	-2.886901
H	-1.112093	-2.470712	-2.453891
H	-0.814771	-1.402143	-1.141452
C	-4.846898	-0.494767	2.363431
H	-4.666005	0.565950	2.148287
C	-3.896550	-0.876640	3.508466
H	4.517249	-2.250562	2.355498
H	5.681204	-0.897336	2.318981
C	6.429389	-1.104668	-0.288783
H	7.241995	-1.811800	-0.050362
H	6.710043	-0.116521	0.113668
H	6.364290	-1.032226	-1.384123
C	2.455177	-3.618827	0.732384
H	2.748440	-4.680280	0.636237
H	1.357499	-3.559703	0.718890
H	2.786354	-3.261001	1.720473
C	2.594572	-3.325326	-1.768807
H	2.978285	-4.350300	-1.927923
H	2.968278	-2.687341	-2.588838
H	1.497959	-3.355049	-1.841917
C	4.109629	0.710661	0.321622
C	4.516399	1.520194	-0.761746
C	4.660847	2.893318	-0.549897
H	4.975429	3.537015	-1.376644
C	4.384509	3.461589	0.688572
H	4.499922	4.539307	0.836542
C	3.931262	2.659004	1.728142
H	3.672945	3.120391	2.685810
C	3.771114	1.278793	1.567492
C	4.707523	0.972521	-2.170567
H	4.642944	-0.123454	-2.114877
C	6.072490	1.344890	-2.763949
H	6.230441	0.818467	-3.721815
H	6.900526	1.080519	-2.085481
H	6.142207	2.427679	-2.970091
C	3.576247	1.427627	-3.105036
H	3.730697	1.015334	-4.118563
H	3.543504	2.529048	-3.187660
H	2.597598	1.084185	-2.731717
C	3.156181	0.481407	2.709441
H	3.129457	-0.573426	2.406403
C	1.700966	0.900354	2.966894

H	-4.131147	-0.279061	4.407369	H	1.267891	0.274619	3.767542
H	-2.846598	-0.687518	3.235296	H	1.083445	0.781135	2.062743
H	-3.999445	-1.943618	3.776664	H	1.640558	1.954767	3.292274
C	-6.306386	-0.658762	2.807852	C	3.974457	0.590341	4.003890
H	-6.535214	0.038190	3.633099	H	3.563727	-0.087505	4.772791
H	-6.501471	-1.680773	3.178610	H	3.938247	1.614316	4.416337
H	-7.017794	-0.462985	1.988081	H	5.035744	0.331793	3.852433

(DBB)Ru^0 (*singlet*)

E(scf) = -1968.56830185 a.u.

B	1.426424	-0.676632	1.124033
Ru	-0.001553	0.000612	-0.436625
B	-1.426367	0.676408	1.126573
C	-0.103676	1.458284	1.246991
H	-0.070393	2.547301	1.128226
C	-1.179802	-0.853305	1.207799
H	-1.981820	-1.577397	1.035362
C	0.104010	-1.458625	1.245867
H	0.070465	-2.547595	1.126708
C	1.180024	0.852985	1.206505
H	1.981793	1.577235	1.033498
C	2.587776	-1.318723	0.342913
N	3.741338	-0.704535	-0.081162
C	4.799649	-1.601765	-0.614338
C	3.997754	-2.876526	-0.874889
H	4.591603	-3.786499	-0.688213
H	3.697732	-2.897743	-1.936287
C	2.729947	-2.818934	0.005882
C	5.925216	-1.835418	0.404855
H	6.650332	-2.557381	-0.008604
H	5.548699	-2.242367	1.355412
H	6.465683	-0.899187	0.613450
C	5.442587	-1.073225	-1.898977
H	6.229914	-1.774264	-2.224670
H	5.909593	-0.085600	-1.744331
H	4.711058	-0.994138	-2.715901

(DBB)Ru^0 (*triplet*)

E(scf) = -1968.56460975 a.u.

B	-1.421035	0.553491	1.009035
Ru	-0.115646	0.222182	-0.733554
B	1.347814	-0.690364	0.670509
C	0.067258	-1.551209	0.872472
H	0.073121	-2.641369	0.737875
C	1.161020	0.817249	0.979306
H	1.975545	1.546090	0.915007
C	-0.120117	1.383102	1.159574
H	-0.125721	2.477007	1.226335
C	-1.200953	-0.993012	1.014752
H	-2.021574	-1.714634	0.950571
C	-2.675091	1.278710	0.447134
N	-3.842708	0.704651	0.046785
C	-4.951767	1.644833	-0.287511
C	-4.177704	2.948685	-0.472212
H	-4.753979	3.825772	-0.135037
H	-3.969471	3.090824	-1.546176
C	-2.839973	2.804520	0.287098
C	-5.976497	1.748477	0.851584
H	-6.731006	2.510369	0.591326
H	-5.512873	2.045205	1.804351
H	-6.502131	0.792729	0.998700
C	-5.708826	1.251896	-1.557065
H	-6.528802	1.971098	-1.722616
H	-6.151347	0.244848	-1.472970
H	-5.060220	1.280646	-2.443852

C	2.904867	-3.617084	1.311703	C	-2.896558	3.461265	1.678622
H	3.119398	-4.678539	1.089649	H	-3.102425	4.542735	1.582257
H	1.990136	-3.566254	1.923617	H	-1.940303	3.333602	2.209778
H	3.730517	-3.220818	1.925610	H	-3.684156	3.017527	2.309887
C	1.593460	-3.443400	-0.823733	C	-1.768106	3.503357	-0.570706
H	1.923429	-4.432087	-1.194862	H	-2.135514	4.508924	-0.849101
H	1.354015	-2.803962	-1.689584	H	-1.575230	2.932280	-1.495866
H	0.668211	-3.596414	-0.253348	H	-0.808494	3.628878	-0.054858
C	3.950521	0.706964	-0.015211	C	-4.028606	-0.711876	-0.058936
C	3.531167	1.502467	-1.107513	C	-3.681413	-1.344274	-1.275459
C	3.741750	2.881526	-1.053662	C	-3.851466	-2.726152	-1.379681
H	3.419865	3.510905	-1.888923	H	-3.580299	-3.234603	-2.309740
C	4.338552	3.474057	0.054054	C	-4.346417	-3.472221	-0.315820
H	4.497798	4.555897	0.080210	H	-4.473607	-4.553998	-0.415372
C	4.706268	2.687851	1.138141	C	-4.657687	-2.840450	0.881032
H	5.135961	3.166704	2.022872	H	-5.012547	-3.439729	1.724496
C	4.506731	1.302672	1.136591	C	-4.491883	-1.460567	1.042424
C	2.783728	0.925268	-2.302137	C	-3.073279	-0.596582	-2.456842
H	2.756610	-0.166380	-2.178918	H	-3.063862	0.473021	-2.202512
C	3.457693	1.256558	-3.641032	C	-3.879442	-0.784107	-3.749883
H	2.956183	0.721442	-4.467034	H	-3.484098	-0.129454	-4.546714
H	4.522824	0.975720	-3.646191	H	-4.947235	-0.551825	-3.610536
H	3.399141	2.336518	-3.864264	H	-3.812472	-1.824159	-4.114616
C	1.324270	1.401739	-2.339751	C	-1.612470	-1.005166	-2.702635
H	0.727909	0.808041	-3.049262	H	-1.103431	-0.282922	-3.362587
H	1.253268	2.457228	-2.660214	H	-1.539406	-1.997556	-3.182196
H	0.846206	1.466317	-1.318783	H	-1.067181	-1.165530	-1.729971
C	4.792343	0.529143	2.416290	C	-4.721826	-0.861347	2.423332
H	4.613936	-0.534017	2.210667	H	-4.584297	0.224288	2.344702
C	3.818863	0.928746	3.535708	C	-3.682070	-1.359218	3.438806
H	4.026523	0.336107	4.444418	H	-3.876841	-0.905273	4.426801
H	2.773553	0.748040	3.240122	H	-2.659366	-1.089829	3.132406
H	3.926291	1.996562	3.798474	H	-3.729781	-2.456586	3.557494
C	6.241341	0.696139	2.893566	C	-6.139836	-1.134236	2.944234
H	6.448561	0.009856	3.733357	H	-6.326650	-0.556872	3.866640
H	6.428852	1.722760	3.255291	H	-6.276593	-2.201857	3.191877

H	6.972318	0.488597	2.094512	H	-6.915879	-0.864774	2.208302
N	-3.740537	0.704099	-0.081104	N	3.781866	-0.671373	-0.268345
C	-2.586193	1.318060	0.343031	C	2.572721	-1.263732	-0.090384
C	-4.798330	1.601769	-0.614148	C	4.822340	-1.511852	-0.932574
C	-3.995583	2.875765	-0.875649	C	3.958315	-2.623845	-1.529962
H	-4.588900	3.786386	-0.690383	H	4.479111	-3.595464	-1.533238
H	-3.694965	2.895473	-1.936924	H	3.727737	-2.373082	-2.579374
C	-2.728172	2.818357	0.005664	C	2.645994	-2.663864	-0.719776
C	-5.923484	1.836884	0.405271	C	5.847307	-2.035034	0.083980
H	-6.647774	2.559818	-0.007962	H	6.580583	-2.677744	-0.432055
H	-5.546324	2.243172	1.355837	H	5.385395	-2.627166	0.886820
H	-6.465118	0.901323	0.613865	H	6.395720	-1.196255	0.542235
C	-5.442439	1.073250	-1.898265	C	5.602091	-0.770454	-2.019219
H	-6.229717	1.774511	-2.223609	H	6.344845	-1.459405	-2.456374
H	-5.909763	0.085862	-1.743038	H	6.145986	0.094893	-1.605404
H	-4.711564	0.993583	-2.715725	H	4.949171	-0.425280	-2.833712
C	-2.903532	3.616984	1.311138	C	2.712808	-3.729223	0.393204
H	-3.118652	4.678247	1.088687	H	2.862985	-4.729778	-0.051274
H	-1.988741	3.566958	1.923042	H	1.784471	-3.748158	0.983294
H	-3.728855	3.220534	1.925323	H	3.542212	-3.541280	1.093866
C	-1.591564	3.442950	-0.823666	C	1.496118	-2.999314	-1.682296
H	-1.921396	4.431727	-1.194707	H	1.733447	-3.937230	-2.218910
H	-1.352051	2.803586	-1.689542	H	1.367963	-2.194748	-2.427154
H	-0.666369	3.595834	-0.253119	H	0.535830	-3.131174	-1.166138
C	-3.950200	-0.707209	-0.015124	C	4.136022	0.600920	0.290183
C	-3.531194	-1.502864	-1.107463	C	4.079445	1.752409	-0.527575
C	-3.741841	-2.881908	-1.053625	C	4.472981	2.975775	0.022237
H	-3.420140	-3.511311	-1.888943	H	4.438065	3.878173	-0.595102
C	-4.338617	-3.474390	0.054141	C	4.880649	3.074796	1.346622
H	-4.497990	-4.556215	0.080306	H	5.182539	4.042144	1.758396
C	-4.706340	-2.688108	1.138162	C	4.872221	1.944301	2.153612
H	-5.136262	-3.166884	2.022831	H	5.151231	2.037823	3.207079
C	-4.506640	-1.302945	1.136583	C	4.491980	0.695021	1.654186
C	-2.784255	-0.925452	-2.302257	C	3.527291	1.749118	-1.948941
H	-2.757747	0.166209	-2.179128	H	3.304511	0.706891	-2.221442
C	-3.458340	-1.257121	-3.641019	C	4.522636	2.327394	-2.966132

H	-2.957243	-0.721830	-4.467160
H	-4.523580	-0.976659	-3.645893
H	-3.399429	-2.337069	-3.864215
C	-1.324670	-1.401161	-2.340068
H	-0.728736	-0.807659	-3.050109
H	-1.253306	-2.456817	-2.659946
H	-0.845742	-1.465541	-1.319398
C	-4.792690	-0.529256	2.416071
H	-4.613803	0.533799	2.210345
C	-3.820056	-0.929057	3.536145
H	-4.028031	-0.336186	4.444637
H	-2.774506	-0.748787	3.241163
H	-3.928037	-1.996788	3.799044
C	-6.242042	-0.695760	2.892452
H	-6.449664	-0.009218	3.731934
H	-6.430083	-1.722253	3.254283
H	-6.972405	-0.488216	2.092838

$Fe(CO)_2^{2+}$ (*singlet*)

E(scf) = -1489.09350162 a.u.

O	2.276888	-1.167359	0.000042
O	-2.276889	-1.167358	-0.000040
Fe	0.000000	0.914912	0.000000
C	1.450475	-0.425830	-0.000088
C	-1.450473	-0.425832	0.000084

$Fe(CO)_2^{2+}$ (*triplet*)

E(scf) = -1489.12840772 a.u.

O	2.219322	-1.230773	0.000066
O	-2.219321	-1.230774	-0.000063
Fe	0.000000	0.968485	0.000001
C	1.422791	-0.457353	-0.000138
C	-1.422790	-0.457354	0.000130

$Fe(CO)_2^{2+}$ (*quintuplet*)

E(scf) = -1489.17004815 a.u.

H	4.156380	2.162477	-3.994828
H	5.523489	1.875339	-2.881933
H	4.638575	3.417494	-2.832070
C	2.200203	2.516814	-2.040936
H	1.827261	2.503075	-3.080704
H	2.318602	3.572608	-1.737201
H	1.427540	2.057631	-1.393432
C	4.394108	-0.469833	2.631544
H	4.120701	-1.368774	2.063373
C	3.271334	-0.236517	3.653965
H	3.175953	-1.115277	4.316195
H	2.303553	-0.075710	3.153332
H	3.486347	0.642101	4.288436
C	5.718054	-0.748268	3.356170
H	5.631242	-1.664789	3.965927
H	5.982853	0.076397	4.041290
H	6.558814	-0.882944	2.656386

$Ru(CO)_2^{2+}$ (*singlet*)

E(scf) = -320.445402691 a.u.

Ru	-0.000044	-0.703083	-0.000001
O	2.204613	1.432938	0.000003
O	-2.204604	1.432941	-0.000018
C	1.395472	0.667228	-0.000006
C	-1.395163	0.667544	0.000037

$Ru(CO)_2^{2+}$ (*triplet*)

E(scf) = -320.456216631 a.u.

Ru	-0.000797	-0.754310	-0.000001
O	2.123166	1.528715	0.000004
O	-2.120110	1.531975	-0.000018
C	1.354070	0.724341	-0.000008
C	-1.352297	0.726344	0.000037

$Ru(CO)_2^{2+}$ (*quintuplet*)

E(scf) = -320.443886698 a.u.

O	-2.606170	-1.142762	0.000016
O	2.606334	-1.142620	0.000021
Fe	-0.000068	0.916322	0.000006
C	-1.728255	-0.461795	-0.000032
C	1.728330	-0.461758	-0.000041

$Fe(CO)_2^{2+}$ (*hextuplet*)

E(scf) = -1488.98906691 a.u.

O	2.351901	-1.396179	0.000236
O	-2.353203	-1.394691	0.000144
Fe	0.000593	1.177453	-0.000150
C	1.492283	-0.691022	0.000100
C	-1.493116	-0.690113	0.000045

$OsCO_2^{2+}$ (*singlet*)

E(scf) = -316.240695965 a.u.

Os	0.000000	0.482131	0.000000
O	2.213606	-1.635500	0.000023
O	-2.213606	-1.635500	-0.000022
C	1.398099	-0.872830	-0.000048
C	-1.398095	-0.872834	0.000047

$OsCO_2^{2+}$ (*triplet*)

E(scf) = -316.253275149 a.u.

Os	-0.000001	0.513320	0.000000
O	2.115005	-1.738129	-0.000003
O	-2.114998	-1.738137	0.000003
C	1.342706	-0.933513	0.000007
C	-1.342704	-0.933516	-0.000007

$OsCO_2^{2+}$ (*quintuplet*)

E(scf) = -316.218263761 a.u.

Os	0.000000	0.383280	0.000000
O	2.842670	-1.295413	-0.000017
O	-2.842669	-1.295413	0.000016
C	1.903199	-0.700224	0.000033

Ru	0.000005	-0.344333	0.000008
O	3.239160	0.692906	-0.000030
O	-3.239178	0.692898	0.000117
C	2.186131	0.338684	0.000057
C	-2.186145	0.338685	-0.000233

$(DBB)Fe^{2+}$ (*singlet*)

E(scf) = -3136.79396937 a.u.

B	1.454492	-0.029045	0.929806
Fe	0.048622	-0.470613	-0.789826
B	-1.415858	0.828796	0.367242
C	-0.220140	1.539141	-0.226850
H	-0.347686	2.324626	-0.981708
C	-1.076850	-0.530399	0.989173
H	-1.841503	-1.302758	1.132933
C	0.248326	-0.915932	1.264783
H	0.377526	-1.953886	1.595914
C	1.119945	1.130594	0.014710
H	1.890175	1.641755	-0.570138
C	2.940407	-0.494603	1.346181
N	4.008667	-0.426859	0.606496
C	5.259826	-1.057832	1.211870
C	4.687074	-1.629788	2.519332
H	5.360465	-1.448995	3.370841
H	4.567286	-2.721786	2.428713
C	3.304663	-0.981016	2.737020
C	6.379091	-0.044902	1.451068
H	7.248872	-0.593104	1.847101

C	-1.903198	-0.700225	-0.000033

(DBB)Ru^{2+} (*singlet*)

E(scf) = -1968.16480580 a.u.

B	1.437367	-0.725433	-0.539181
Ru	0.000080	1.042368	0.001650
B	-1.437395	-0.726979	0.537307
C	-0.214184	-0.569026	1.439894
H	-0.322684	-0.302609	2.497783
C	-1.123701	-0.559862	-0.946392
H	-1.900802	-0.304205	-1.673967
C	0.214137	-0.564736	-1.441313
H	0.322644	-0.295088	-2.498375
C	1.123676	-0.562642	0.944972
H	1.900774	-0.309177	1.673328
C	2.916452	-0.838185	-1.177914
N	4.013234	-0.322400	-0.705207
C	5.244289	-0.493061	-1.591730
C	4.637623	-1.229274	-2.796347
H	5.273938	-2.065447	-3.123713
H	4.548666	-0.538421	-3.650389
C	3.234527	-1.712180	-2.380163
C	6.358290	-1.284297	-0.908951
H	7.220048	-1.313103	-1.594938
H	6.077540	-2.325248	-0.693366
H	6.690852	-0.795538	0.019303
C	5.787227	0.884429	-1.974219
H	6.649804	0.743121	-2.645562
H	6.137358	1.445159	-1.091815
H	5.043766	1.486549	-2.519278
C	3.247133	-3.160203	-1.834661
H	3.505874	-3.849580	-2.655827
H	2.254773	-3.449447	-1.447987
H	3.985248	-3.298563	-1.028106
C	2.266898	-1.639727	-3.565025
H	2.712430	-2.193195	-4.408800
H	6.115214	0.723982	2.191197
H	6.693098	0.440527	0.515008
C	5.789983	-2.148041	0.278408
H	6.693442	-2.585849	0.732615
H	6.075031	-1.738006	-0.704917
H	5.069225	-2.967544	0.136325
C	3.376298	0.295222	3.608816
H	3.691567	0.016818	4.628369
H	2.389282	0.783666	3.679211
H	4.096102	1.032178	3.217269
C	2.333722	-1.971029	3.386447
H	2.782675	-2.333297	4.326646
H	2.152573	-2.851928	2.747457
H	1.369770	-1.503243	3.639952
C	4.005904	0.167109	-0.714090
C	3.563892	-0.615248	-1.797183
C	3.514687	-0.015894	-3.058863
H	3.187743	-0.598480	-3.924907
C	3.885850	1.311699	-3.238945
H	3.854316	1.759562	-4.235485
C	4.292778	2.068701	-2.147725
H	4.560232	3.117773	-2.297616
C	4.352452	1.526720	-0.859114
C	3.082093	-2.053943	-1.656490
H	3.316211	-2.412591	-0.646224
C	3.766081	-3.005946	-2.646091
H	3.496692	-4.051724	-2.421947
H	4.862130	-2.915347	-2.583064
H	3.472288	-2.802374	-3.689461
C	1.553696	-2.128609	-1.805343
H	1.202475	-3.151869	-2.019093
H	1.211276	-1.487088	-2.635035
H	1.091689	-1.904567	-0.795963
C	4.714905	2.454606	0.295849
H	4.791810	1.850944	1.207337
C	3.626514	3.505815	0.567359

H	2.101492	-0.602424	-3.902511	H	3.979164	4.210620	1.339115
H	1.296854	-2.109235	-3.341644	H	2.692885	3.051841	0.936521
C	4.106473	0.397945	0.548083	H	3.394322	4.094364	-0.337338
C	3.799174	1.771278	0.567482	C	6.067461	3.146179	0.067935
C	3.939438	2.458693	1.775505	H	6.394338	3.651716	0.991886
H	3.715812	3.528413	1.819546	H	5.996501	3.918161	-0.717145
C	4.345061	1.800845	2.930828	H	6.857382	2.439219	-0.233126
H	4.458174	2.354670	3.866250	N	-3.980374	0.742961	-0.110969
C	4.589349	0.433249	2.897355	C	-2.900780	1.418845	0.153076
H	4.874036	-0.078626	3.819961	C	-5.206341	1.587399	-0.445547
C	4.473226	-0.305564	1.715056	C	-4.634844	3.008338	-0.304901
C	3.192666	2.502784	-0.621632	H	-5.304569	3.658188	0.278489
H	3.223457	1.834537	-1.491643	H	-4.526958	3.468736	-1.300120
C	3.934490	3.789359	-0.999186	C	-3.248565	2.883581	0.357997
H	3.486133	4.241854	-1.899940	C	-6.385573	1.324919	0.487941
H	4.995965	3.588906	-1.211842	H	-7.235016	1.932570	0.136671
H	3.891029	4.538852	-0.191287	H	-6.182338	1.617974	1.528214
C	1.697832	2.793274	-0.336590	H	-6.699518	0.270364	0.459012
H	1.478966	3.866763	-0.210115	C	-5.635378	1.283688	-1.882438
H	1.422339	2.329119	0.655155	H	-6.487468	1.934915	-2.136747
H	1.063688	2.454580	-1.200309	H	-5.965160	0.238043	-1.999838
C	4.675408	-1.816235	1.781500	H	-4.831977	1.497095	-2.605259
H	4.678328	-2.206400	0.756644	C	-3.316798	3.063083	1.893811
C	3.518416	-2.519442	2.509632	H	-3.598406	4.105272	2.119725
H	3.747682	-3.592862	2.619423	H	-2.337542	2.866035	2.362981
H	2.568875	-2.439265	1.956254	H	-4.063740	2.399342	2.359391
H	3.367980	-2.106168	3.522279	C	-2.275325	3.915475	-0.217862
C	6.012607	-2.195885	2.434111	H	-2.726806	4.915801	-0.106867
H	6.207388	-3.272383	2.295222	H	-2.090893	3.755134	-1.293609
H	5.999046	-2.007243	3.521004	H	-1.313766	3.925901	0.317575
H	6.862549	-1.637824	2.009759	C	-4.052516	-0.701661	-0.169036
N	-4.013273	-0.324377	0.704238	C	-3.669563	-1.353662	-1.356580
C	-2.916591	-0.841740	1.175430	C	-3.816787	-2.740672	-1.422892
C	-5.244428	-0.497682	1.590131	H	-3.538253	-3.274130	-2.336116
C	-4.638196	-1.238460	2.792181	C	-4.297970	-3.460406	-0.334443
H	-5.274531	-2.076010	3.115961	H	-4.416662	-4.544691	-0.403664

H	-4.549804	-0.550948	3.648963
C	-3.234831	-1.719583	2.374811
C	-6.358860	-1.285911	0.904583
H	-7.220588	-1.316785	1.590515
H	-6.078599	-2.326218	0.685271
H	-6.691262	-0.793665	-0.021883
C	-5.786642	0.878700	1.977602
H	-6.649421	0.735427	2.648266
H	-6.136273	1.442871	1.097192
H	-5.042918	1.478352	2.525018
C	-3.247031	-3.165852	1.824661
H	-3.505850	-3.857917	2.643543
H	-2.254543	-3.453718	1.437290
H	-3.984971	-3.301726	1.017514
C	-2.267528	-1.650623	3.560121
H	-2.713039	-2.207005	4.401990
H	-2.102643	-0.614337	3.900988
H	-1.297239	-2.118985	3.335455
C	-4.106386	0.399774	-0.546881
C	-3.798972	1.773123	-0.562283
C	-3.939088	2.464017	-1.768348
H	-3.715295	3.533822	-1.809317
C	-4.344739	1.809559	-2.925578
H	-4.457738	2.366094	-3.859405
C	-4.589214	0.441903	-2.896056
H	-4.874027	-0.067271	-3.820116
C	-4.473152	-0.300333	-1.715899
C	-3.192457	2.501180	0.628963
H	-3.223254	1.830437	1.497053
C	-3.934144	3.786760	1.010158
H	-3.485762	4.236637	1.912209
H	-4.995650	3.585867	1.222209
H	-3.890533	4.538515	0.204372
C	-1.697679	2.792405	0.344736
H	-1.478967	3.866194	0.220522
H	-1.422148	2.330449	-0.648032
C	-4.605723	-2.800998	0.849222
H	-4.944715	-3.383414	1.709459
C	-4.489913	-1.411250	0.967979
C	-2.936567	-0.637692	-2.481879
H	-2.966371	0.442816	-2.291204
C	-3.517656	-0.880243	-3.877042
H	-2.952528	-0.312378	-4.635643
H	-4.569736	-0.559474	-3.926896
H	-3.479059	-1.945853	-4.158529
C	-1.452682	-1.060390	-2.422019
H	-1.227607	-1.905320	-3.094869
H	-1.270538	-1.489770	-1.386215
H	-0.786526	-0.217742	-2.723259
C	-4.768643	-0.767122	2.322038
H	-4.778905	0.321073	2.185275
C	-3.652233	-1.066198	3.335315
H	-3.930532	-0.663939	4.324009
H	-2.695113	-0.601547	3.047826
H	-3.491913	-2.152446	3.450581
C	-6.129350	-1.181673	2.899544
H	-6.372953	-0.556065	3.774334
H	-6.120534	-2.229513	3.244534
H	-6.946549	-1.077604	2.167822

4

E(scf) = -4200.3797249501 a.u.

Fe	-0.747050	0.176385	0.325087
P	0.940282	-0.556835	-0.84256
P	-1.41333	1.210076	-1.475132
P	-1.644278	-1.800874	0.040894
H	-1.158076	-0.482658	-3.210589
H	-0.650527	1.096578	-3.838324
C	0.77886	-2.390027	-1.107534
H	1.397213	-2.736039	-1.953592
H	1.150438	-2.885972	-0.194587
C	-0.704461	-2.71815	-1.284491

H	-1.063606	2.451968	1.207758
C	-4.675572	-1.810780	-1.786658
H	-4.678797	-2.203823	-0.762898
C	-3.518546	-2.512143	-2.516526
H	-3.748041	-3.585184	-2.629501
H	-2.569138	-2.433818	-1.962660
H	-3.367738	-2.095952	-3.527924
C	-6.012704	-2.188378	-2.440581
H	-6.207607	-3.265261	-2.304896
H	-5.998986	-1.996532	-3.526909
H	-6.862647	-1.631496	-2.014681

5

E(scf) = -3096.25506943a.u.

C	-1.374043	-1.391227	0.262245
Fe	0.153796	-0.000803	0.002662
P	1.949145	-1.422287	0.580534
P	1.939121	-0.604194	-1.420384
C	-1.392664	1.41456	-0.246359
C	-1.405566	-0.914241	-1.078545
P	1.958119	1.394764	-0.603791
C	-1.359285	-0.468976	1.352587
C	-1.346726	0.936481	1.094732
P	1.971099	0.577499	1.397287
C	-1.339129	0.489276	-1.33144
C	-1.31521	-2.876002	0.527299
C	-1.454503	2.901161	-0.518517
H	-1.948288	3.105526	-1.479651
H	-2.039726	3.415752	0.258277
H	-0.448907	3.354327	-0.550587
H	-0.774664	-3.405337	-0.269787
H	-0.786364	-3.099847	1.463149
H	-2.335074	-3.299056	0.593223
C	-1.182563	0.994934	-2.745025
H	-0.623349	0.276715	-3.361353
H	-2.165436	1.171118	-3.219784
H	-0.889349	-3.804644	-1.22449
H	-1.068334	-2.38342	-2.271154
C	2.704126	-0.311764	-0.413329
C	3.227473	0.989314	-0.436826
H	2.586142	1.833972	-0.702914
C	4.555355	1.230792	-0.094281
H	4.940545	2.253982	-0.11337
C	5.383665	0.174724	0.284945
H	6.425125	0.363731	0.560368
C	4.874177	-1.121429	0.318174
H	5.513476	-1.956126	0.619371
C	3.544645	-1.362956	-0.028223
H	3.174259	-2.389463	0.011873
C	-0.864305	2.956431	-1.66991
H	-1.36487	3.584954	-0.914755
C	-3.168399	1.350516	-1.997535
H	-3.62161	0.35304	-2.104888
C	-1.480477	-3.00245	1.427316
H	-0.444053	-2.991597	1.80341
C	-3.393267	-2.079736	-0.449001
H	-3.58092	-1.658656	-1.449593
C	0.85456	0.105396	-2.591777
H	1.320446	-0.601297	-3.29997
H	1.462777	1.025826	-2.614595
C	-0.59562	0.432634	-2.957844
H	-3.720439	1.893925	-1.21262
H	-3.265041	1.894419	-2.953035
H	-1.10381	3.338472	-2.677423
H	0.2218	3.024096	-1.495842
H	-4.050042	-1.566336	0.272908
H	-3.642149	-3.1548	-0.465573
H	-1.74604	-4.02227	1.099096
H	-2.142235	-2.702464	2.255839
P	0.48635	1.889226	1.446641
P	-1.674106	2.031891	1.517052
P	-1.78282	0.129858	2.476087

H	-0.614311	1.935802	-2.767298	P	0.379942	-0.065545	2.411958
C	-1.472093	-1.894909	-2.227944				
H	-1.942648	-1.438749	-3.110837				
H	-0.469186	-2.247546	-2.523627				
H	-2.079683	-2.771764	-1.958163				
C	-1.347163	-0.998205	2.769251				
H	-1.689396	-0.243036	3.489439				
H	-2.023821	-1.86159	2.864576				
H	-0.337289	-1.323233	3.073001				
C	-1.238534	1.924449	2.231449				
H	-2.241332	2.214917	2.595993				
H	-0.666563	1.510568	3.072387				
H	-0.71005	2.835755	1.918735				

References

[1] M. Arrowsmith, J. Böhnke, H. Braunschweig. M. A. Celik, C. Claes, W. C. Ewing, I. Krummenacher, K.Lubitz, C. Schneider, *Angew. Chem. Int. Ed.* **2016**, *55*, 11271–11275.

[2] O. J. Scherer, H. Sitzmann, G. Wolmershäuser, *J. Organomet. Chem.* **1986**, *309*, 77–86.

[3] G. Sheldrick, *Acta Cryst.* **2015**, *A71*, 3–8.

[4] G. Sheldrick, *Acta Cryst.* **2008**, *A64*, 112–122.

[5] A. L. Spek, Platon. Utrecht University, Utrecht, The Netherlands, **1980**–**2021**.

[6] A. L. Spek, *Acta Cryst.* **2015**, *C71*, 9–18.

[7] M. J. Frisch, G. W. Trucks, H. B. Schlegel, G. E. Scuseria, M. A. Robb, J. R. Cheeseman, G. Scalmani, V. Barone, G. A. Petersson, H. Nakatsuji, X. Li, M. Caricato, A. V. Marenich, J. Bloino, B. G. Janesko, R. Gomperts, B. Mennucci, H. P. Hratchian, J. V. Ortiz, A. F. Izmaylov, J. L. Sonnenberg, D. Williams-Young, F. Ding, F. Lipparini, F. Egidi, J. Goings, B. Peng, A. Petrone, T. Henderson, D. Ranasinghe, V. G. Zakrzewski, J. Gao, N. Rega, G. Zheng, W. Liang, M. Hada, M. Ehara, K. Toyota, R. Fukuda, J. Hasegawa, M. Ishida, T. Nakajima, Y. Honda, O. Kitao, H. Nakai, T. Vreven, K. Throssell, J. A. Montgomery, Jr., J. E. Peralta, F. Ogliaro, M. J. Bearpark, J. J. Heyd, E. N. Brothers, K. N. Kudin, V. N. Staroverov, T. A. Keith, R. Kobayashi, J. Normand, K. Raghavachari, A. P. Rendell, J. C. Burant, S. S. Iyengar, J. Tomasi, M. Cossi, J. M. Millam, M. Klene, C. Adamo, R. Cammi, J. W. Ochterski, R. L. Martin, K. Morokuma, O. Farkas, J. B. Foresman, D. J. Fox, Gaussian 16, Revision C.01. Gaussian, Inc., Wallingford CT, **2016**.

[8] J.-D. Chai, M. Head-Gordon, *Phys. Chem. Chem. Phys.* **2008**, *10*, 6615–6620.

[9] F. Weigend, R. Ahlrichs, *Phys. Chem. Chem. Phys.* **2005**, *7*, 3297–3305.

[10] S. F. Boys, F. Bernardi, *Mol. Phys.* **1970**, *19*, 553–566.

[11] S. Simon, M. Duran, J. J. Dannenberg, *J. Chem. Phys.* **1996**, *105*, 11024–11031.

[12] P. Salvador, M. Duran, *J. Chem. Phys.* **1999**, *111*, 4460–4465.

[13] Z. Chen, C. S. Wannere, C. Corminboeuf, R. Puchta, P. v. R. Schleyer, *Chem. Rev*. **2005**, *105*, 3842–3888.

[14] P. v. R. Schleyer, C. Maerker, A. Dransfeld, H. Jiao, N. J. R. van Eikema Hommes, *J. Am. Chem. Soc.* **1996**, *118*, 6317–6318.

[15] J. O. C. Jimenez-Halla, E. Matito, J. Robles, M. Solà, *J. Organomet. Chem*. **2006**, *691*, 4359–4366.

[16] M. Giambiagi, M. S. de Giambiagi, K. C. Mundim, *Struct. Chem.* **1990**, *1*, 423–427.

[17] K. Wolinski, J. F. Hilton, P. Pulay, *J. Am. Chem. Soc.* **1990**, *112*, 8251–8260.

[18] T. Lu, F. Chen, *J. Comput. Chem.* **2012**, *33*, 580–592.

[19] G. Knizia, *J. Chem. Theory Comput.* **2013**, *9*, 4834–4843.

[20] G. Knizia, J. E. M. N. Klein, *Angew. Chem. Int. Ed.* **2015**, *54*, 5518–5522.

[21] E. D. Glendening, J. K. Badenhoop, A. E. Reed, J. E. Carpenter, J. A. Bohmann, C. M. Morales, P. Karafiloglou, C. R. Landis, F. Weinhold, NBO 7.0., Theoretical Chemistry Institute, University of Wisconsin, Madison, WI, **2019**.

[22] E. Ramos-Cordoba, V. Postils, P. Salvador, *J. Chem. Theory Comput.* **2015**, *11*, 1501–1508.

[23] A. Cavaillé, N. Saffon-Merceron, N. Nebra, M. Fustier-Boutignon, N. Mézailles, *Angew. Chem. Int. Ed.* **2018**, *57*, 1874–1878.

[24] G. Frenking, I. Fernández, N. Holzmann, S. Pan, I. Krossing, M. Zhou, *JACS Au* **2021**, *1*, 623–645.

[25] Z. Chen, C. S. Wannere, C. Corminboeuf, R. Puchta, P. v. R. Schleyer, *Chem. Rev.* **2005**, *105*, 3842–3888.

[26] a) K. Wolinski, J. F. Hilton, P. Pulay, *J. Am. Chem. Soc.* **1990**, *112*, 8251–8260; b) R. Ditchfield, *Mol. Phys.* **1974**, *27*, 789–807; c) J. R. Cheeseman, G. W. Trucks, T. A. Keith, M. J. Frisch, *J. Chem. Phys.* **1996**, *104*, 5497–5509.

[27] a) A. Stanger, *J. Org. Chem.* **2006**, *71*, 883–893; b) J. O. C. Jimenez-Halla, E. Matito, J. Robles, M. Solà, *J. Organomet. Chem.* **2006**, *691*, 4359–4366.

[28] F. Feixas, J. O. C. Jiménez-Halla, E. Matito, J. Poater, M. Solà, *Pol. J. Chem.* **2007**, *81*, 783–797

[29] P. Bultinck, M. Rafat, R. Ponec, B. van Gheluwe, R. Carbó-Dorca, P. Popelier, *J Phys Chem A* **2006**, *110*, 7642.

[30] a) F. Feixas, E. Matito, J. Poater, M. Solà, *J. Comput. Chem.* **2008**, *29*, 1543–1554; b) M. Solà, F. Feixas, J. O. C. Jiménez-Halla, E. Matito, J. Poater, *Symmetry* **2010**, *2*, 1156–1179.
